# *N*-Aryl-*S*-aryl-2-mercaptoacetamide Derivatives Effectively Inhibit Mushroom and Cellular Tyrosinase Activities, Melanin Production, and Pigmentation in Zebrafish Larvae: Regarding Copper Ion Chelation

**DOI:** 10.3390/molecules31030422

**Published:** 2026-01-26

**Authors:** Hee Jin Jung, Hye Jin Kang, Hyeon Seo Park, Minchang Kim, Hyunju Lee, Hyunhee Ju, Yeonsoo Jeong, Yujin Park, Hae Young Chung, Hyung Ryong Moon

**Affiliations:** 1 Department of Manufacturing Pharmacy, College of Pharmacy, Pusan National University, Busan 46241, Republic of Korea; hjjung2046@pusan.ac.kr (H.J.J.); dirgowls22@pusan.ac.kr (H.J.K.); gustj6956@pusan.ac.kr (H.S.P.); pawky0106@pusan.ac.kr (M.K.); lhj6384770@pusan.ac.kr (H.L.); hyunh@pusan.ac.kr (H.J.); jysoo627@pusan.ac.kr (Y.J.); 2 Research Institute for Drug Development, Pusan National University, Busan 46241, Republic of Korea; 3 Department of Medicinal Chemistry, New Drug Development Center, Daegu-Gyeongbuk Medical Innovation Foundation, Daegu 41061, Republic of Korea; pyj1016@kmedihub.re.kr; 4 Department of Pharmacy, College of Pharmacy, Pusan National University, Busan 46241, Republic of Korea; hyjung@pusan.ac.kr

**Keywords:** tyrosinase, melanin, mercaptoacetamide, zebrafish, copper chelation, depigmentation

## Abstract

In this study, we designed and synthesized 11 *N*-aryl-*S*-aryl-2-mercaptoacetamide derivatives as new tyrosinase inhibitors (TYRIs). Experiments with pyrocatechol violet confirmed that four derivatives showed copper-chelating abilities similar to or superior to those of well-known copper-chelating TYRIs like kojic acid (KA) and *N*-phenylthiourea. However, these four derivatives showed little or no inhibition of mushroom TYR (mTYR) activity and melanin production in B16F10 cells. Instead, derivatives with low copper chelation ability exhibited potent inhibitory effects on mTYR activity and melanin production in B16F10 cells. These findings suggest that the results of metal ion chelation by inhibitors in an enzyme-free environment do not always match those under metalloenzyme conditions because of the interactions between inhibitors and amino acid residues around the metalloenzyme active site. Owing to their favorable interactions with amino acids in the mTYR active site, two of the derivatives inhibited mTYR more effectively than KA. Probably for the same reason, three derivatives inhibited B16F10 cellular TYR more effectively than KA, and one derivative inhibited pigment production in zebrafish larvae much better than KA. This last derivative, which effectively exhibits TYR-inhibitory activity and suppresses melanin production in several species, is considered a promising compound for use as a TYRI in various fields.

## 1. Introduction

Melanin plays a vital role in determining the pigmentation of hair, skin, feathers, and eyes. Changes in melanin content give rise to various symptoms and diseases [1,2]; hyperpigmentation, caused by excess melanin production, results in melasma, freckles, and solar melanosis [3], while hypopigmentation, caused by less than normal melanin production, results in vitiligo, albinism, and idiopathic hypopigmentation [4]. Furthermore, malignant melanoma is associated with melanocytes, and an increase in melanocyte content in the substantia nigra of the brain can lead to neurological disorders, such as Parkinson’s disease [5,6]. Melanin is found in a variety of organisms, ranging from humans to plants and bacteria, and is involved in a variety of functions, including protecting the skin from ultraviolet rays, removing free radicals, determining the color of skin, hair, or feathers, regulating temperature, maintaining nervous system function, and cuticular hardening [7].

Melanin is produced in melanosomes within melanocytes via various non-enzymatic and enzymatic reactions involving tyrosinase (TYR), tyrosinase-related protein (TRP)-1, and TRP-2. Among these enzyme-related reactions, the enzymatic reaction involving TYR is the rate-limiting step, and thus plays the most crucial role in melanin biosynthesis [2,8]. TYR is a metalloenzyme containing two Cu^2+^ ions inside its active site and is a multifunctional enzyme that acts as a monophenolase, which oxidizes monophenol to catechol (*o*-dihydroxyphenol), and diphenolase, which oxidizes catechol to the corresponding *o*-quinone. In melanin biosynthesis, TYR participates in the conversion of l-tyrosine, l-DOPA, and 5,6-dihydroxyindole into l-DOPA, dopaquinone, and indole-5,6-quinone, respectively [9,10,11]. Owing to its rate-limiting properties, TYR is considered a promising target for the control of melanogenesis. Therefore, TYR inhibitors (TYRIs), including kojic acid (KA), ascorbic acid, and citric acid, have been used as skin-whitening agents, for hyperpigmentation treatments, and also to prevent the browning of crops and improve their appearance, thereby extending their shelf life [12]. Moreover, recent studies have shown that TYR regulation may be a potential target for the prevention and treatment of Parkinson’s disease [13,14].

**Figure 1:**
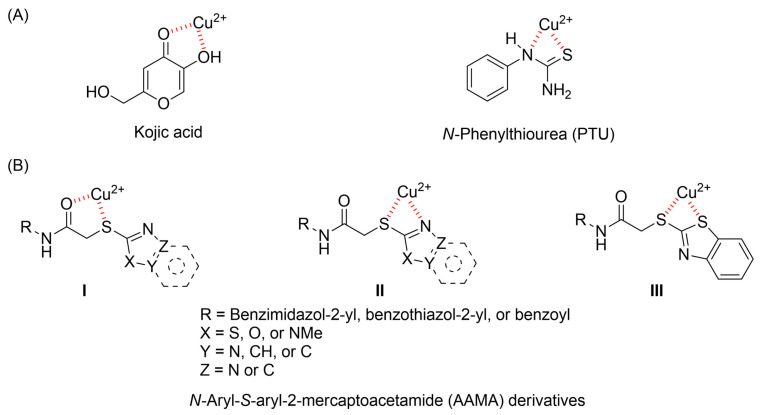
Copper ion chelation by (**A**) kojic acid (KA), *N*-phenylthiourea (PTU), and (**B**) *N*-aryl-*S*-aryl-2-mercaptoacetamide (AAMA) derivatives with three plausible copper-chelation modes.

Diverse TYRI applications have inspired the design and synthesis of novel TYRIs. This study was aimed at identifying novel TYRIs. KA and *N*-phenylthiourea (PTU) are well-known copper-ion-chelating TYRIs (Figure 1A). KA chelates copper ions using the oxygen atoms of its carbonyl and 5-hydroxyl groups [15,16], whereas PTU chelates copper ions using the sulfur atom of its thiocarbonyl group and the nitrogen atom of its amino group [17,18]. 2-Mercaptoacetamide derivatives have shown various biological effects, including antibacterial [19,20,21], antiparasitic [22], anti-melanogenic [23], antiviral [24], and anti-histone deacetylase [25,26,27] activities. The target compounds designed in this study, viz., *N*-aryl-*S*-aryl-2-mercaptoacetamide (AAMA) derivatives, were expected to chelate copper ions in one of three modes (I, II, or III), as shown in Figure 1B. Therefore, AAMA derivatives were synthesized and evaluated for their copper-ion-chelating efficacy. We also investigated their inhibitory activities on various types of TYR, melanin production in melanocytes, and pigmentation in zebrafish larvae (ZFL).

## 2. Results and Discussion

### 2.1. Preparation of AAMA Derivatives

**Scheme 1:**
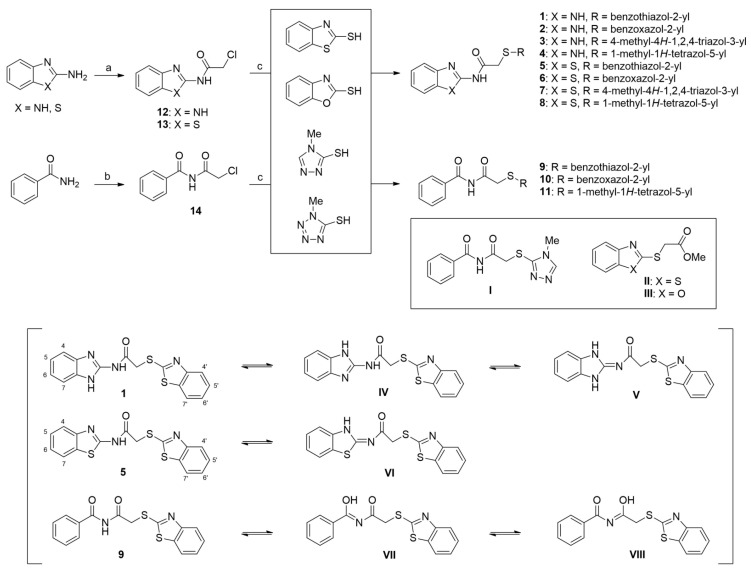
Syntheses of the target compounds, *N*-aryl-*S*-aryl-2-mercaptoacetamide derivatives **1**–**11**, and tautomeric structures of representative derivatives **1**, **5**, and **9**. Reagents and conditions: (a) 2-chloroacetyl chloride, acetone, reflux, 2 h for **12** and 7 h for **13**, 65% for **12** and 90% for **13**; (b) 2-chloroacetyl chloride, reflux, 50 min, 58%; (c) NaOMe, MeOH, 20 °C, 5–21 h, 29–99%.

The target compounds, i.e., AAMA derivatives **1**–**11**, were synthesized from three starting materials, viz., 2-aminobenzo[*d*]imidazole, 2-aminobenzo[*d*]thiazole, and benzamide via a two-step reaction (Scheme 1). Refluxing 2-aminobenzo[*d*]imidazole and 2-aminobenzo[*d*]thiazole with 2-chloroacetyl chloride in acetone yielded two 2-chloroacetamides, **12** (65%) and **13** (90%), respectively [28,29], and refluxing benzamide in 2-chloroacetyl chloride, used as both a reagent and a solvent, afforded *N*-(2-chloroacetyl)benzamide (**14**) in 58% yield [28]. Condensation reactions of **12** and **13** with four mercapto compounds (2-mercaptobenzo[*d*]thiazole, 2-mercaptobenzo[*d*]oxazole, 4-methyl-4*H*-1,2,4-triazole-3-thiol, and 1-methyl-1*H*-tetrazole-5-thiol) in the presence of NaOMe afforded the target compounds **1**–**8** in 40–99% yields. The coupling reaction of **14** with the mercapto compounds 2-mercaptobenzo[*d*]thiazole, 2-mercaptobenzo[*d*]oxazole, and 1-methyl-1*H*-tetrazole-5-thiol in the presence of NaOMe produced the target compounds **9**–**11** in 29–79% yields. However, under the same reaction conditions, the coupling of **14** with 4-methyl-4*H*-1,2,4-triazole-3-thiol did not produce the desired target compound (**I**); instead, benzamide was obtained as the main product. Additionally, the coupling reactions afforded **9** and **10** in only 29% and 49% yields, respectively, owing to the formation of byproducts **II** (45%) and **III** (31%), respectively.

In ^1^H NMR spectra, the protons at positions 4 and 7 and at positions 5 and 6 of the benzimidazole ring in derivatives **1**–**4** appeared at 7.47–7.41 ppm and 7.12–7.07 ppm, respectively. As shown in Figure 1, the benzimidazole moiety exists in three tautomers (**1**, **IV**, and **V**). Therefore, the proton at position 4 is identical to the proton at position 7, and the proton at position 5 is identical to the proton at position 6 [30]. Derivatives **2**–**4**, like derivative **1**, contain a benzimidazole moiety that quickly equilibrates with their tautomers. Derivative **5** contains two benzothiazole rings. Among the two benzothiazole rings, the one on the left is involved in the formation of tautomers (**5** and **VI**). Due to the presence of tautomer **VI**, the protons of the left benzothiazole ring appeared upfield in ^1^H-NMR spectrum compared to those of the right benzothiazole ring [31]. In derivative **6**, the protons of the benzothiazole ring appeared downfield compared to the protons of the benzoxazole ring. This is thought to be due to oxygen donating electrons to the benzene ring more effectively than sulfur. The protons at the 5-position of the 4-methyl-1,2,4-triazole ring of derivatives **3** and **7** appeared at 8.56 and 8.57 ppm, respectively, in the ^1^H NMR spectra, and the carbons at the 5-position of the 4-methyl-1,2,4-triazole ring appeared at 146.8 and 146.9 ppm, respectively, in the ^13^C NMR spectra. This confirmed the introduction of the 4-methyl-1,2,4-triazole ring. The imide group of derivative **9** is also in equilibrium with tautomers (**VII** and **VIII**). In derivatives **9**–**11**, the protons at positions 2 and 6 of the phenyl ring appeared most downfield due to the anisotropic effect [32]. Next, the proton at position 4, and then the protons at positions 3 and 5 appeared downfield. In all derivatives **1**–**11**, the α-methylene of sulfur appeared at 4.14–4.70 ppm and 34.2–38.3 ppm in the ^1^H and ^13^C NMR spectra, respectively.

### 2.2. Inhibition Activity of AAMA Derivatives Against Mushroom TYR (mTYR)

The TYR-inhibitory activity of the AAMA derivatives was assessed using mTYR with l-DOPA and l-tyrosine substrates. The half-maximal inhibitory concentration (IC_50_) values of AAMA derivatives were obtained using sets of three concentrations (5, 25, and 125 μM and 1, 5, and 25 μM). KA, a positive control, was tested at concentrations of 2, 10, and 50 μM.

The IC_50_ values of the AAMA derivatives are listed in Table 1. For derivatives with IC_50_ values exceeding 125 μM, specific IC_50_ values were not calculated. Eight of the 11 derivatives showed IC_50_ values exceeding 125 μM for both substrates. KA exhibited IC_50_ values of 31 ± 2 and 36.9 ± 1.5 μM with l-tyrosine and l-DOPA, respectively. Derivative **10** showed an IC_50_ value exceeding 125 μM in the presence of l-tyrosine but showed an IC_50_ value of 16.9 ± 0.7 μM in the presence of l-DOPA, which was two times lower than that of KA. Strong mTYR inhibitory activity against both substrates was observed for derivatives **5** (R^1^ and R^2^ = benzothiazol-2-yl) and **9** (R^1^ = benzoyl and R^2^ = benzothiazol-2-yl) with an *S*-benzothiazol-2-yl moiety. These derivatives had IC_50_ values of 8.5 ± 1.4 and 15.6 ± 0.8 μM in the presence of l-tyrosine and 5.8 ± 1.6 and 5.7 ± 1.3 μM in the presence of l-DOPA, demonstrating that they inhibited mTYR activity 2–6 times more potently than KA.

He et al. reported that compounds in which the 2-thiobenzothiazole, 2-thiobenzoxazole, 4-methyl-3-thiotriazole, and 1-methyl-5-thiotetrazole moieties of AAMA derivatives were conjugated to kojic acid exhibited IC_50_ values of 3–20 μM against mTYR [33]. These IC_50_ values are similar to those of derivatives **5** and **9**. Moreover, according to the literature [34], compounds that substitute carbazole for the *N*-aryl moiety of AAMA derivatives have IC_50_ values that are comparable to those of derivatives **5** and **9**. These results suggest that the four *S*-aryl moieties, especially 2-thiobenzothiazole and 2-thiobenzoxazole, may play important roles in mTYR inhibitory activity.

### 2.3. Cu^2+^-Chelation Efficacy of AAMA Derivatives

To examine whether AAMA derivatives could chelate Cu^2+^ ions, the Cu^2+^-chelation efficacy was evaluated using a Cu^2+^-chelating agent, viz., pyrocatechol violet (PV) [35]. When PV chelates copper ions, the copper-chelating complex forms a bluish-violet color [36]. Therefore, the extent to which the bluish-violet color produced by PV and CuSO_4_ was attenuated by the addition of AAMA derivatives was measured to investigate their Cu^2+^-chelating efficacy.

**Figure 2:**
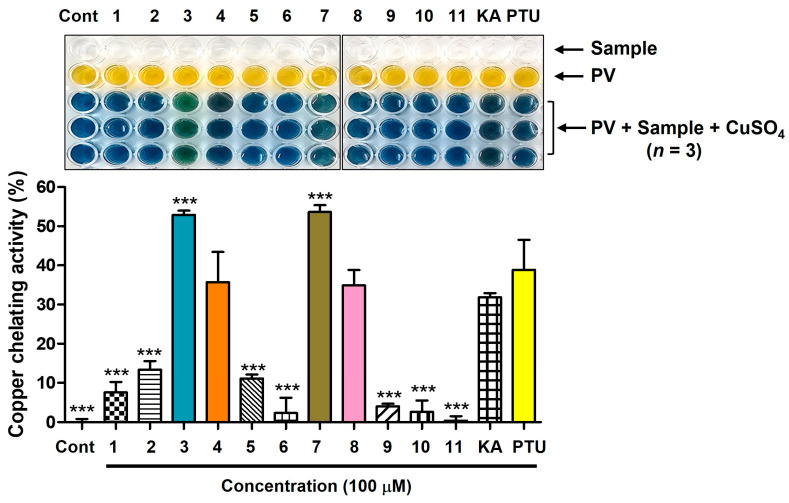
Cu^2+^-chelating potency of AAMA derivatives obtained from an experiment using pyrocatechol violet (PV; 80 μM) and CuSO_4_ (208 μM). All test samples (derivatives **1**–**11**, KA, and PTU) were tested at 100 μM. KA and PTU were employed as positive substances. *** *p* < 0.001 vs. group treated with KA. Cont = control.

Figure 2 shows the copper-ion-chelating potencies of derivatives **1**–**11**. KA and PTU, used as positive controls, exhibited copper ion-chelating activities of 32% and 39%, respectively. Derivatives **4** and **8**, which bear *S*-(1-methyltetrazol-5-yl) moieties, exhibited copper-ion-chelating activities between those of KA and PTU. The copper-chelating activities are 36% and 35%, respectively. Derivatives **3** and **7**, which contain an *S*-(4-methyl-1,2,4-tetrazol-3-yl) moiety, had 53% and 54% copper-chelating activity, respectively, providing greater potency than that of KA and PTU. The other derivatives showed low copper ion-chelating activities of less than 14%.

Considering that only AAMA derivatives **3**, **4**, **7**, and **8**, with *S*-(1-methyltetrazol-5-yl) and *S*-(4-methyl-1,2,4-tetrazol-3-yl) moieties, showed potent copper-ion-chelation activity, the chelation mode of these derivatives is mode II, as described in Figure 1. However, none of these derivatives exhibited potent mTYR inhibitory activity, suggesting that AAMA derivatives cannot act as copper chelators in mTYR inhibition. Even if a compound has copper-ion-chelating ability in an experiment using PV, there is no guarantee that the compound can chelate copper ions in a Cu^2+^-containing metalloenzyme system, such as mTYR. When a compound binds to the copper ion present in the mTYR active site, it interacts with the surrounding amino acid residues. Thus, if the interactions with amino acid residues are unfavorable, even a proven metal chelator in enzyme-free experiments may not function as a metal chelator at the active site of the metalloenzyme, including mTYR. Similar results are observed with curcumin derivatives [37] and peptides inhibiting mTYR [38]. Collectively, it is thought that derivatives **5**, **9**, and **10** exhibit mTYR inhibitory activity not via chelation with the copper ions of mTYR, but via favorable interactions with the amino acid residues present in the active site.

### 2.4. Inhibition Mechanism of AAMA Derivatives ***5***, ***9***, and ***10*** on mTYR

Derivatives **5**, **9**, and **10** displayed more potent inhibitory activities than KA against mTYR in the presence of l-DOPA. Therefore, the inhibition modes of these derivatives were investigated using kinetic experiments. Derivatives **5** and **9** were used at concentrations of 1.25, 5, and 20 μM, derivative **10** was used at concentrations of 4, 16, and 64 μM, and l-DOPA concentrations were 1, 2, 4, 8, and 16 mM. To obtain the initial dopachrome production rate, the change in optical density per minute was measured at 475 nm.

**Figure 3:**
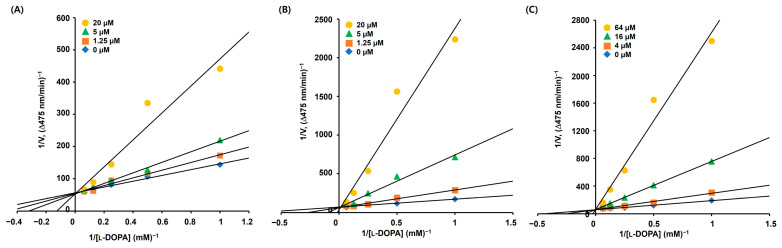
Lineweaver–Burk (L–B) plots of AAMA derivatives obtained from the kinetic experiments conducted using mTYR. (**A**–**C**) represent L–B plots of **5**, **9**, and **10**, respectively. Derivatives **5** and **9** were tested at 0, 1.25, 5, and 20 μM, and derivative **10** was tested at 0, 4, 16, and 64 μM. The substrate, l-DOPA, was used at 1, 2, 4, 8, and 16 mM.

Lineweaver–Burk (L–B) plots for each derivative (Figure 3) were drawn based on the corresponding experimental kinetic data. All L–B plots showed four straight lines, and the lines merged at a single point on the y-axis. Regardless of the concentration of the derivatives, the maximum velocity (V_max_) in each L–B plot was consistent, and the Michaelis constant (K_M_) increased with increasing concentration of the derivatives. These characteristics indicated that derivatives **5**, **9**, and **10** were competitive mTYR inhibitors that bound to the active site.

**Figure 4:**
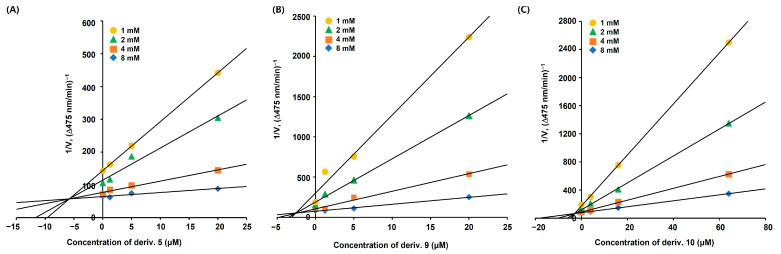
Dixon plots of AAMA derivatives **5**, **9**, and **10** converted from L–B plots. (**A**–**C**) represent Dixon plots of **5**, **9**, and **10**, respectively. The substrate l-DOPA was used at 1, 2, 4, and 8 mM. Derivatives **5** and **9** were used at 0, 1.25, 5, and 20 μM, while derivative **10** was used at 0, 4, 16, and 64 μM.

The Dixon plots for each derivative (Figure 4) were drawn using the same kinetic experimental data used to draw the L–B plots. Similarly to the L–B plots, each Dixon plot showed four straight lines; however, these lines converged at one point in the second quadrant instead of on the y-axis. The inhibition constants (K_i_) for derivatives **5**, **9**, and **10**, obtained from the merged points, were 5.8, 2.5, and 2.9 μM, respectively, implying that these derivatives have binding strengths similar to mTYR.

### 2.5. Prediction of In Silico Chemical Interactions Between AAMA Derivatives and mTYR

To predict the in silico chemical interactions between the AAMA derivatives and mTYR and their binding affinities, a docking simulation was performed. The mTYR structure for docking simulation was acquired from the Protein Data Bank (PDB), and 2Y9X (PDB ID) (*Agaricus bisporus*) was used as the docking enzyme. Chemical ligands obtained from an energy-minimization process using Chem3D Pro 12.0 were docked to the three-dimensional (3D) structure of 2Y9X. Derivatives **5**, **9**, and **10** demonstrated strong inhibitory capacity against mTYR; these derivatives were used as ligands for docking simulations along with KA, which was used as a positive control.

**Figure 5:**
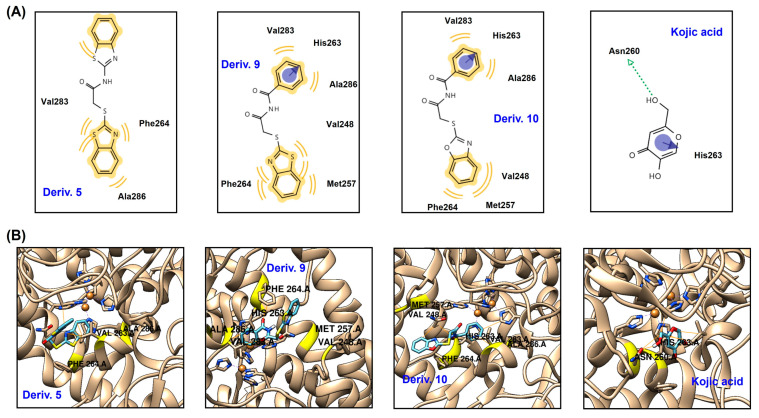
Docking simulation results between AAMA derivatives **5**, **9**, and **10** and KA and mTYR (2Y9X) using AutoDock Vina 1.2.0 software. (**A**) 2-Dimensional (2D) and (**B**) 3D pictures. The 2D and 3D pictures were acquired using LigandScout 4.4.8 software. The blue and green arrows represent π-π stacking and hydrogen bonds, respectively, and the yellow wave represents HP interactions.

All ligands were strongly bound to the mTYR active site, and the results are depicted as 3D and 2D images (Figure 5). Derivative **5** forms hydrophobic (HP) interactions with Ala286, Val283, and Phe264. Phe264 and Ala286 interact only with *S*-benzothiazole, whereas Val283 interacts hydrophobically with both benzothiazoles. Derivative **9** creates HP interactions and π-π stacking. The phenyl ring has HP interactions with Ala286 and Val283 and π-π stacking with His263, whereas the benzothiazole moiety is involved in HP interactions with Met257, Val248, and Phe264. Similarly to derivative **9**, derivative **10** produces HP interactions and π-π stacking and interacts with the same amino acids as derivative **9**. The phenyl ring interacts with His263 via π-π stacking and with Ala286 and Val283 through HP interactions, and the phenyl ring of benzoxazole interacts with Met257, Val248, and Phe264 through HP interactions. KA interacts with two amino acids present in the mTYR active site: pyranone forms π-π stacking with His263, and hydroxymethyl forms a hydrogen bond with Asn260. Three amino acids (Val283, Phe264, and Ala286) appear to play key roles in interactions with AAMA derivatives, as these three amino acids are involved in HP interactions with all derivatives. In addition, His263 also appears to play an important role in the interactions with AAMA derivatives: His263 creates π-π stacking in both derivatives **9** and **10**, as with KA. These interactions provide the ligands with strong binding affinities to mTYR, giving binding energies of −7.4, −7.7, and −7.9 kcal/mol for **5**, **9**, and **10**. The binding energies of these derivatives are much lower than those of KA (−5.4 kcal/mol), implying that **5**, **9**, and **10** bind to mTYR more tightly than KA. These docking simulation results support the kinetic study results, indicating that **5**, **9**, and **10** are competitive inhibitors. Moreover, these results are in line with the report by Havasi et al. that thymol compounds containing 2-thiobenzothiazole and 2-thiobenzoxazole moieties, such as derivatives **9** and **10**, have strong binding affinities for mTYR [39].

### 2.6. Cytotoxicity of AAMA Derivatives in B16F10 Cells

The B16F10 cell line is a murine melanoma cell line that is widely used in research on melanin production. Among the 11 derivatives, **5**, **9**, and **10**, demonstrated more potent mTYR inhibitory activities than KA, while **3**, **4**, **7**, and **8**, demonstrated copper ion-chelating activities similar to or stronger than those of KA and PTU. These results prompted us to investigate their potency in inhibiting melanin production in B16F10 cells.

Prior to the experiment on the inhibition of melanin production in B16F10 cells, the effect of AAMA derivatives on the viability of B16F10 cells was examined for 72 h. Derivatives **1**–**11** were tested at concentrations of 3.2, 8, and 20 μM.

**Figure 6:**
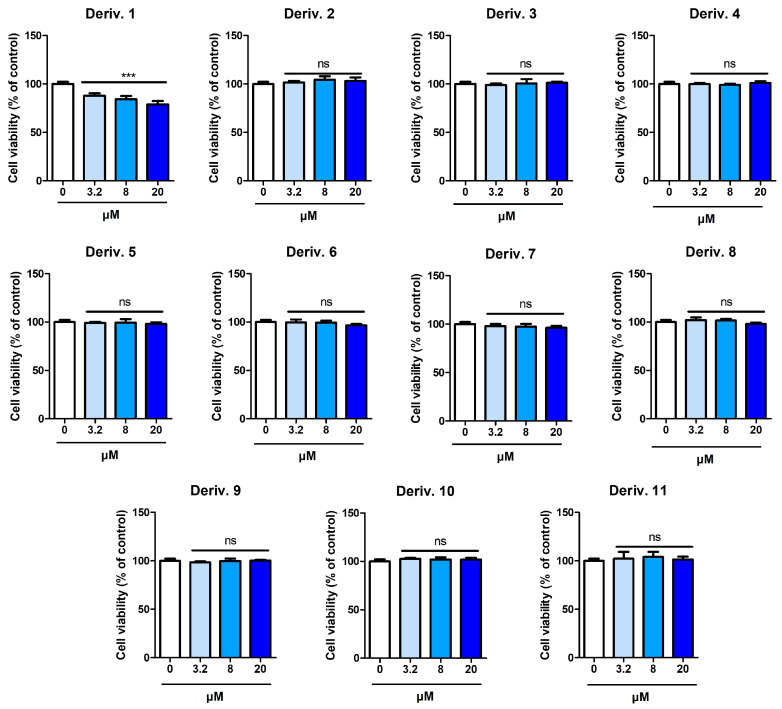
Cytotoxicity of AAMA derivatives **1**–**11** on B16F10 cells. *** *p* < 0.001 vs. the control group. ns, not significant.

Except for **1**, none of the derivatives exhibited detectable cytotoxicity at any of the tested concentrations (Figure 6). Derivative **1** exhibited weak cytotoxicity in B16F10 cells, providing cell viabilities of 91, 84, and 82% at 3.2, 8, and 20 μM, respectively. Therefore, experiments were conducted on the inhibition of melanin production in B16F10 cells at concentrations ≤ 20 μM for AAMA derivatives, excluding derivative **1**.

### 2.7. Inhibition Effect of AAMA Derivatives on Melanin Formation in B16F10 Cells

To examine the effect of AAMA derivatives on melanin formation in B16F10 cells, as a preliminary test, derivatives **2**–**11** at a concentration of 20 μM were tested for their effects on melanin levels. Derivative **1** was excluded from this test because it was cytotoxic to B16F10 cells at concentrations ≥ 3.2 μM. After treatment with 20 μM of AAMA derivatives for 1 h, B16F10 cells were treated with stimulators (α-melanocyte-stimulating hormone [α-MSH; 1 μM] and 3-isobutyl-1-methylxanthine [IBMX; 200 μM]) to boost the activity of cellular TYR (cTYR). Following a 72 h incubation period, melanin production levels were calculated by measuring the optical density at 405 nm.

Treatment with stimulators led to 2.5-fold increases in melanin content levels (Supplementary Materials S42). KA (20 μM), used as a positive control, did not significantly reduce the melanin content levels induced by stimulator treatment, but PTU (20 μM), one of the most potent TYRIs, reduced the stimulator-induced melanin content to the control levels. Among the AAMA derivatives, derivatives **2**, **5**, and **6** significantly decreased the stimulator-induced melanin content, and in particular, derivatives **5** and **6** also decreased the melanin content to almost control levels.

**Figure 7:**
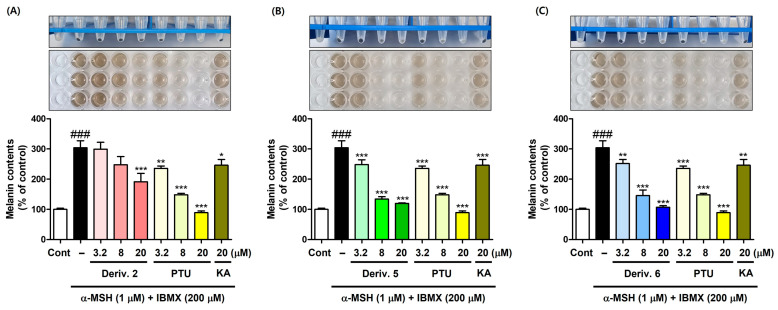
Inhibition effect of AAMA derivatives on B16F10 cellular melanin content. Derivatives **2** (**A**), **5** (**B**), and **6** (**C**) were used as test samples, and PTU and KA were used as positive reference substances. KA was tested at 20 μM, whereas **2**, **5**, **6**, and PTU were tested at three different concentrations (3.2, 8, and 20 μM). Following 1 h of sample treatment, stimulators (IBMX/α-MSH [200 μM/1 μM]) were administered to B16F10 cells for 72 h. * *p* < 0.05, ** *p* < 0.01, and *** *p* < 0.001 vs. group treated with stimulators; ### *p* < 0.001 vs. control.

Thus, derivatives **2**, **5**, and **6** at various concentrations (3.2, 8, and 20 μM) were selected for the main experiments on melanin content. Similarly to the preliminary experiment, derivatives were administered with B16F10 cells for 1 h, followed by 72 h of stimulation. The amount of melanin produced was determined by measuring the absorbance at 405 nm (Figure 7). PTU (3.2, 8, and 20 μM) and KA (20 μM) were employed as positive materials.

KA treatment significantly decreased the melanin content induced by stimulator treatment, but its effect was similar to that of PTU (3.2 μM). Derivatives **2**, **5**, and **6** significantly reduced stimulation-induced melanin content in a dose-dependent manner, their potency being in the order **5** ≈ **6** > **2**. PTU also showed a dose-dependent reduction in stimulator-induced melanin content. At 20 μM, derivatives **5** and **6** exhibited a little lower potency of melanin production inhibition than PTU, but demonstrated much more potent melanin production inhibitory potency than KA.

Similarly to the results of the mTYR activity inhibition experiment, derivatives **3**, **4**, **7**, and **8**, which demonstrated potent copper-ion-chelating activity in the PV experiment, did not show any significant inhibition of melanin content, whereas derivatives **2**, **5**, and **6**, which exhibited weak or no copper-ion-chelating capacity, showed significant and strong inhibitory activities on melanin content. These findings on B16F10 cell-based melanin production suggested that derivatives **3**, **4**, **7**, and **8** did not act as copper-chelating TYRIs in B16F10 cells, probably due to their unfavorable interactions with the cTYR amino acids, giving no inhibition in melanin production, whereas derivatives **2**, **5**, and **6** inhibited cTYR activity in B16F10 cells, probably owing to their favorable interactions with the cTYR amino acids, thus affording potent inhibition in melanin production. To determine whether the inhibition of melanin production by derivatives **2**, **5**, and **6** is due to inhibition of cTYR, the in situ cTYR inhibitory ability of these derivatives was investigated.

### 2.8. In Situ B16F10 cTYR Inhibition Potency

To identify whether the antimelanogenic effect of AAMA derivatives was driven by their cTYR inhibitory activity, the in situ cTYR inhibition potency of these derivatives was observed [40]. Test samples (**2**, **5**, and **6** and two positive materials, PTU and KA) were administered to B16F10 cells for 1 h, and then stimulators (200 μM IBMX plus 1 μM α-MSH) were applied for 72 h. To determine the in situ B16F10 cTYR inhibitory activity of **2**, **5**, and **6**, excess l-DOPA was added for 2 h, and images of the melanin-stained cells were acquired by a camera. KA was tested at 20 μM, and the other test samples were tested at concentrations of 3.2, 8, and 20 μM.

**Figure 8:**
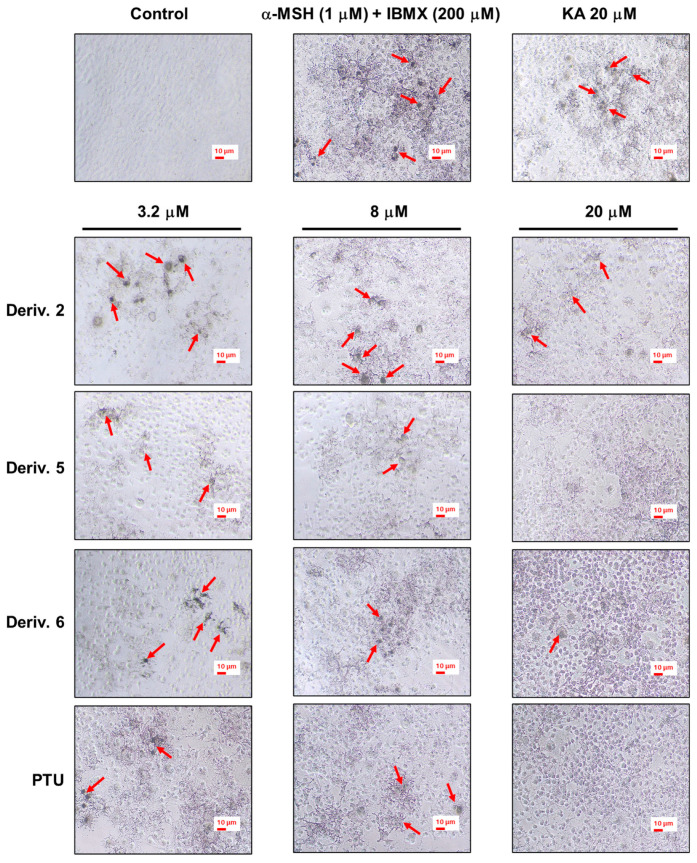
In situ B16F10 cTYR inhibition effects of derivatives **2**, **5**, and **6**. These derivatives were used as test samples, and PTU and KA were used as positive reference substances. KA was tested at 20 μM, whereas **2**, **5**, **6**, and PTU were tested at concentrations of 3.2, 8, and 20 μM. Following 1 h of sample treatment, stimulators (IBMX/α-MSH [200 μM/1 μM]) were administered to B16F10 cells for 72 h. Following l-DOPA (2 mM) treatment for 2 h, melanin-stained cells were photographed to determine in situ cTYR activity. The red arrows indicate cells that are heavily stained with melanin.

KA treatment reduced the number of melanin-stained cells compared to that of the group treated with stimulators only (Figure 8). The order of the in situ cTYR inhibition potency of the AAMA derivatives was **5** ≈ **6** > **2**, and their inhibition potency was dose-dependent. All the derivatives demonstrated stronger in situ cTYR inhibition potency than KA. In particular, the inhibitory potencies of derivatives **5** and **6** were similar to that of PTU. The results of in situ cTYR inhibition by derivatives **2**, **5**, and **6** were similar to those of melanin production, suggesting that the inhibition of melanin production by these derivatives was due to cTYR inhibition.

Considering the copper ion chelation results shown in Figure 2 and the results of melanin inhibition and cTYR activity inhibition in B16F10 cells, it is believed that the AAMA derivatives that demonstrate copper ion-chelating effects do not chelate with the copper ions present at the active site of B16F10 cTYR. The reason these derivatives do not chelate with the copper ion at the cTYR active site seems to be because of their unfavorable interactions with the amino acid residues of cTYR when the derivatives approach the copper ions within cTYR. Derivatives **2**, **5**, and **6** have strong inhibitory effects on cTYR activity and melanin production in B16F10 cells, likely because of their stronger binding affinity for the amino acid residues of cTYR than for the copper ions within cTYR.

### 2.9. Zebrafish Pigmentation Inhibition Effect

Zebrafish share 70% genetic similarity with humans, and zebrafish (*Danio rerio*) embryos (ZFE) produce most organs within 36 h post fertilization (hpf). ZFE has been used as a useful in vivo tool to identify inhibitors of melanin production [41]. To examine the depigmentation effect of AAMA derivatives on ZFL, ZFE were dechorionated at 24 hpf. After incubation in E3 embryo media (1×) for 4 h at 28 °C, test samples (KA [positive substance; 20 mM] and derivatives **1**–**11** [0.1 mM]) were applied to the dechorionated ZFE. At 72 hpf, pigment-inhibitory effects were observed in the test samples (Supplementary Materials S58–S59). Based on this observation, derivative **5** was selected for detailed depigmentation experiments at three different concentrations. The same procedure as that used in the preliminary experiments was conducted at three concentrations (0.01, 0.03, and 0.1 mM) of **5**. The depigmentation effect was compared with that of KA (20 mM). DMSO was used as the stock solution used for the derivatives. The final highest DMSO concentration in the media containing the embryos was 2%. Previous studies have shown that DMSO at concentrations ≤ 3% did not affect the pigmentation inhibitory effect of ZFL and had no detectable toxicity to them [42].

**Figure 9:**
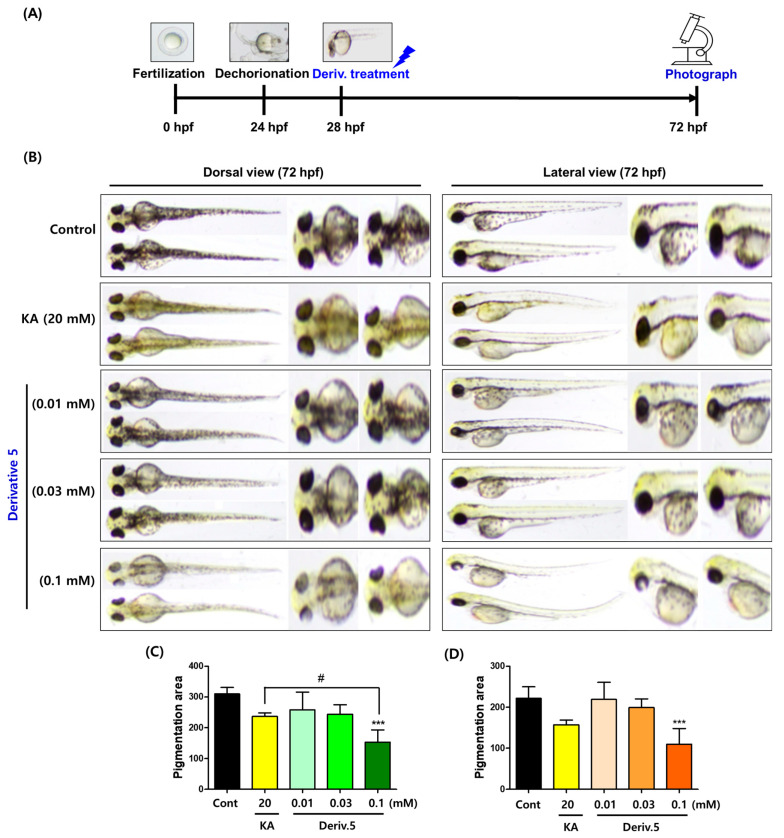
Pigment-reducing effects of AAMA derivative **5** in zebrafish larvae. (**A**) Time-based manipulation. (**B**) Zebrafish photos (dorsal and lateral views). They were taken at 30× magnification. (**C**,**D**) Pigmentation area in the dorsal and lateral views, respectively. KA (kojic acid; 20 mM) was used as a positive control. Derivative **5** was tested at three concentrations of 0.01, 0.03, and 0.1 mM. *** *p* < 0.001 vs. control; # *p* < 0.05 vs. KA.

The control group showed the darkest pigmentation throughout the body of the ZFL (Figure 9). The group exposed to KA showed lower pigmentation than the control group. Derivative **5** (0.01 mM), at a concentration 2000-fold lower than that of KA, tended to inhibit dorsal pigmentation more than in the control group, but the degree of inhibition was less than that of KA. At a concentration of 0.01 mM, **5** inhibited pigmentation more than the control group in the dorsal view. At a concentration of 0.1 mM, which is hundreds of times lower than the KA concentration, **5** inhibited pigment production in ZFL significantly better than KA in a dorsal view. This inhibition of melanin pigmentation was observed throughout the body of the ZFL, including the eyes.

There is literature reporting that compounds containing a 2-mercaptobenzothiazole moiety, such as derivative **5**, effectively inhibit pigment production in ZFL [42,43]. This suggests that the 2-mercaptobenzothiazole moiety is likely closely related to the inhibition of pigment production.

### 2.10. Cytotoxicity in Hs27 Cells

Skin whitening is one of the many applications of TYRIs. Therefore, to predict the skin application potential of AAMA derivatives, their viability was evaluated in Hs27 cells, i.e., human foreskin fibroblasts. Derivatives were treated at concentrations of 3.2, 8, and 20 μM for 24 h.

**Figure 10:**
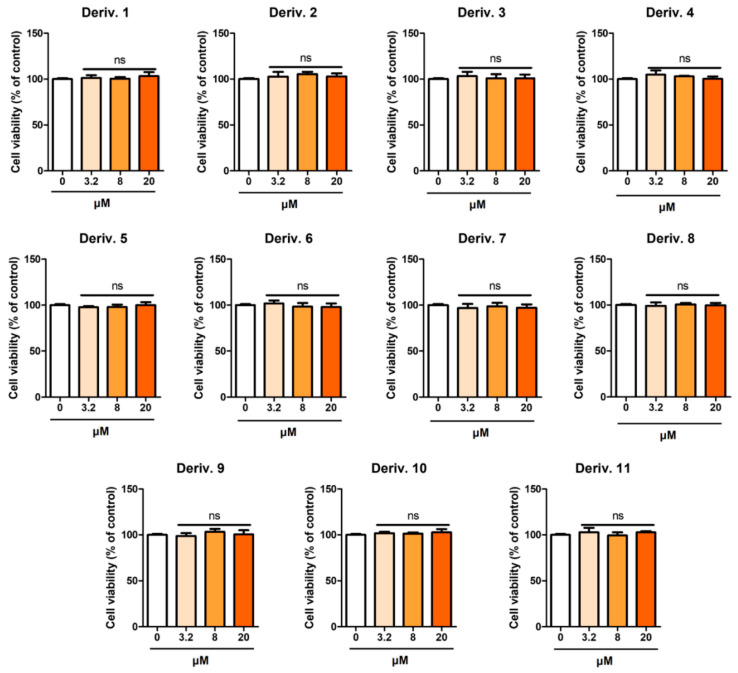
Cytotoxic effects of AAMA derivatives **1**–**11** in Hs27 cells. After AAMA derivatives (3.2, 8, and 20 μM) were administered to Hs27 for 24 h, cell viability was determined using an EZ-Cytox assay. ns, not significant.

None of the derivatives showed any discernible cytotoxicity against Hs27 cells (Figure 10). Derivative **1** also showed no cytotoxicity against Hs27 cells, in contrast to the outcomes against B16F10 cells. These results confirmed the skin application potential of AAMA derivatives, except for derivative **1**.

### 2.11. Study Limitations

There is no guarantee that compounds that inhibit mTYR and B16F10 cTYR activity will necessarily inhibit human TYR activity and exert skin-lightening effects [44]. Therefore, for AAMA derivatives to be used as pigmentation treatment drugs or cosmetics, screening for human TYR inhibition activity is essential. Furthermore, additional toxicity studies in human skin cells are likely necessary.

## 3. Materials and Methods

### 3.1. General Methods for Chemistry

^13^C and ^1^H nuclear magnetic resonance (NMR) spectroscopy was performed on a JEOL ECZ400S instrument (JEOL Ltd., Tokyo, Japan). Coupling constants are reported in hertz, and chemical shifts are recorded in ppm (*δ*). The abbreviations used in the NMR data are m (multiplet), td (triplet of doublets), s (singlet), d (doublet), and br s (broad singlet). Anhydrous solvents were obtained by distillation with CaH_2_ or Na/benzophenone. Daejung Chemicals and SEJIN CI Co. (Siheung-si and Seoul, respectively, Republic of Korea) provided the chemicals for the chemical reactions. Silica gel (MP Silica 40–63, 60 Å) was used for column chromatography. High-resolution (HR) mass spectroscopy (MS) data were collected using the ESI(+)-TOF method on a HR liquid chromatography tandem mass spectrometer (Sciex’s ZenoTOF 7600 model; SCIEX, Toronto, ON, Canada).

### 3.2. Synthesis of Compounds

#### 3.2.1. General Procedure for the Synthesis of *N*-(1*H*-Benzo[d]imidazol-2-yl)-2-chloroacetamide (**12**) and *N*-(Benzo[d]thiazol-2-yl)-2-chloroacetamide (**13**)

2-Aminobenzimidazole or 2-aminobenzothiazole was dissolved in acetone (10 mL/1 g of 2-aminobenzimidazole or 2-aminobenzothiazole), and the solution was cooled to 0 °C. 2-Chloroacetyl chloride (1.0 equiv.) in acetone (3 mL/1 mL of 2-chloroacetyl chloride) was slowly added, and the reaction mixture was refluxed for 2 h for **12** or 7 h for **13**. After evaporation of the volatiles, a NaHCO_3_ aqueous solution was added to the resulting residue to adjust the pH to 7, and the precipitates generated were filtered and washed with water to afford title compounds **12** and **13** (yields = 65% for **12** and 90% for **13**).

*N-(1H-Benzo[d]imidazol-2-yl)-2-chloroacetamide* (**12**) [29]

^1^H NMR (400 MHz, (CD_3_)_2_SO) *δ* 12.06 (s, 2H, NH×2), 7.46–7.42 (m, 2H, 4-H, 7-H), 7.13–7.09 (m, 2H, 5-H, 6-H), 4.37 (s, 2H, CH_2_Cl); ^13^C NMR (100 MHz, (CD_3_)_2_SO) *δ* 167.7, 147.5, 135.7, 122.0, 114.4, 44.0; 65%.

*N-(Benzo[d]thiazol-2-yl)-2-chloroacetamide* (**13**) [28]

^1^H NMR (400 MHz, (CD_3_)_2_SO) *δ* 12.71 (s, 1H, NH), 8.00 (d, 1H, *J* = 7.6 Hz, 4-H), 7.77 (d, 1H, *J* = 8.0 Hz, 7-H), 7.45 (td, 1H, *J* = 8.0, 1.2 Hz, 6-H), 7.33 (td, 1H, *J* = 7.6, 1.2 Hz, 5-H), 4.46 (s, 2H, CH_2_Cl); ^13^C NMR (100 MHz, (CD_3_)_2_SO) *δ* 166.4, 158.1, 149.0, 132.1, 126.8, 124.3, 122.4, 121.2, 43.1; 90%.

#### 3.2.2. Synthesis of *N*-(2-Chloroacetyl)benzamide (**14**)

2-Chloroacetyl chloride (8.2 mL, 103.01 mmol) was slowly added to benzamide (12.1 g, 99.88 mmol) at 0 °C, and the reaction mixture was refluxed for 50 min. After cooling, volatiles were evaporated under reduced pressure. Diethyl ether (30 mL) was added to the resultant residue, and the resulting precipitate was filtered and washed with diethyl ether to obtain pure *N*-(2-chloroacetyl)benzamide (**14**; 11.526 g, 58%).

^1^H NMR (400 MHz, chloroform-*d*) *δ* 9.18 (brs, 1H, NH), 7.90 (d, 2H, *J* = 7.6 Hz, 6-H, 2-H), 7.63 (t, 1H, *J* = 7.6 Hz, 4-H), 7.52 (t, 2H, *J* = 7.6 Hz, 5-H, 3-H), 4.75 (s, 2H, CH_2_Cl); ^13^C NMR (100 MHz, chloroform-*d*) *δ* 168.7, 165.5, 133.7, 131.9, 129.2, 127.9, 45.1; 58%.

#### 3.2.3. General Procedure for *N*-Aryl-*S*-aryl-2-mercaptoacetamide Derivatives **1**–**11**

An α-chloro carbonyl compound (**12**–**14**) and a mercapto compound (2-mercaptobenzothiazole, 2-mercaptobenzoxazole, 4-methyl-4*H*-1,2,4-triazole-3-thiol, or 1-methyl-1*H*-tetrazole-5-thiol) were dissolved in methanol (2 mL/50 mg of **12**–**14**), and then NaOMe (1.0 equiv.) was added to the methanol solution. The reaction mixture was stirred at 20 °C for 5–21 h, and water was added. The generated precipitates were filtered and washed with water to obtain pure AAMA derivatives **1**–**11** as solids.

*N-(1H-Benzo[d]imidazol-2-yl)-2-(benzo[d]thiazol-2-ylthio)acetamide* (**1**)

^1^H NMR (400 MHz, (CD_3_)_2_SO) *δ* 12.06 (br s, 2H, NH×2), 8.01 (m, 1H, 4′-H), 7.81 (m, 1H, 7′-H), 7.47–7.41 (m, 3H, 4-H, 7-H, 6′-H), 7.36 (td, 1H, *J* = 8.0, 1.2 Hz, 5′-H), 7.12–7.07 (m, 2H, 5-H, 6-H), 4.48 (s, 2H, CH_2_); ^13^C NMR (100 MHz, (CD_3_)_2_SO) *δ* 168.2, 166.3, 153.1, 147.3, 136.3, 135.4, 126.9, 125.1, 122.3, 121.9, 121.7, 114.4, 37.9; 76%; HRMS (ESI+) *m*/*z* C_16_H_13_N_4_OS_2_ (M+H)^+^ calcd 341.0525, obsd 341.0526.

*N-(1H-Benzo[d]imidazol-2-yl)-2-(benzo[d]oxazol-2-ylthio)acetamide* (**2**)

^1^H NMR (400 MHz, (CD_3_)_2_SO) *δ* 12.08 (br s, 2H, NH×2), 7.67–7.59 (m, 2H, 4′-H, 7′-H), 7.46–7.41 (m, 2H, 4-H, 7-H), 7.34–7.29 (m, 2H, 5′-H, 6′-H), 7.12–7.08 (m, 2H, 5-H, 6-H), 4.46 (s, 2H, CH_2_); ^13^C NMR (100 MHz, (CD_3_)_2_SO) *δ* 167.9, 164.3, 151.9, 147.2, 141.8, 136.0, 125.2, 124.9, 121.9, 118.8, 114.4, 110.8, 36.8; 69%; HRMS (ESI+) *m*/*z* C_16_H_13_N_4_O_2_S (M+H)^+^ calcd 325.0754, obsd 325.0753.

*N-(1H-Benzo[d]imidazol-2-yl)-2-((4-methyl-4H-1,2,4-triazol-3-yl)thio)acetamide* (**3**)

^1^H NMR (400 MHz, (CD_3_)_2_SO) *δ* 11.95 (br s, 2H, NH×2), 8.56 (s, 1H, N=CH), 7.44–7.41 (m, 2H, 4-H, 7-H), 7.10–7.07 (m, 2H, 5-H, 6-H), 4.14 (s, 2H, CH_2_), 3.62 (s, 3H, NCH_3_); ^13^C NMR (100 MHz, (CD_3_)_2_SO) *δ* 168.5, 149.0, 147.2, 146.8, 136.4, 121.8, 114.6, 37.9, 31.4; 58%; HRMS (ESI+) *m*/*z* C_12_H_13_N_6_OS (M+H)^+^ calcd 289.0866, obsd 289.0871.

*N-(1H-Benzo[d]imidazol-2-yl)-2-((1-methyl-1H-tetrazol-5-yl)thio)acetamide* (**4**)

^1^H NMR (400 MHz, (CD_3_)_2_SO) *δ* 12.03 (br s, 2H, NH×2), 7.45–7.41 (m, 2H, 4-H, 7-H), 7.12–7.07 (m, 2H, 5-H, 6-H), 4.36 (s, 2H, CH_2_), 4.00 (s, 3H, NCH_3_); ^13^C NMR (100 MHz, (CD_3_)_2_SO) *δ* 168.0, 153.8, 147.3, 136.0, 121.9, 114.5, 37.8, 34.3; 99%; HRMS (ESI+) *m*/*z* C_11_H_12_N_7_OS (M+H)^+^ calcd 290.0819, obsd 290.0824.

*N-(Benzo[d]thiazol-2-yl)-2-(benzo[d]thiazol-2-ylthio)acetamide* (**5**) [31]

^1^H NMR (400 MHz, CDCl_3_) *δ* 8.10 (d, 1H, *J* = 8.0 Hz, 4′-H), 7.81–7.77 (m, 3H, *J* = 8.0 Hz, 7′-H, 4-H, 7-H), 7.52 (td, 1H, *J* = 8.0, 1.2 Hz, 6′-H), 7.42–7.36 (m, 2H, 5′-H, 6-H), 7.29 (td, 1H, *J* = 8.0, 1.2 Hz, 5-H), 4.20 (s, 2H, CH_2_); ^13^C NMR (100 MHz, CDCl_3_) *δ* 167.1, 166.5, 157.5, 152.1, 148.8, 135.5, 132.5, 126.8, 126.2, 125.3, 124.0, 122.0, 121.4, 121.4, 121.4, 36.5; 74%.

*2-(Benzo[d]oxazol-2-ylthio)-N-(benzo[d]thiazol-2-yl)acetamide* (**6**) [31]

^1^H NMR (400 MHz, (CD_3_)_2_SO) *δ* 12.80 (s, 1H, NH), 7.97 (d, 1H, *J* = 8.0 Hz, 4-H), 7.78 (d, 1H, *J* = 8.0 Hz, 7-H), 7.65–7.63 (m, 1H, 4′-H), 7.62–7.60 (m, 1H, 7′-H), 7.45 (td, 1H, *J* = 8.0, 1.2 Hz, 6-H), 7.34–7.29 (m, 3H, 5-H, 5′-H, 6′-H), 4.54 (s, 2H, CH_2_); ^13^C NMR (100 MHz, (CD_3_)_2_SO) *δ* 167.0, 164.0, 158.2, 151.9, 149.1, 141.7, 132.0, 126.8, 125.3, 125.0, 124.3, 122.3, 121.3, 118.9, 110.8, 36.2: 40%.

*N-(Benzo[d]thiazol-2-yl)-2-((4-methyl-4H-1,2,4-triazol-3-yl)thio)acetamide* (**7**)

^1^H NMR (400 MHz, (CD_3_)_2_SO) *δ* 12.64 (s, 1H, NH), 8.57 (s, 1H, N=CH), 7.97 (d, 1H, *J* = 8.0 Hz, 4-H), 7.76 (d, 1H, *J* = 8.0 Hz, 7-H), 7.44 (td, 1H, *J* = 8.0, 1.2 Hz, 6-H), 7.31 (td, 1H, *J* = 8.0, 1.2 Hz, 5-H), 4.21 (s, 2H, CH_2_), 3.61 (s, 3H, CH_3_); ^13^C NMR (100 MHz, (CD_3_)_2_SO) *δ* 167.8, 158.2, 149.0, 148.9, 146.9, 132.0, 126., 124.2, 122.3, 121.2, 36.9, 31.4; 60%; HRMS (ESI+) *m*/*z* C_12_H_12_N_5_OS_2_ (M+H)^+^ calcd 306.0478, obsd 306.0479.

*N-(Benzo[d]thiazol-2-yl)-2-((1-methyl-1H-tetrazol-5-yl)thio)acetamide* (**8**)

^1^H NMR (400 MHz, (CD_3_)_2_SO) *δ* 12.72 (s, 1H, NH), 7.97 (d, 1H, *J* = 8.0 Hz, 4-H), 7.77 (d, 1H, *J* = 8.0 Hz, 7-H), 7.44 (td, 1H, *J* = 8.0, 1.2 Hz, 6-H), 7.31 (td, 1H, *J* = 8.0, 1.2 Hz, 5-H), 4.43 (s, 2H, CH_2_), 3.99 (s, 3H, CH_3_); ^13^C NMR (100 MHz, (CD_3_)_2_SO) *δ* 167.1, 158.2, 153.6, 149.0, 132.0, 126.8, 124.3, 122.3, 121.3, 37.0, 34.3; 96%; HRMS (ESI+) *m*/*z* C_11_H_11_N_6_OS_2_ (M+H)^+^ calcd 307.0430, obsd 307.0432.

*N-(2-(Benzo[d]thiazol-2-ylthio)acetyl)benzamide* (**9**)

^1^H NMR (400 MHz, CDCl_3_) *δ* 10.45 (br s, 1H, NH), 7.86 (d, 2H, *J* = 7.2 Hz, 2-H, 6-H), 7.82–7.78 (m, 2H, *J* = 8.0 Hz, 4′-H, 7′-H), 7.56 (t, 1H, *J* = 7.2 Hz, 4-H), 7.45 (td, 1H, *J* = 8.0, 1.2 Hz, 6′-H), 7.40 (t, 2H, *J* = 7.2 Hz, 3-H, 5-H), 7.36 (td, 1H, *J* = 8.0, 1.2 Hz, 5′-H), 4.37 (s, 2H, CH_2_); ^13^C NMR (100 MHz, CDCl_3_) *δ* 168.2, 166.9, 165.5, 152.2, 135.6, 133.3, 132.9, 129.0, 128.0, 126.5, 125.1, 121.5, 121.5, 38.3; 29%; HRMS (ESI+) *m*/*z* C_16_H_13_N_2_O_2_S_2_ (M+H)^+^ calcd 329.0413, obsd 329.0412.

*N-(2-(Benzo[d]oxazol-2-ylthio)acetyl)benzamide* (**10**)

^1^H NMR (400 MHz, CDCl_3_) *δ* 10.17 (br s, 1H, NH), 7.92 (d, 2H, *J* = 7.2 Hz, 2-H, 6-H), 7.60–7.58 (m, 2H, 4-H, 4′-H), 7.49–7.45 (m, 3H, 3-H, 5-H, 7′-H), 7.33 (td, 1H, J = 7.6, 1.6 Hz, 6′-H), 7.29 (td, 1H, J = 7.6, 1.6 Hz, 5′-H), 4.45 (s, 2H, CH_2_); ^13^C NMR (100 MHz, CDCl_3_) *δ* 168.3, 165.5, 164.8, 152.3, 141.2, 133.5, 132.5, 129.0, 128.0, 124.8, 124.6, 118.4, 110.4, 37.6; 49%; HRMS (ESI+) *m*/*z* C_16_H_13_N_2_O_3_S (M+H)^+^ calcd 313.0641, obsd 313.0643.

*N-(2-((1-Methyl-1H-tetrazol-5-yl)thio)acetyl)benzamide* (**11**)

^1^H NMR (400 MHz, (CD_3_)_2_SO) *δ* 11.50 (s, 1H, NH), 7.96 (d, 2H, *J* = 8.0 Hz, 2-H, 6-H), 7.66 (t, 1H, *J* = 8.0 Hz, 4-H), 7.54 (t, 2H, *J* = 8.0 Hz, 3-H, 5-H), 4.70 (s, 2H, CH_2_), 3.99 (s, 3H, CH_3_); ^13^C NMR (100 MHz, (CD_3_)_2_SO) *δ* 169.6, 167.1, 153.9, 133.6, 133.1, 129.1, 129.0, 34.2: 79%; HRMS (ESI+) *m*/*z* C_11_H_12_N_5_O_2_S (M+H)^+^ calcd 278.0706, obsd 278.0708.

### 3.3. Inhibition Assay Against mTYR [45,46]

Experiments to determine the IC_50_ values of AAMA derivatives against mTYR were performed in 96-well plates. Derivatives were tested at three different sets of concentrations (2, 10, and 50 μM, 5, 25, and 125 μM, or 1, 5, 25 μM). A substrate (170 μL) solution consisting of 17.2 mM phosphate buffer (pH 6.5) and 345 μM l-DOPA or l-tyrosine, test samples (10 μL; KA, positive substance, and **1**–**11**) dissolved in DMSO, and 500 units/mL mTYR aqueous solution (20 μL) were added sequentially to each well and then incubated for 30 min. The absorbance of each well was recorded using a VersaMax^TM^ microplate (VMM) reader (Molecular Devices, San Jose, CA, USA). Three independent experiments were conducted. The control group received the vehicle (5% DMSO) only.

### 3.4. Copper(II) Chelation Assay [35,36]

Experiments to calculate the copper-ion-chelating activity of AAMA derivatives were carried out in 96-well plates. Derivatives were tested at a concentration of 100 μM. First, 6 μL of PV (4 mM), 10 μL of test samples (positive substances [KA and PTU] and **1**–**11**), 280 μL of acetic acid-sodium acetate buffer (50 mM; pH 6.0), and 10 μL of aqueous copper sulfate solution (1 g/L) were added sequentially to each well. After maintaining the temperature at 19 °C for 20 min, the absorbance of each well at 632 nm was recorded using a VMM reader. The percentage copper chelation activity was acquired using the following equation: 100 × (1 − ABS_sample_/ABS_control_), where ABS_sample_ and ABS_control_ are the optical densities of test samples and control, respectively. The control group received the vehicle (3.3% DMSO) only.

### 3.5. Kinetic Study Using mTYR

Kinetic experiments were conducted to identify the inhibitory mechanisms of derivatives **5**, **9**, and **10** on mTYR. The initial dopachrome production rates were obtained in the presence of l-DOPA and the AAMA derivative at different concentrations in a 96-well plate. To a mixture of l-DOPA substrate solution (170 μL) comprising l-DOPA (final conc.: 16, 8, 4, 2, and 1 mM) and sodium phosphate buffer (pH 6.5; final conc.: 14.7 mM) and test sample (**5**, **9**, and **10**; 10 μL) in each well, mTYR solution (120 units/mL; 20 μL) was added. The final concentrations were 0, 1.25, 5, and 20 μM for **5** and **9** and 0, 4, 16, and 64 μM for **10**. To prepare the L–B and Dixon plots, ΔOD_475_/min (the change in well optical density at 475 nm/min) was calculated by measuring the absorbance at 475 nm at 1 min intervals for 20 min while incubating at 37 °C. The control group (derivative: 0 μM) received the vehicle (5% DMSO) only.

### 3.6. Docking Simulation with mTYR

For the docking simulation of AAMA derivatives, the mTYR X-ray crystal structure was obtained from the PDB ID: 2Y9X (*Agaricus bisporus*) [47]. The energetically minimized 3D ligand (**5**, **9**, **10**, and KA) structures were prepared using Chem3D Pro 12.0 (https://cs-chemdraw-pro.software.informer.com, accessed on 16 September 2025). Using AutoDock Vina 1.2.0 (The Scripps Research Institute, La Jolla, San Diego, CA, USA), docking simulation was performed by docking the 3D ligand structures to 2Y9X, in which tropolone, the original ligand of 2Y9X, was removed. To identify possible interactions between the ligands and the prepared mTYR structure, LigandScout 4.4.8 (http://www.inteligand.com/ligandscout/download.shtml, accessed on 22 September 2025) (Inte:Ligand GmbH, Vienna, Austria) was utilized. To validate the structure of mTYR prepared for docking, redocking with tropolone was carried out [48,49]. The difference between the experimental and co-crystallized poses provided a root-mean-square deviation of 0.99 Å (Supplementary Materials S62), suggesting that the prepared mTYR structure could be used to correctly dock AAMA derivatives.

### 3.7. Cell Culture

For the cultivation of Hs27 and B16F10 cells supplied by the Korean Cell Line Bank (Seoul, Republic of Korea), Dulbecco’s modified Eagle’s medium with fetal bovine serum (10%) and penicillin-streptomycin solution (100 units/mL) were used. The cells were incubated at 5% CO_2_ and 37 °C in a humidified environment. All the reagents used for cell culture were supplied by Thermo Fisher Scientific (Waltham, MA, USA).

### 3.8. Cell Toxicity Assay in B16F10 Cells [50]

A 96-well plate with 800 B16F10 cells/well was placed in an incubator set at 37 °C for 24 h. A DMSO solution of derivatives **1**–**11** (0, 3.2, 8, and 20 μM) was added to each well, and the plate was placed for 72 h in an incubator. Cells were cultivated for 2 h after an EZ-Cytox solution treatment (10 μL/well; EZ-1000, DoGenBio, Seoul, Republic of Korea) to each well. Cell viability results were obtained by measuring the ABS_λ450_ using a VMM reader. Percentage cell viability = (ABS_sample_/ABS_control_) × 100, where ABS_sample_ and ABS_control_ are the optical densities of test samples and control, respectively. The control group received the vehicle (0.1% DMSO) only.

### 3.9. Melanin Content Level Assay in B16F10 Cells [51,52]

A six-well plate with 4000 B16F10 cells/well was placed in an incubator set at 37 °C and 5% CO_2_ for 24 h. The cultivated cells were exposed to derivatives **1**–**11** (20 μM) for the preliminary experiment and derivatives **2**, **5**, and **6** (3.2, 8, and 20 μM) for the main experiment. KA and PTU, used as positive substances, were tested at 20 μM for the preliminary experiment and at 20 μM and 3.2, 8, and 20 μM, respectively, for the main experiment. After 1 h, stimulators consisting of IBMX/α-MSH (200 μM/1 μM) were administered to B16F10 cells and incubated for 72 h. Using Dulbecco’s phosphate-buffered saline (DPBS), the cultivated cells were washed, exposed to 1 N NaOH (100 μL), and cultivated at 60 °C for 1 h. The ABS_λ405_ of each well was recorded using a VMM reader. All the results were normalized to the total protein concentration of the cell lysates using bicinchoninic acid (BCA) assay reagent (Pierce, Thermo Fisher Scientific, MA, USA). Three independent experiments were conducted. The control group received the vehicle (0.1% DMSO) only.

### 3.10. In Situ cTYR Activity Assay in B16F10 Cells [53,54]

A 24-well plate with 3000 B16F10 cells/well was placed in an incubator set at 37 °C and 5% CO_2_ for 24 h. The cultivated cells were exposed to derivatives **2**, **5**, and **6** (3.2, 8, and 20 μM) or positive controls (PTU [3.2, 8, and 20 μM] or KA [20 μM]) and after 1 h, treated with stimulators (IBMX/α-MSH [200 μM/1 μM]) for 72 h. The cells were fixed, washed, and permeabilized for 2 min using a 4% paraformaldehyde aqueous solution, DPBS, and 0.1% Triton X-100. l-DOPA solution (500 μL; 2 mM) was dispensed to each well. After 2 h, images of the melanin-stained cells were captured using a camera attached to a Motic stereomicroscope (Hong Kong, China). The control group received the vehicle (0.1% DMSO) only.

### 3.11. In Vivo Pigmentation Assay Using Zebrafish Embryos (ZFE) [55,56]

For the in vivo zebrafish (*Danio rerio*) pigmentation assay with AAMA derivatives, ZFE in an E3-MB media at 28 °C were dechorionated using Pronase^®^ (Sigma-Aldrich, St. Louis, MO, USA) at 24 h post fertilization (hpf). The E3-MB media were prepared by mixing calcium chloride, sodium chloride, potassium chloride, and magnesium sulfate in concentrations of 0.33, 5, 0.17, and 0.33 mM, respectively, and methylene blue solution was added to make a 0.001% solution. In a 48-well plate, derivatives **1**–**11** (0.1 mM in DMSO) were applied to six ZFE dispensed in each well containing 500 μL of E3 media at 28 hpf. At 72 hpf, pigmentation was determined by capturing images of ZFL using a camera connected to an SMZ745T Nikon stereomicroscope (Tokyo, Japan). On the basis of this preliminary experiment (one replicate), derivative **5** was selected as the test sample for the main experiment. The main experiment was conducted in the same manner as the preliminary experiment. However, derivative **5** was tested at three concentrations (0.01, 0.03, and 0.1 mM), and the experiments were repeated three times (three replicates). The degree of pigmentation of the ZFL was calculated using ATTO CS analysis software version 3.2 (Tokyo, Japan), which converts the concentration of pigment into a numerical value by measuring the density of the fixed area selected for measurement. KA was used as a positive standard to compare the depigmentation effects of the AAMA derivatives. The Office of Laboratory Animal Welfare (OLAW) of the National Institutes of Health considers fish species to be “live vertebrate animals” at “hatching”. OLAW considers zebrafish hatching to occur at 72 h/3 days post fertilization (dpf). Therefore, zebrafish 0–3 dpf are not considered live vertebrate animals, and no ethical approval was required for experiments on ZFL. The control group received the vehicle (2% DMSO) only.

### 3.12. Cytotoxicity in Hs27 Cells [57]

A 96-well plate including 10,000 Hs27 cells per well was incubated at 37 °C and 5% CO_2_ for 24 h. AAMA derivatives at various concentrations (0, 3.2, 8, and 20 μM) were supplied to each well, and the plate was incubated at 37 °C and 5% CO_2_ for 24 h. After treatment with 10 μL EZ-Cytox solution for 2 h, the well optical density was measured at 450 nm using a VMM reader to determine cytotoxicity. Three independent experiments were conducted. The control group received the vehicle (0.1% DMSO) only.

### 3.13. Statistical Analysis

Each experiment was performed in triplicate. All data are presented as the mean ± standard deviation. Data were analyzed by one-way analysis of variance with the Bonferroni test using GraphPad Prism (version 5.0; Boston, MA, USA). Statistical significance (*p*) < 0.05 is acceptable.

## 4. Conclusions

In this study, 11 *N*-aryl-*S*-aryl-2-mercaptoacetamide (AAMA) derivatives were designed and synthesized as tyrosinase inhibitors. Derivatives **3**, **4**, **7**, and **8** showed stronger copper-ion-chelating activities than KA and PTU in experiments with pyrocatechol violet. In contrast, these four derivatives did not inhibit mTYR or melanin production in B16F10 cells. Derivatives that did not show a copper-ion-chelation effect showed mTYR inhibitory activity, cTYR inhibitory activity, and melanin production inhibitory activity in B16F10 cells or a depigmentation effect in ZFL. These results indicated that the results of copper-ion-chelation experiments performed in an enzyme-free environment may not necessarily be consistent with the results of copper-ion chelation performed in a copper-containing metalloenzyme. Derivatives **2**, **5**, **6**, and **9**, which exhibited little copper-ion-chelating activity, inhibited mTYR activity, cTYR activity, melanogenesis in B16F10 cells, or pigment production in ZFL. In particular, derivative **5** showed broad activity, inhibiting mTYR activity, cTYR activity, melanogenesis in B16F10 cells, and pigment production in ZFL. Docking simulations and kinetic studies revealed that derivative **5** competitively inhibited mTYR by interacting with amino acid residues present in the active site of mTYR.

## Figures and Tables

**Table 1:** Chemical structures of AAMA derivatives and their IC_50_ values against mTYR.

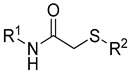				
Deriv.	R^1^	R^2^	IC_50_ Value (μM) ^a^	
			l-Tyrosine	l-DOPA
**1**	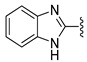	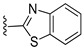	>125	>125
**2**	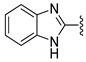	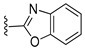	>125	>125
**3**	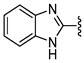	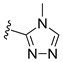	>125	>125
**4**	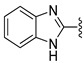	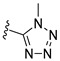	>125	>125
**5**	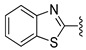	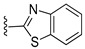	8.5 ± 1.4	5.8 ± 1.6
**6**	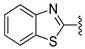	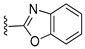	>125	>125
**7**	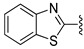	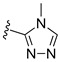	>125	>125
**8**	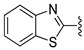	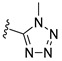	>125	>125
**9**	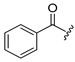	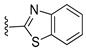	15.6 ± 0.8	5.7 ± 1.3
**10**	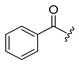	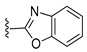	>125	16.9 ± 0.7
**11**	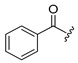	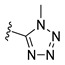	>125	>125
KA ^b^	-	-	31 ± 2	36.9 ± 1.5

^a^ Each IC_50_ is expressed as the mean ± SD of experiments performed independently in triplicate. ^b^ KA (kojic acid; positive control) was used for comparing inhibitory activity.

## Data Availability

The data presented in this study are available in the article and Supplementary Materials.

## Supplementary Materials

The following supporting information can be downloaded at: https://www.mdpi.com/article/10.3390/molecules31030422/s1, S1. ^1^H NMR spectrum of derivative **1**; S2. ^13^C NMR spectrum of derivative **1**; S3. HRMS spectrum of derivative **1**; S4. ^1^H NMR spectrum of derivative **2**; S5. ^13^C NMR spectrum of derivative **2**; S6. HRMS spectrum of derivative **2**; S7. ^1^H NMR spectrum of derivative **3**; S8. ^13^C NMR spectrum of derivative **3**; S9. HRMS spectrum of derivative **3**; S10. ^1^H NMR spectrum of derivative **4**; S11. ^13^C NMR spectrum of derivative **4**; S12. HRMS spectrum of derivative **4**; S13. ^1^H NMR spectrum of derivative **5**; S14. ^13^C NMR spectrum of derivative **5**; S15. ^1^H NMR spectrum of derivative **6**; S16. ^13^C NMR spectrum of derivative **6**; S17. ^1^H NMR spectrum of derivative **7**; S18. ^13^C NMR spectrum of derivative **7**; S19. HRMS spectrum of derivative **7**; S20. ^1^H NMR spectrum of derivative **8**; S21. ^13^C NMR spectrum of derivative **8**; S22. HRMS spectrum of derivative **8**; S23. ^1^H NMR spectrum of derivative **9**; S24. ^13^C NMR spectrum of derivative **9**; S25. HRMS spectrum of derivative **9**; S26. ^1^H NMR spectrum of derivative **10**; S27. ^13^C NMR spectrum of derivative **10**; S28. HRMS spectrum of derivative **10**; S29. ^1^H NMR spectrum of derivative **11**; S30. ^13^C NMR spectrum of derivative **11**; S31. HRMS spectrum of derivative **11**; S32. ^1^H NMR spectrum of derivative **12**; S33. ^13^C NMR spectrum of derivative **12**; S34. ^1^H NMR spectrum of derivative **13**; S35. ^13^C NMR spectrum of derivative **13**; S36. ^1^H NMR spectrum of derivative **14**; S37. ^13^C NMR spectrum of derivative **14**; S38. Graphs used to calculate the IC_50_ values for derivatives **5** and **9** in the presence of l-tyrosine; S39. Graphs used to calculate the IC_50_ value for kojic acid in the presence of l-tyrosine; S40. Graphs used to calculate the IC_50_ values for derivatives **5** and **9** in the presence of l-DOPA; S41. Graphs used to calculate the IC_50_ values for derivative **10** and kojic acid in the presence of l-DOPA; S42. Melanin content levels in the presence of AAMA derivatives **2**–**11** in B16F10 cells; S43. Images of the control group (*n* = 6) in the in situ B16F10 cellular tyrosinase activity experiments; S44. Images of the α-MSH + IBMX group (*n* = 9) in the in situ B16F10 cellular tyrosinase activity experiments; S45. Images of the kojic acid (20 μM) group (*n* = 7) in the in situ B16F10 cellular tyrosinase activity experiments; S46. Images of the derivative **2** (3.2 μM) group (*n* = 8) in the in situ B16F10 cellular tyrosinase activity experiments; S47. Images of the derivative **2** (8 μM) group (*n* = 8) in the in situ B16F10 cellular tyrosinase activity experiments; S48. Images of the the derivative **2** (20 μM) group (*n* = 9) in the in situ B16F10 cellular tyrosinase activity experiments; S49. Images of the derivative **5** (3.2 μM) group (*n* = 6) in the in situ B16F10 cellular tyrosinase activity experiments; S50. Images of the derivative **5** (8 μM) group (*n* = 7) in the in situ B16F10 cellular tyrosinase activity experiments; S51. Images of the the derivative **5** (20 μM) group (*n* = 7) in the in situ B16F10 cellular tyrosinase activity experiments; S52. Images of the derivative **6** (3.2 μM) group (*n* = 9) in the in situ B16F10 cellular tyrosinase activity experiments; S53. Images of the derivative **6** (8 μM) group (*n* = 10) in the in situ B16F10 cellular tyrosinase activity experiments; S54. Images of the the derivative **6** (20 μM) group (*n* = 9) in the in situ B16F10 cellular tyrosinase activity experiments; S55. Images of the PTU (3.2 μM) group (*n* = 8) in the in situ B16F10 cellular tyrosinase activity experiments; S56. Images of the PTU (8 μM) group (*n* = 8) in the in situ B16F10 cellular tyrosinase activity experiments; S57. Images of the the PTU (20 μM) group (*n* = 9) in the in situ B16F10 cellular tyrosinase activity experiments; S58. Pigment-reducing effects of derivatives **1**–**3** and kojic acid in zebrafish larvae; S59. Pigment-reducing effects of derivatives **4**–**11** in zebrafish larvae; S60 Pigment-reducing effect of kojic acid in zebrafish larvae; S61 Pigment-reducing effect of derivative **5** in zebrafish larvae; S62. Alignment of the redocked ligand (yellow; tropolone) and co-crystallized ligand (red; tropolone) with the 2Y9X protein; S63. Statistical analysis of Cu^2+^-chelation efficacy in AAMA derivatives 1–11, KA, and PTU; S64. Statistical analysis of the cytotoxicity of derivative **1** in B16F10 cells; S65. Statistical analysis of the cytotoxicity of derivative **2** in B16F10 cells; S66. Statistical analysis of the cytotoxicity of derivative **3** in B16F10 cells; S67. Statistical analysis of the cytotoxicity of derivative **4** in B16F10 cells; S68. Statistical analysis of the cytotoxicity of derivative **5** in B16F10 cells; S69. Statistical analysis of the cytotoxicity of derivative **6** in B16F10 cells; S70. Statistical analysis of the cytotoxicity of derivative **7** in B16F10 cells; S71. Statistical analysis of the cytotoxicity of derivative **8** in B16F10 cells; S72. Statistical analysis of the cytotoxicity of derivative **9** in B16F10 cells; S73. Statistical analysis of the cytotoxicity of derivative **10** in B16F10 cells; S74. Statistical analysis of the cytotoxicity of derivative **11** in B16F10 cells; S75. Statistical analysis of the inhibitory effect of derivatives **2**–**11**, KA, and PTU on melanin formation in B16F10 cells; S76. Statistical analysis of the inhibitory effect of derivative **2**, PTU, and KA on melanin formation in B16F10 cells; S77. Statistical analysis of the inhibitory effect of derivative **5**, PTU, and KA on melanin formation in B16F10 cells; S78. Statistical analysis of the inhibitory effect of derivative **6,** PTU, and KA on melanin formation in B16F10 cells; S79. Statistical analysis of the pigmentation inhibition effect of derivative **5** and KA on the zebrafish dorsal view; S80. Statistical analysis of the pigmentation inhibition effect of derivative **5** and KA on the zebrafish lateral view; S81. Statistical analysis of the cytotoxicity of derivative **1** in Hs27 cells; S82. Statistical analysis of the cytotoxicity of derivative **2** in Hs27 cells; S83. Statistical analysis of the cytotoxicity of derivative **3** in Hs27 cells; S84. Statistical analysis of the cytotoxicity of derivative **4** in Hs27 cells; S85. Statistical analysis of the cytotoxicity of derivative **5** in Hs27 cells; S86. Statistical analysis of the cytotoxicity of derivative **6** in Hs27 cells; S87. Statistical analysis of the cytotoxicity of derivative **7** in Hs27 cells; S88. Statistical analysis of the cytotoxicity of derivative **8** in Hs27 cells; S89. Statistical analysis of the cytotoxicity of derivative **9** in Hs27 cells; S90. Statistical analysis of the cytotoxicity of derivative **10** in Hs27 cells; S91. Statistical analysis of the cytotoxicity of derivative **11** in Hs27 cells.
