# Supplementary material for: N-Aryl-S-aryl-2-mercaptoacetamide Derivatives Effectively Inhibit Mushroom and Cellular Tyrosinase Activities, Melanin Production, and Pigmentation in Zebrafish Larvae: Regarding Copper Ion Chelation
Source: Molecules. 2026 Jan 26;31(3):422. doi: 10.3390/molecules31030422 (PMC12899738; doi:10.3390/molecules31030422)
Supplement: Supplementary file 1 [file molecules-31-00422-s001.zip › molecules-4088467-supplementary.pdf]

Supporting Information

For

N-Aryl-*S*-aryl-2-mercaptoacetamide Derivatives Effectively Inhibit Mushroom and Cellular Tyrosinase Activities, Melanin Production, and Pigmentation in Zebrafish Larvae: Regarding Copper Ion Chelation

Hee Jin Jung [1,2,†], Hye Jin Kang [1,2,†], Hyeon Seo Park [1,2], Minchang Kim [1,2], Hyunju Lee [1,2], Hyunhee Ju [1,2], Yeonsoo Jeong [1,2], Yujin Park [3], Hae Young Chung [4] and Hyung Ryong Moon [1,2,*]

[1] Department of Manufacturing Pharmacy, College of Pharmacy, Pusan National University, Busan 46241, Republic of Korea; hjjung2046@pusan.ac.kr (H.J.J.); dirgowls22@pusan.ac.kr (H.J.K.); gustj6956@pusan.ac.kr (H.S.P.); pawky0106@pusan.ac.kr (M.K.); lhj6384770@pusan.ac.kr (H.L.); hyunh@pusan.ac.kr (H.J.); jysoo627@pusan.ac.kr (Y.J.)

[2] Research Institute for Drug Development, Pusan National University, Busan 46241, Republic of Korea

[3] Department of Medicinal Chemistry, New Drug Development Center, Daegu-Gyeongbuk Medical Innovation Foundation, Daegu 41061, Republic of Korea; pyj1016@kmedihub.re.kr

[4] Department of Pharmacy, College of Pharmacy, Pusan National University, Busan 46241, Republic of Korea; hyjung@pusan.ac.kr

* Correspondence: mhr108@pusan.ac.kr; Tel.: +82-51-510-2815; Fax: +82-51-513-6754

† These authors contributed equally to this work.

S1. ^{1}H NMR spectrum of derivative **1** 7

S2. ^{13}C NMR spectrum of derivative **1** 8

S3. HRMS spectrum of derivative **1** 9

S4. ^{1}H NMR spectrum of derivative **2** 10

S5. ^{13}C NMR spectrum of derivative **2** 11

S6. HRMS spectrum of derivative **2** 12

S7. ^{1}H NMR spectrum of derivative **3** 13

S8. ^{13}C NMR spectrum of derivative **3** 14

S9. HRMS spectrum of derivative **3** 15

S10. ^{1}H NMR spectrum of derivative **4** 16

S11. ^{13}C NMR spectrum of derivative **4** 17

S12. HRMS spectrum of derivative **4** 18

S13. ^{1}H NMR spectrum of derivative **5** 19

S14. ^{13}C NMR spectrum of derivative **5** 20

S15. ^{1}H NMR spectrum of derivative **6** 21

S16. ^{13}C NMR spectrum of derivative **6** 22

S17. ^{1}H NMR spectrum of derivative **7** 23

S18. ^{13}C NMR spectrum of derivative **7** 24

S19. HRMS spectrum of derivative **7** 25

S20. ^{1}H NMR spectrum of derivative **8** 26

S21. ^{13}C NMR spectrum of derivative **8** 27

S22. HRMS spectrum of derivative **8** 28

S23. ^{1}H NMR spectrum of derivative **9** 29

S24. ^{13}C NMR spectrum of derivative **9** 30

S25. HRMS spectrum of derivative **9** 31

S26. ^{1}H NMR spectrum of derivative **10** 32

S27. ^{13}C NMR spectrum of derivative **10** 33

S28. HRMS spectrum of derivative **10** 34

S29. ^{1}H NMR spectrum of derivative **11** 35

S30. ^{13}C NMR spectrum of derivative **11** 36

S31. HRMS spectrum of derivative **11** 37

S32. ^{1}H NMR spectrum of derivative **12** 38

S33. ^{13}C NMR spectrum of derivative **12** 39

S34. ^{1}H NMR spectrum of derivative **13** 40

S35. ^{13}C NMR spectrum of derivative **13** 41

S36. ^{1}H NMR spectrum of derivative **14** 42

S37. ^{13}C NMR spectrum of derivative **14** 43

S38. Graphs used to calculate the IC_{50} values for derivatives **5** and **9** in the presence of L-tyrosine 44

S39. Graphs used to calculate the IC_{50} value for kojic acid in the presence of L-tyrosine 45

S40. Graphs used to calculate the IC_{50} values for derivatives **5** and **9** in the presence of L-dopa 46

S41. Graphs used to calculate the IC_{50} values for derivative **10** and kojic acid in the presence of L-dopa 47

S42. Melanin content levels in the presence of AAMA derivatives **2–11** in B16F10 cells.......... 48

S43. Images of the control group (*n* = 6) in the in situ B16F10 cellular tyrosinase activity experiments 49

S44. Images of the α-MSH + IBMX group (*n* = 9) in the in situ B16F10 cellular tyrosinase activity experiments 50

S45. Images of the kojic acid (20 μM) group (*n* = 7) in the in situ B16F10 cellular tyrosinase activity experiments 51

S46. Images of the derivative **2** (3.2 μM) group (*n* = 8) in the in situ B16F10 cellular tyrosinase activity experiments 52

S47. Images of the derivative **2** (8 μM) group (*n* = 8) in the in situ B16F10 cellular tyrosinase activity experiments 53

S48. Images of the the derivative **2** (20 μM) group (*n* = 9) in the in situ B16F10 cellular tyrosinase activity experiments 54

S49. Images of the derivative **5** (3.2 μM) group (*n* = 6) in the in situ B16F10 cellular tyrosinase activity experiments 55

S50. Images of the derivative **5** (8 μM) group (*n* = 7) in the in situ B16F10 cellular tyrosinase activity experiments 56

S51. Images of the the derivative **5** (20 μM) group (*n* = 7) in the in situ B16F10 cellular tyrosinase activity experiments 57

S52. Images of the derivative **6** (3.2 μM) group (*n* = 9) in the in situ B16F10 cellular tyrosinase activity experiments 58

S53. Images of the derivative **6** (8 μM) group (*n* = 10) in the in situ B16F10 cellular tyrosinase activity experiments 59

S54. Images of the the derivative **6** (20 μM) group (*n* = 9) in the in situ B16F10 cellular tyrosinase activity experiments 60

S55. Images of the PTU (3.2 μM) group (*n* = 8) in the in situ B16F10 cellular tyrosinase activity experiments 61

S56. Images of the PTU (8 μM) group (n = 8) in the in situ B16F10 cellular tyrosinase activity experiments 62
S57. Images of the the PTU (20 μM) group (n = 9) in the in situ B16F10 cellular tyrosinase activity experiments 63
S58. Pigment-reducing effects of derivatives **1**–**3** and kojic acid in zebrafish larvae 64
S59. Pigment-reducing effects of derivatives **4**–**11** in zebrafish larvae 65
S60. Pigment-reducing effect of kojic acid in zebrafish larvae ………...……….........………66
S61. Pigment-reducing effect of derivative **5** in zebrafish larvae …….......…………………67
S62. Alignment of the redocked ligand (yellow; tropolone) and co-crystallized ligand (red: tropolone) with the 2Y9X protein 68
S63. Statistical analysis of Cu^{2+}-chelation efficacy in AAMA derivatives **1**−**11**, KA, and PTU 69
S64. Statistical analysis of the cytotoxicity of derivative **1** in B16F10 cells 72
S65. Statistical analysis of the cytotoxicity of derivative **2** in B16F10 cells 73
S66. Statistical analysis of the cytotoxicity of derivative **3** in B16F10 cells 74
S67. Statistical analysis of the cytotoxicity of derivative **4** in B16F10 cells 75
S68. Statistical analysis of the cytotoxicity of derivative **5** in B16F10 cells 76
S69. Statistical analysis of the cytotoxicity of derivative **6** in B16F10 cells 77
S70. Statistical analysis of the cytotoxicity of derivative **7** in B16F10 cells 78
S71. Statistical analysis of the cytotoxicity of derivative **8** in B16F10 cells 79
S72. Statistical analysis of the cytotoxicity of derivative **9** in B16F10 cells 80
S73. Statistical analysis of the cytotoxicity of derivative **10** in B16F10 cells 81
S74. Statistical analysis of the cytotoxicity of derivative **11** in B16F10 cells 82

S75. Statistical analysis of the inhibitory effect of derivatives **2–11**, KA, and PTU on melanin formation in B16F10 cells 83

S76. Statistical analysis of the inhibitory effect of derivative **2**, PTU, and KA on melanin formation in B16F10 cells 86

S77. Statistical analysis of the inhibitory effect of derivative **5**, PTU, and KA on melanin formation in B16F10 cells 88

S78. Statistical analysis of the inhibitory effect of derivative **6**, PTU, and KA on melanin formation in B16F10 cells 90

S79. Statistical analysis of the pigmentation inhibition effect of derivative **5** and KA on the zebrafish dorsal view 92

S80. Statistical analysis of the pigmentation inhibition effect of derivative **5** and KA on the zebrafish lateral view 93

S81. Statistical analysis of the cytotoxicity of derivative **1** in Hs27 cells 94

S82. Statistical analysis of the cytotoxicity of derivative **2** in Hs27 cells 95

S83. Statistical analysis of the cytotoxicity of derivative **3** in Hs27 cells 96

S84. Statistical analysis of the cytotoxicity of derivative **4** in Hs27 cells 97

S85. Statistical analysis of the cytotoxicity of derivative **5** in Hs27 cells 98

S86. Statistical analysis of the cytotoxicity of derivative **6** in Hs27 cells 99

S87. Statistical analysis of the cytotoxicity of derivative **7** in Hs27 cells 100

S88. Statistical analysis of the cytotoxicity of derivative **8** in Hs27 cells 101

S89. Statistical analysis of the cytotoxicity of derivative **9** in Hs27 cells 102

S90. Statistical analysis of the cytotoxicity of derivative **10** in Hs27 cells 103

S91. Statistical analysis of the cytotoxicity of derivative **11** in Hs27 cells............................ 104

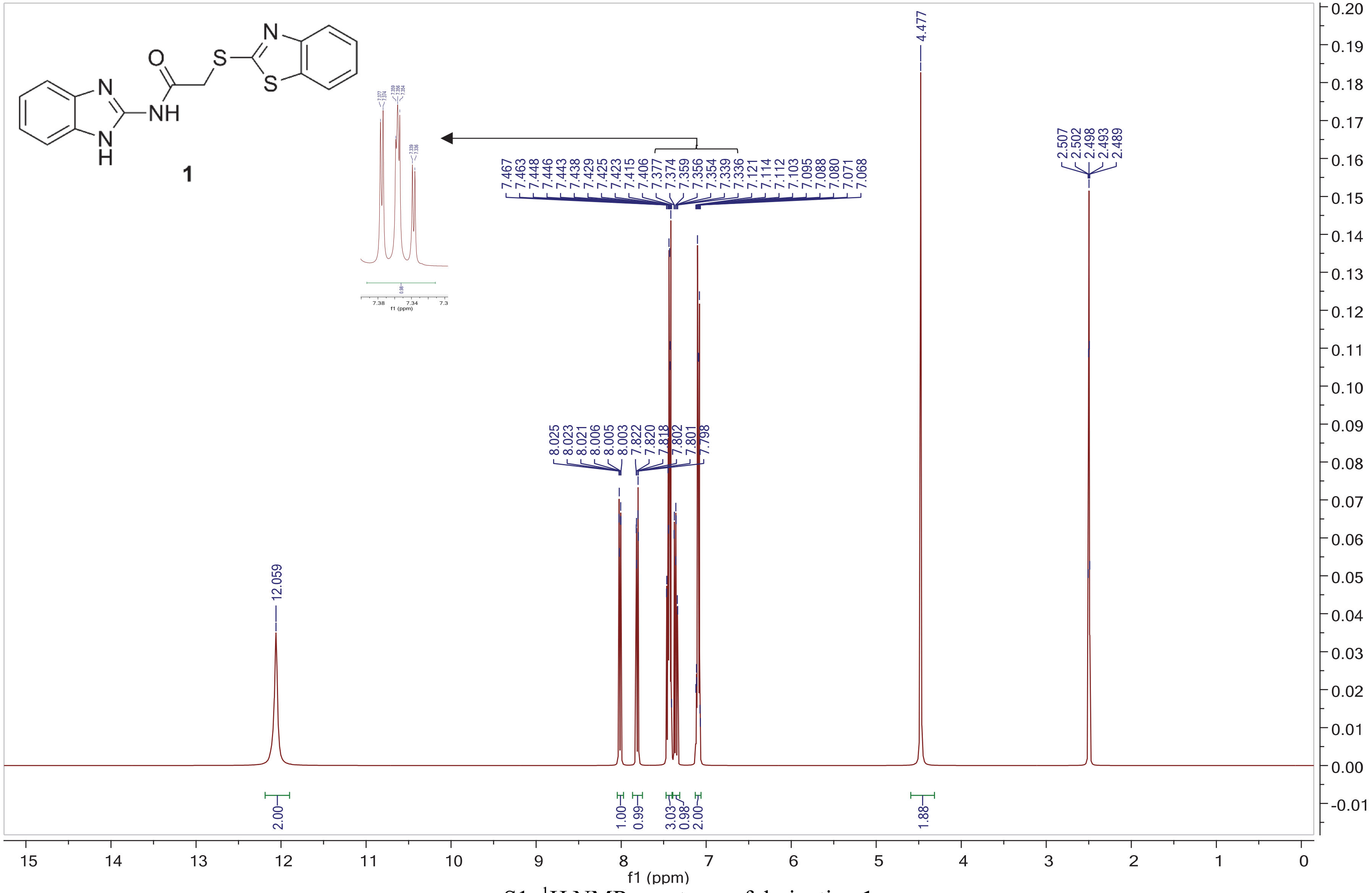

S1. ^{1}H NMR spectrum of derivative **1**

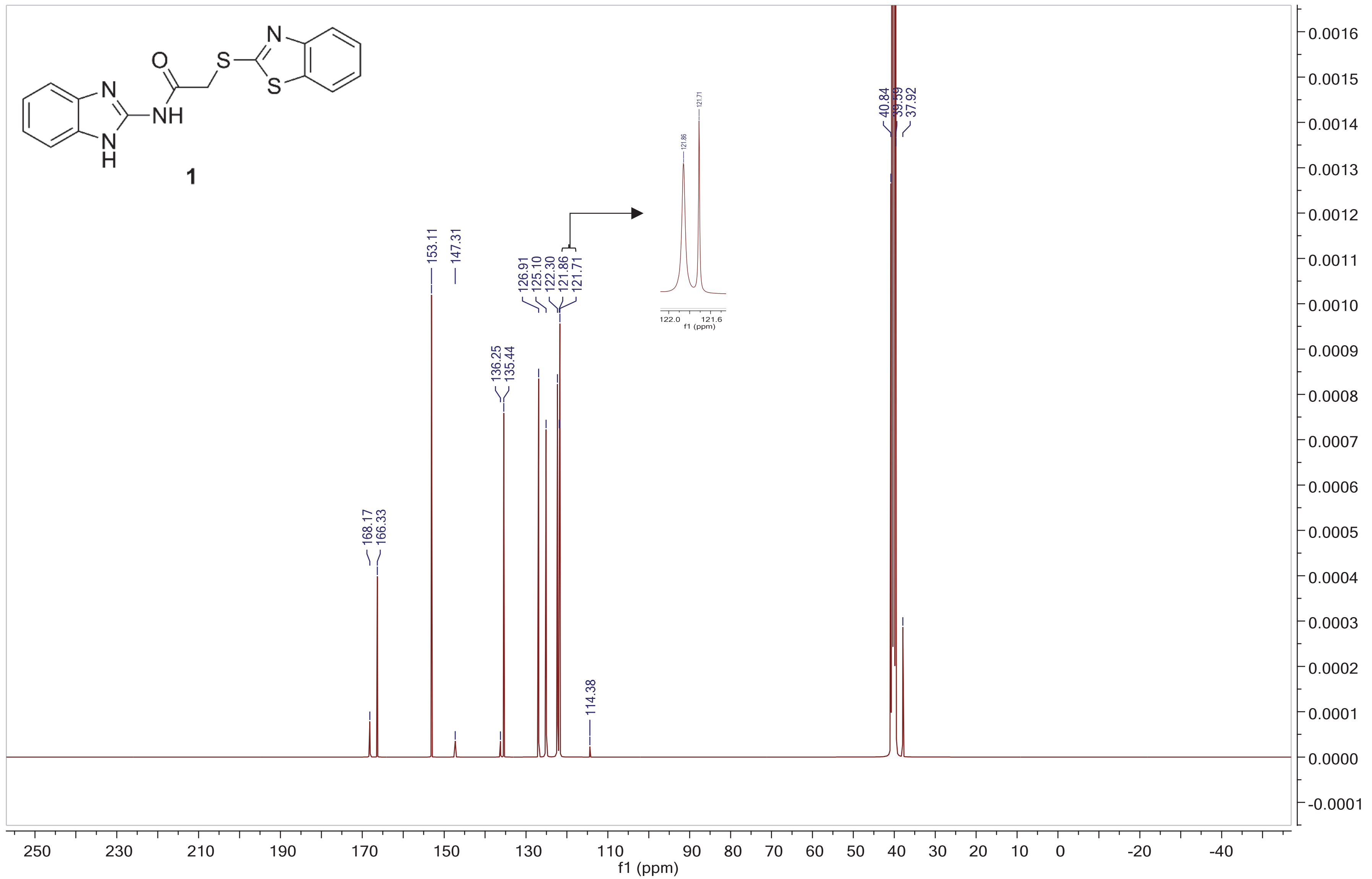

S2. ^{13}C NMR spectrum of derivative **1**

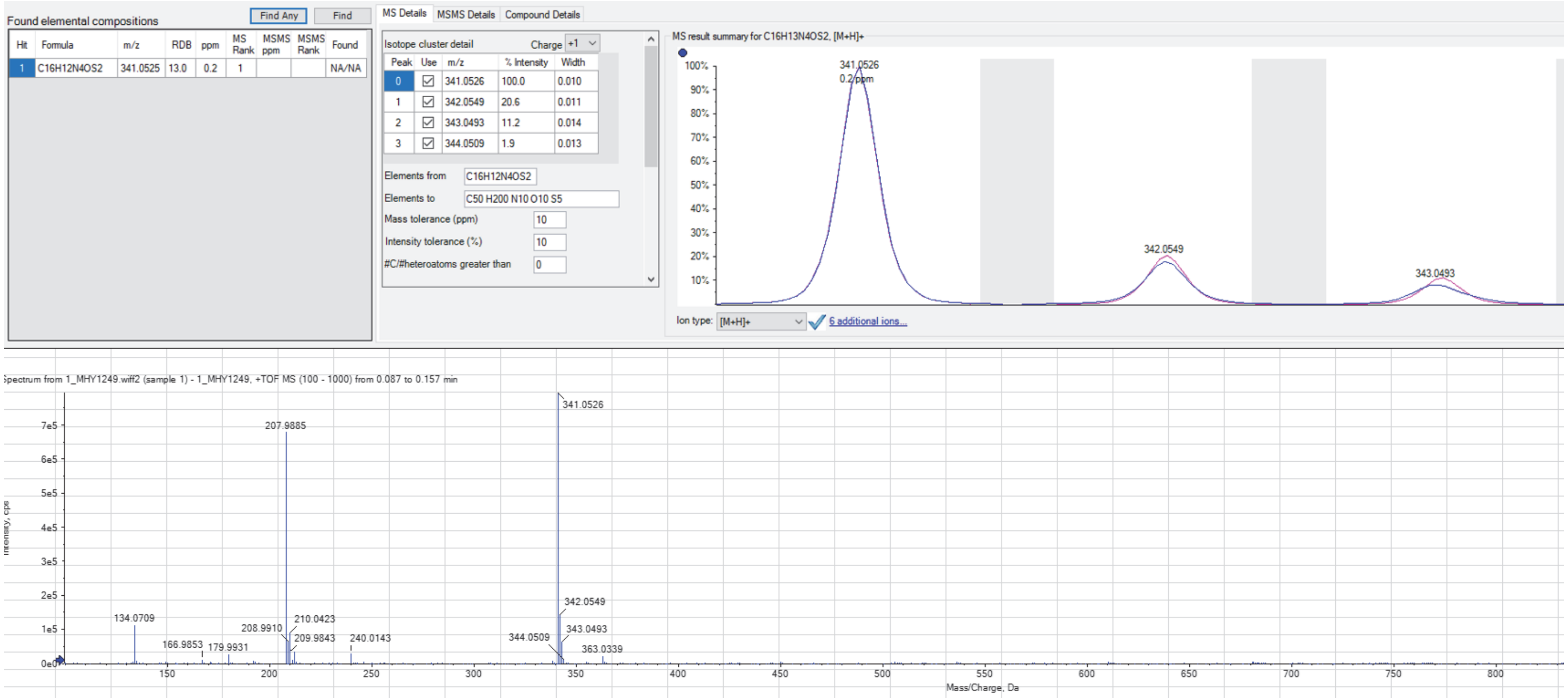

S3. HRMS spectrum of derivative **1**

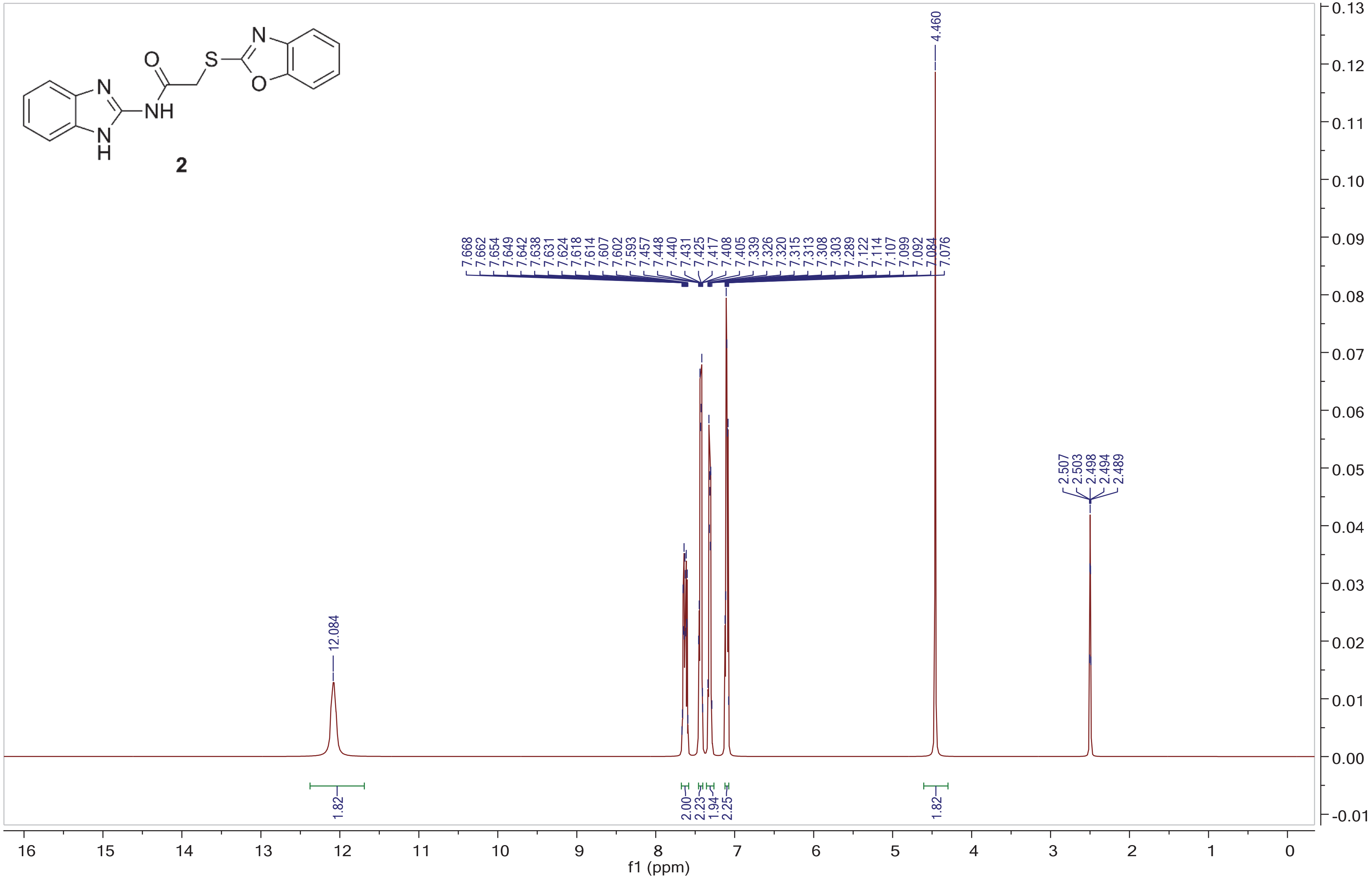

S4. ^{1}H NMR spectrum of derivative **2**

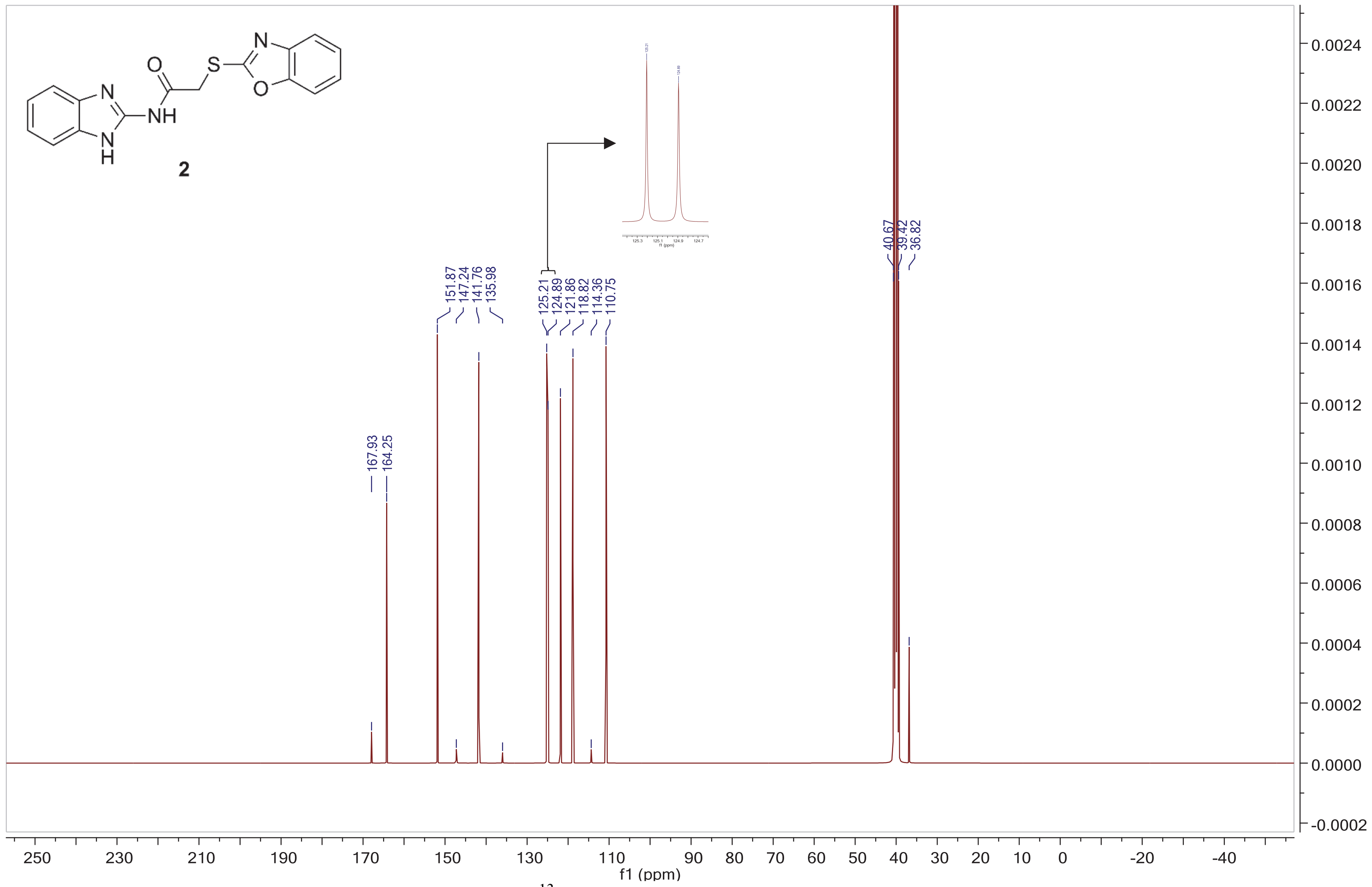

S5. ^{13}C NMR spectrum of derivative **2**

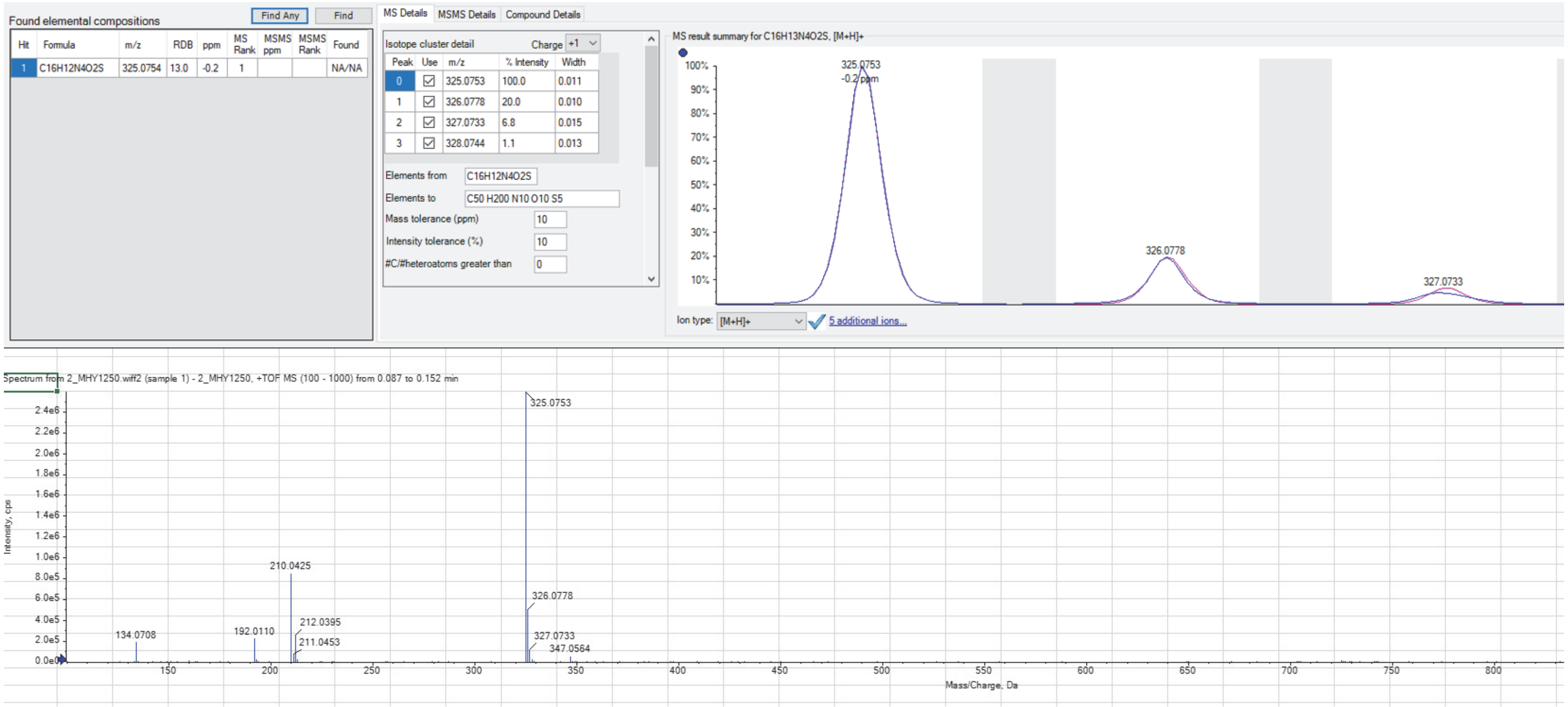

S6. HRMS spectrum of derivative **2**

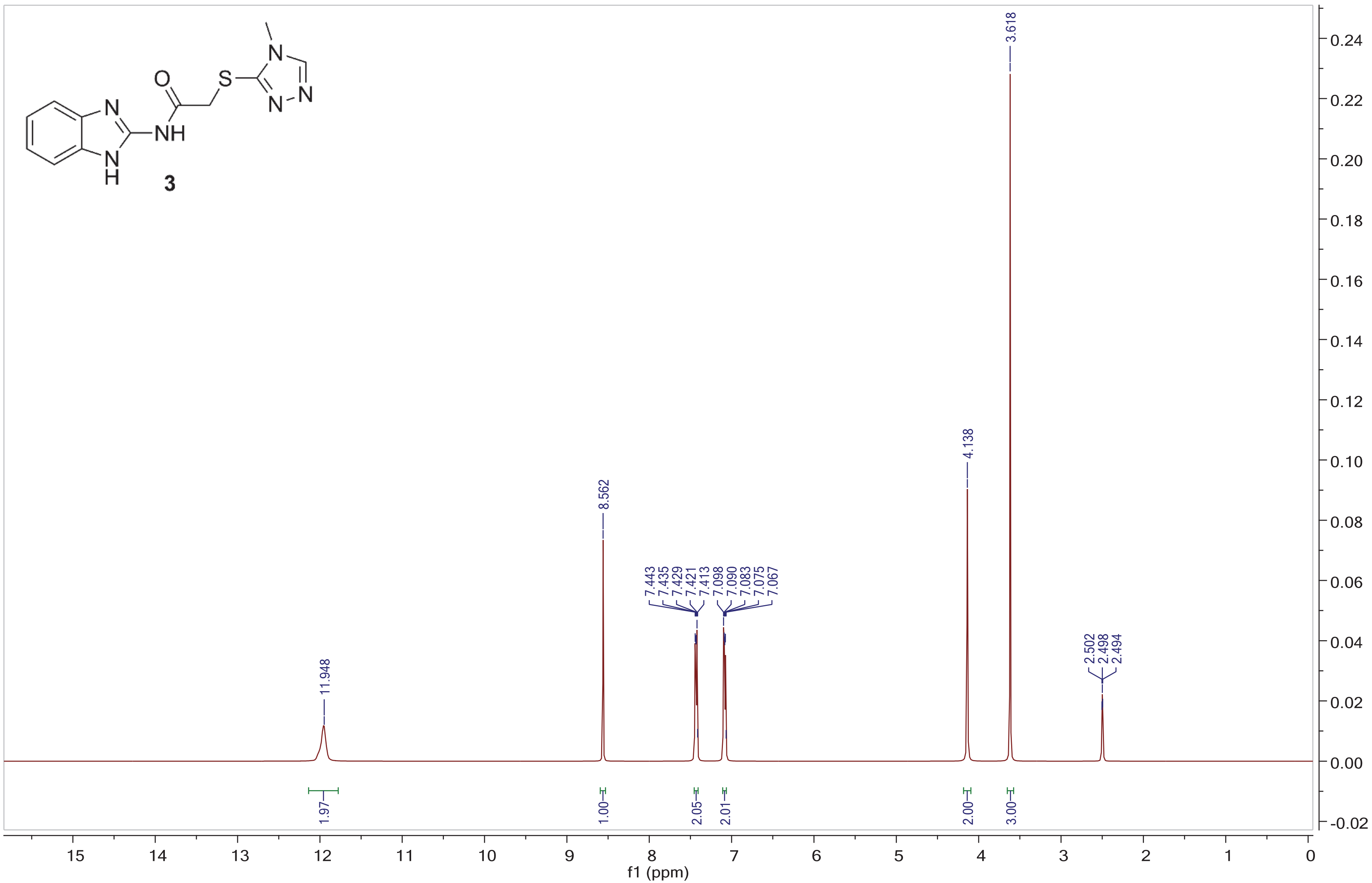

S7. ^{1}H NMR spectrum of derivative **3**

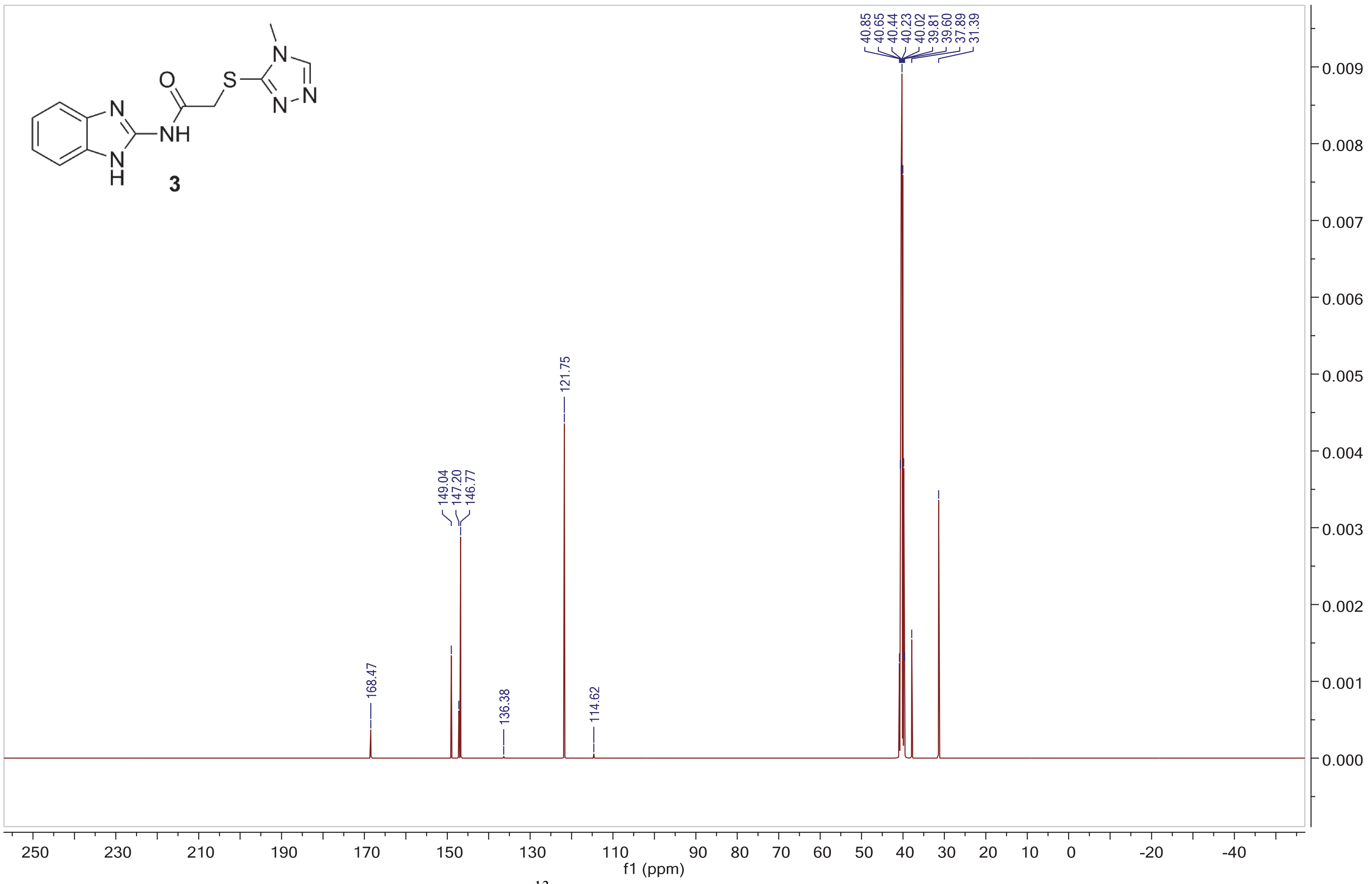

S8. ^{13}C NMR spectrum of derivative **3**

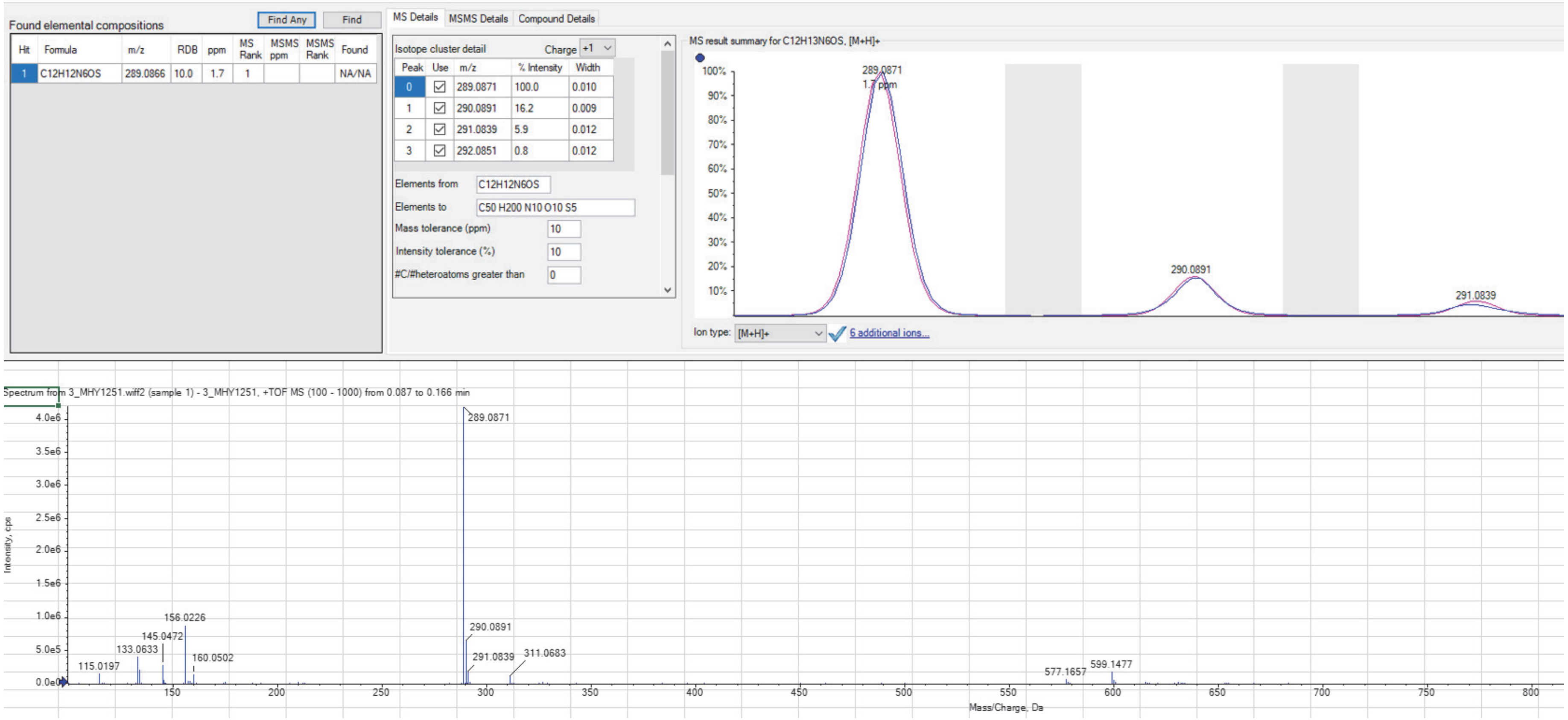

S9. HRMS spectrum of derivative **3**

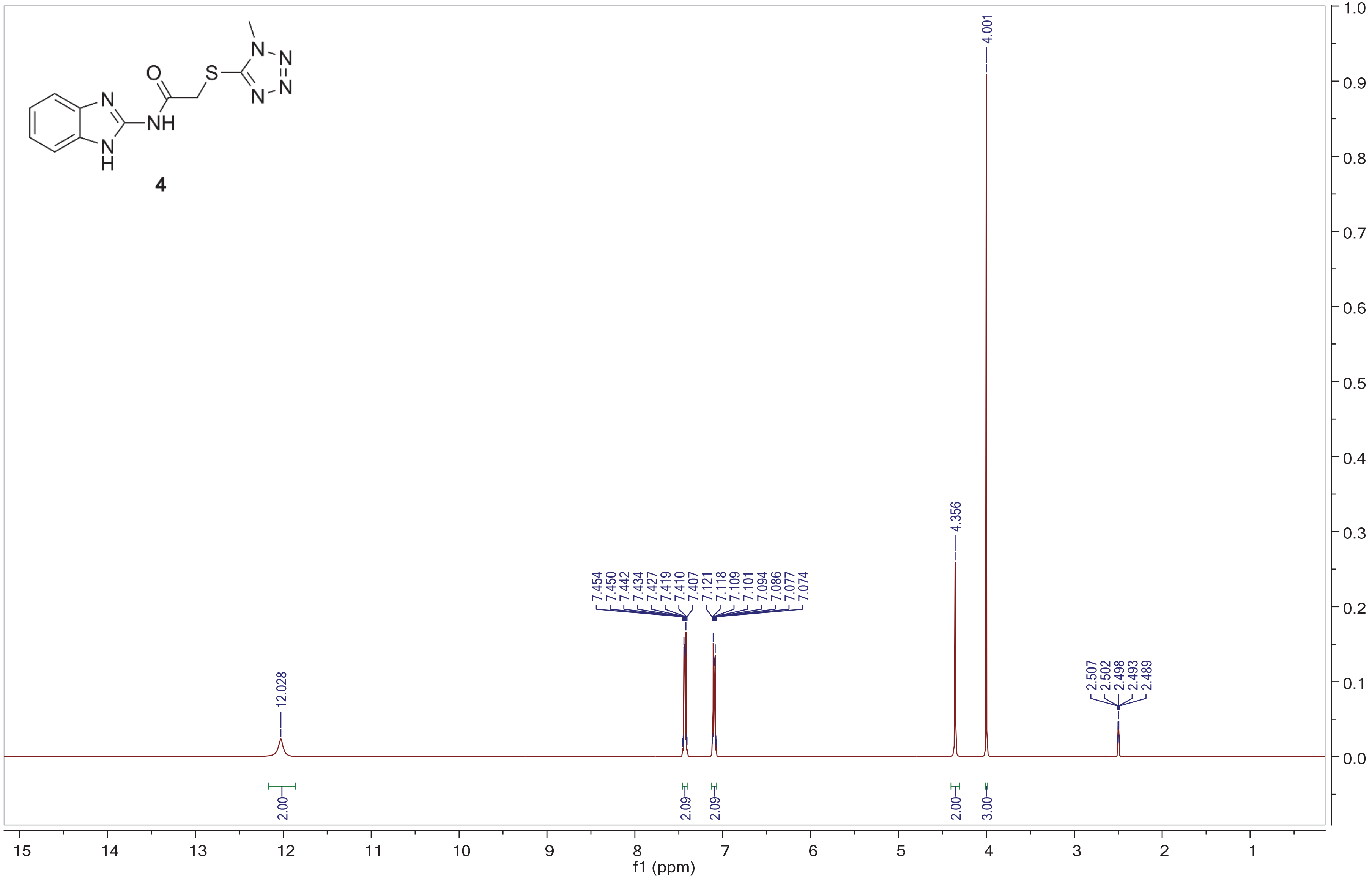

S10. ^{1}H NMR spectrum of derivative **4**

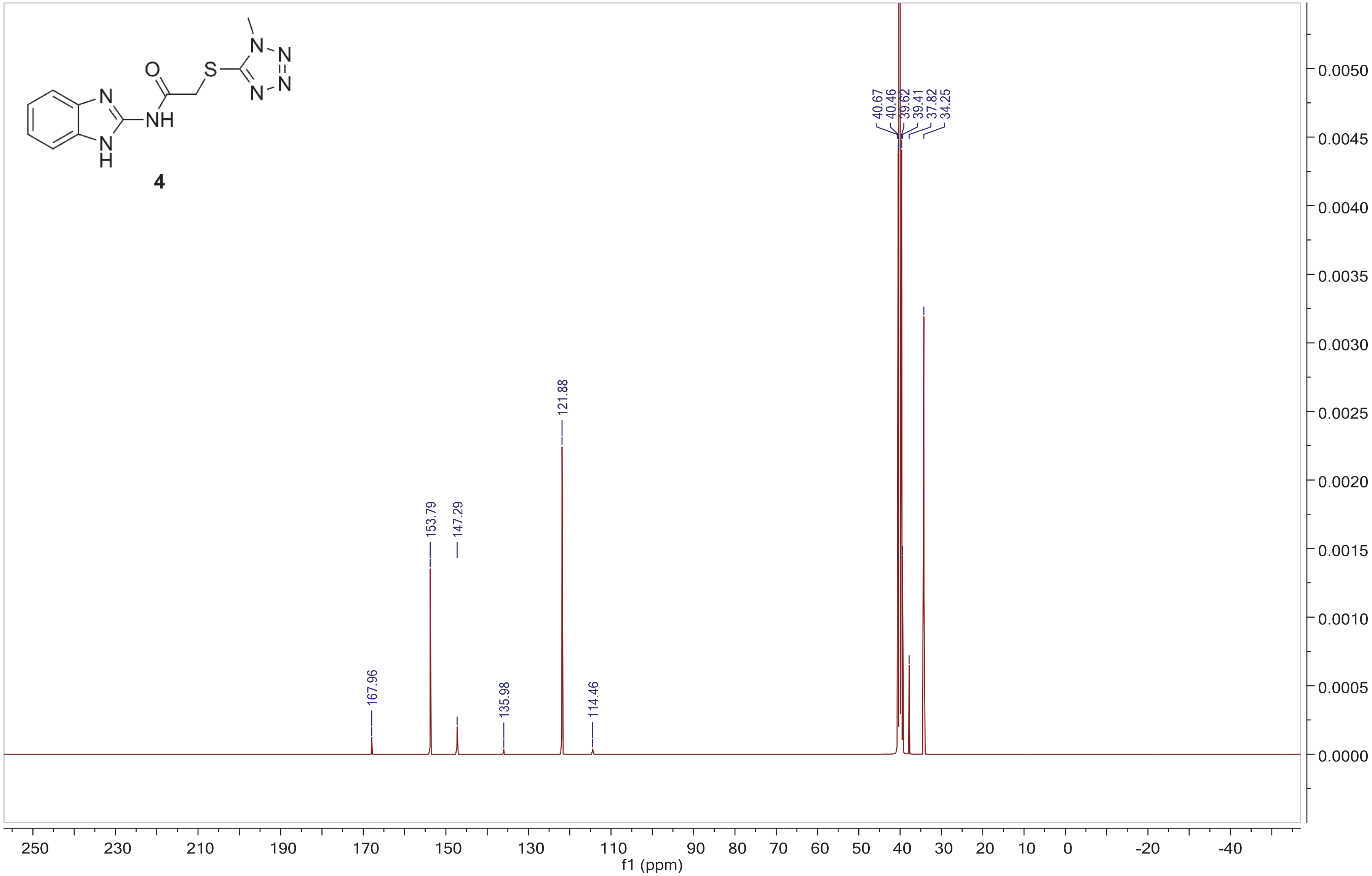

S11. ^{13}C NMR spectrum of derivative **4**

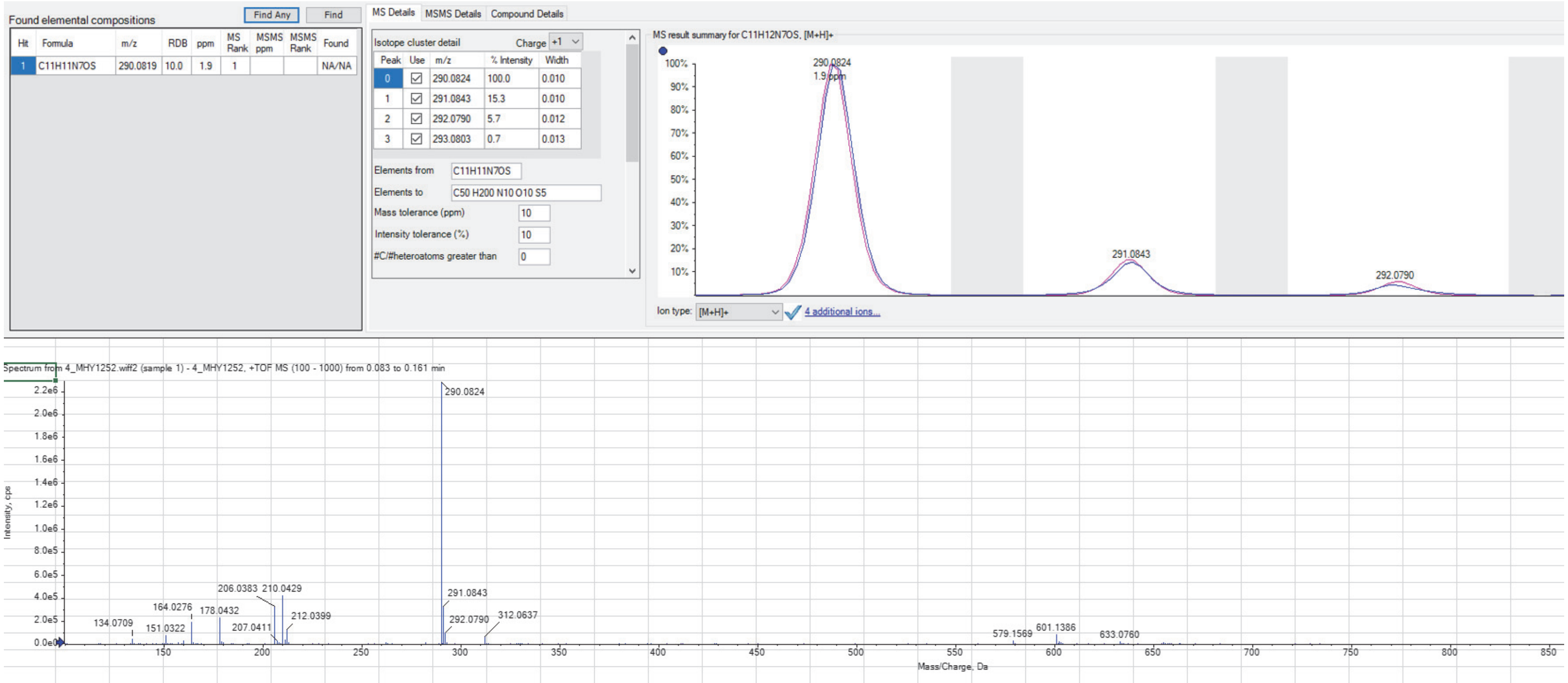

S12. HRMS spectrum of derivative **4**

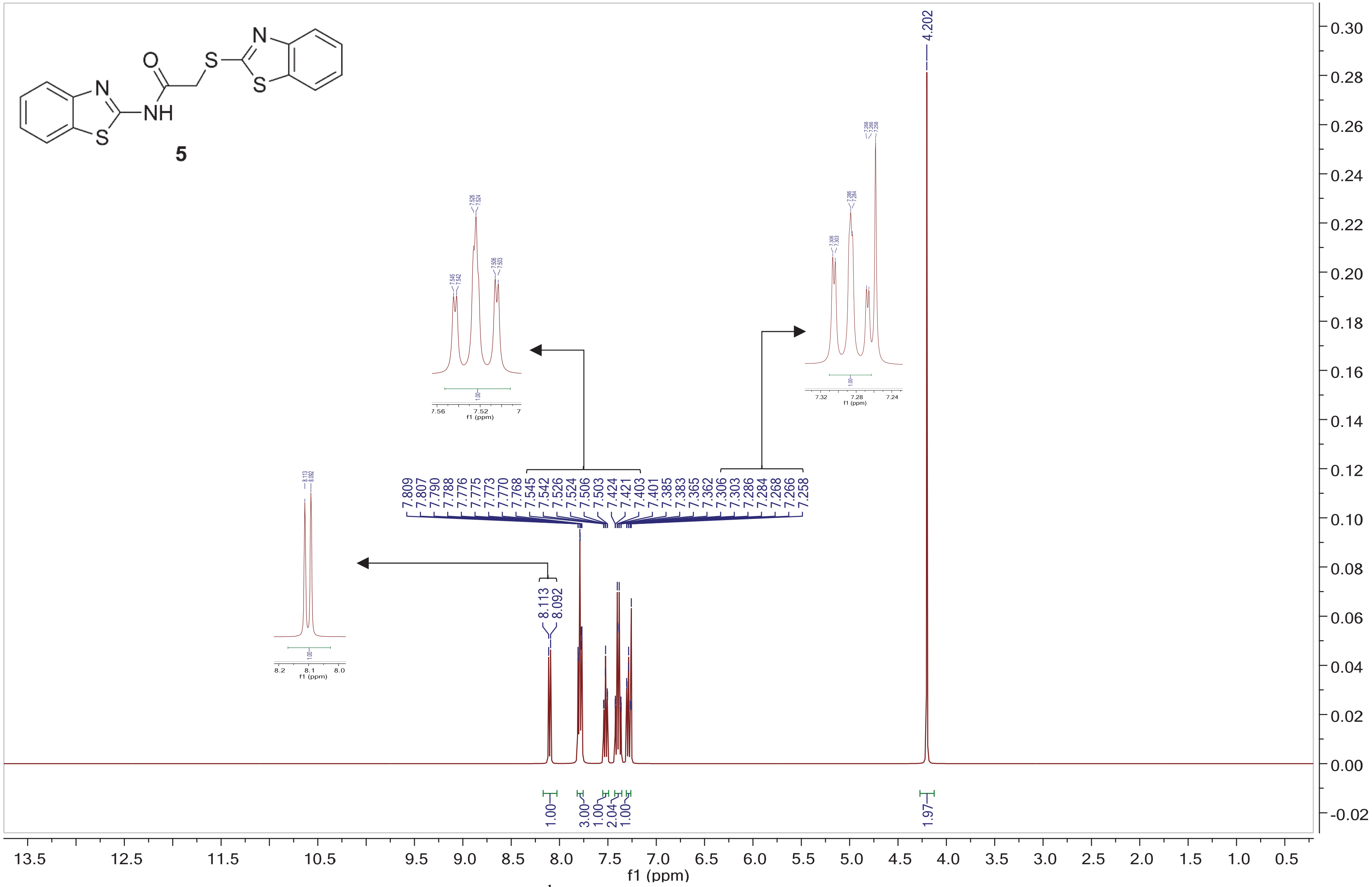

S13. ^{1}H NMR spectrum of derivative **5**

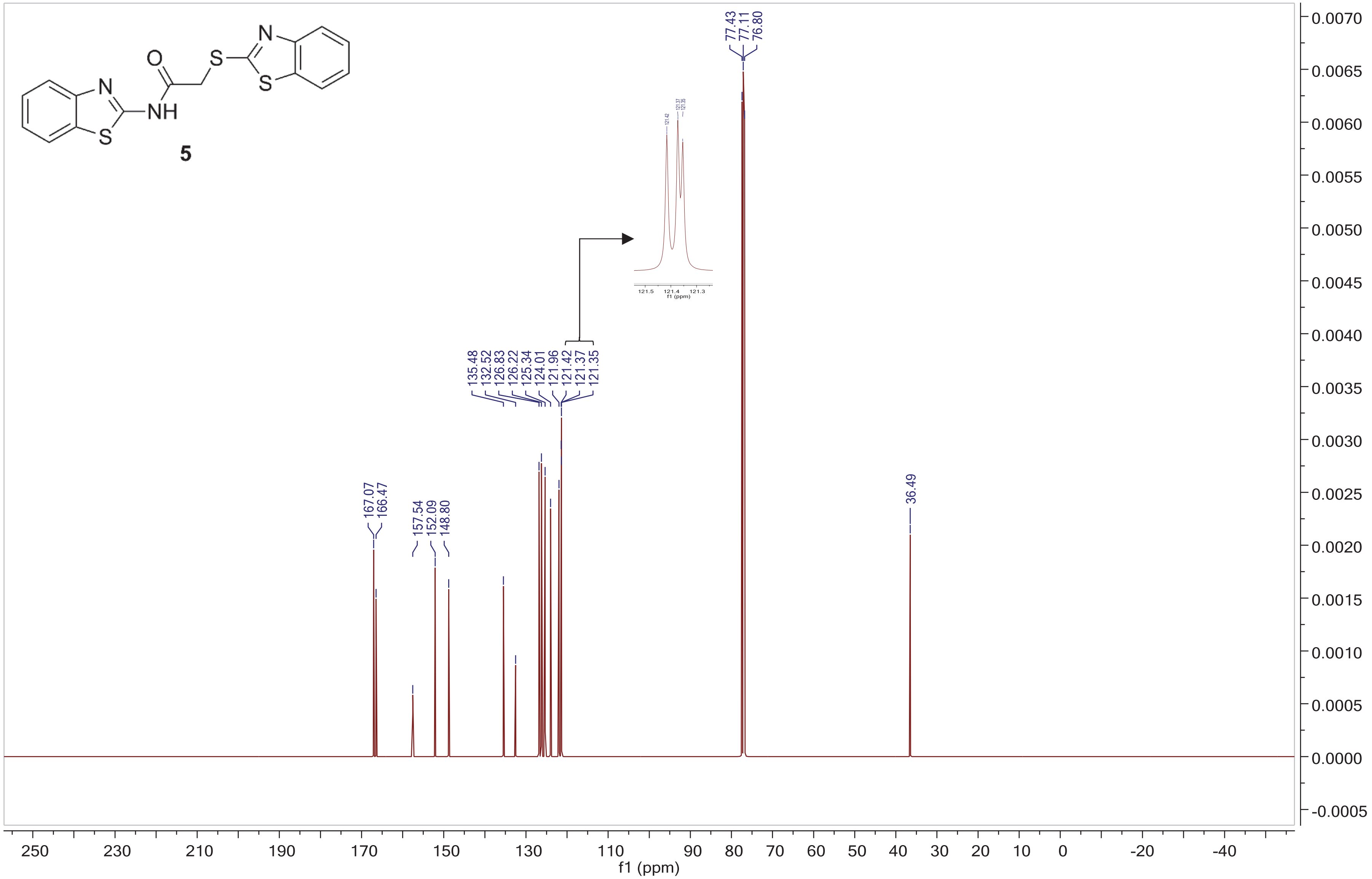

S14. ^{13}C NMR spectrum of derivative **5**

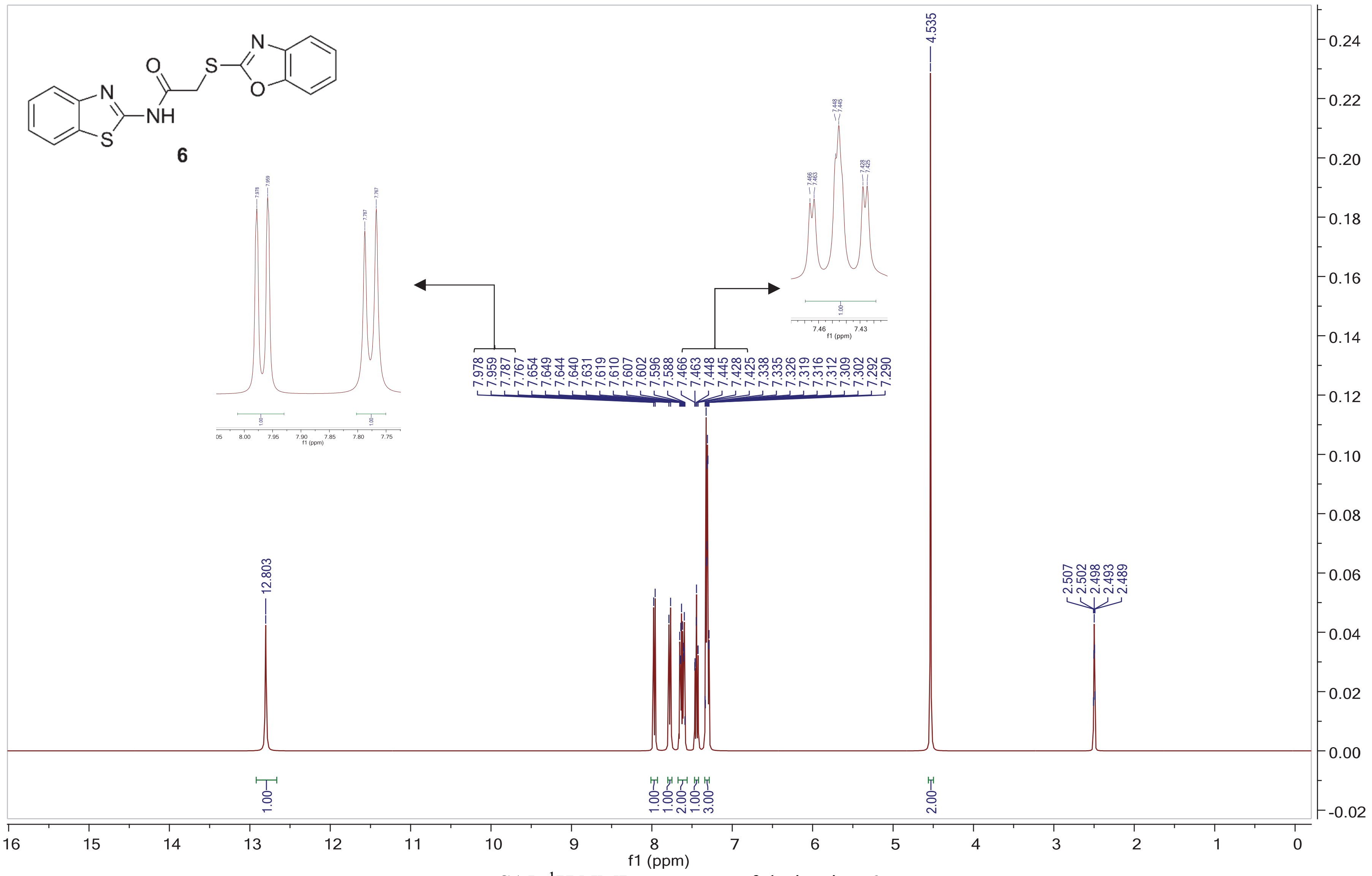

S15. ^{1}H NMR spectrum of derivative **6**

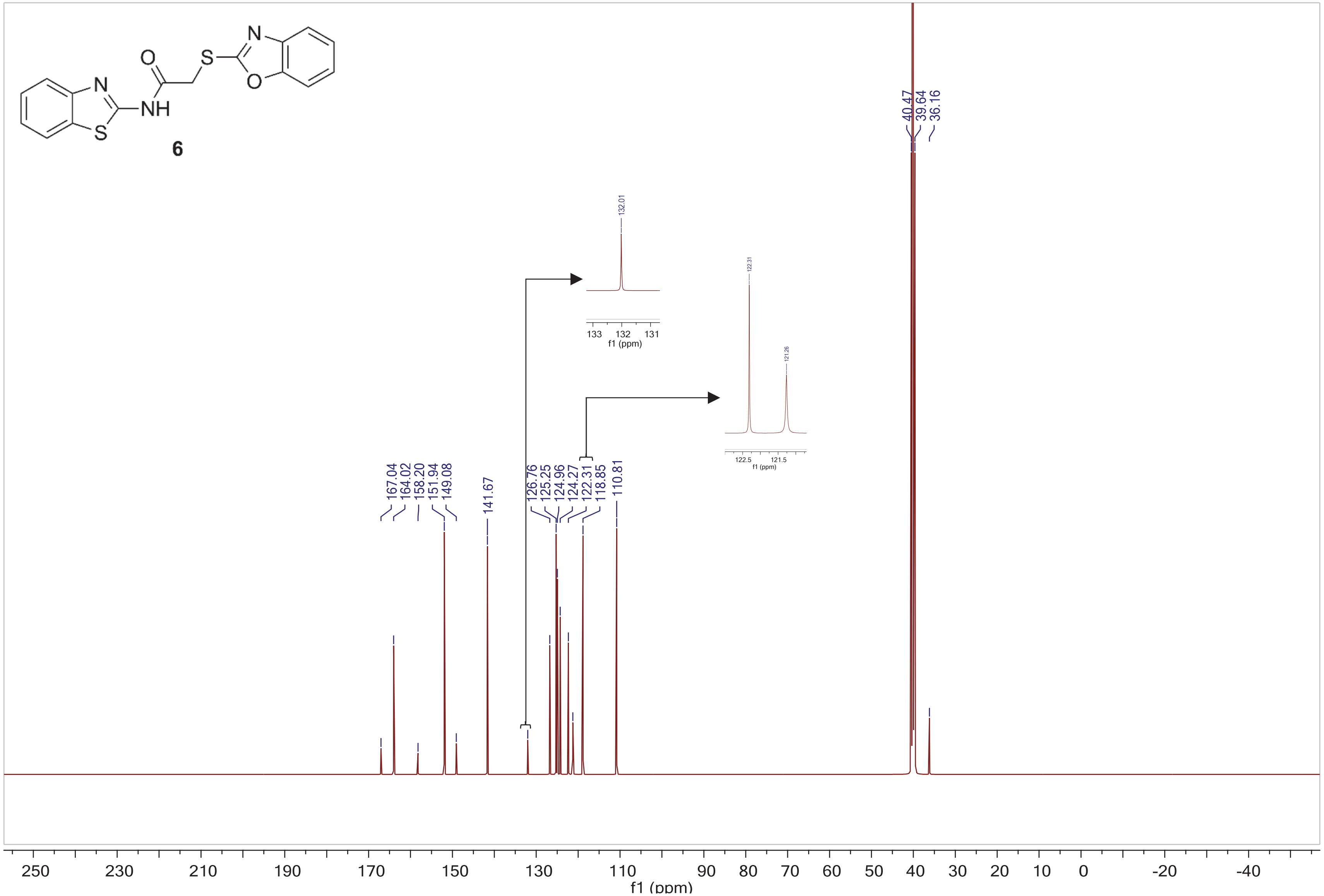

6

S16. ^{13}C NMR spectrum of derivative **6**

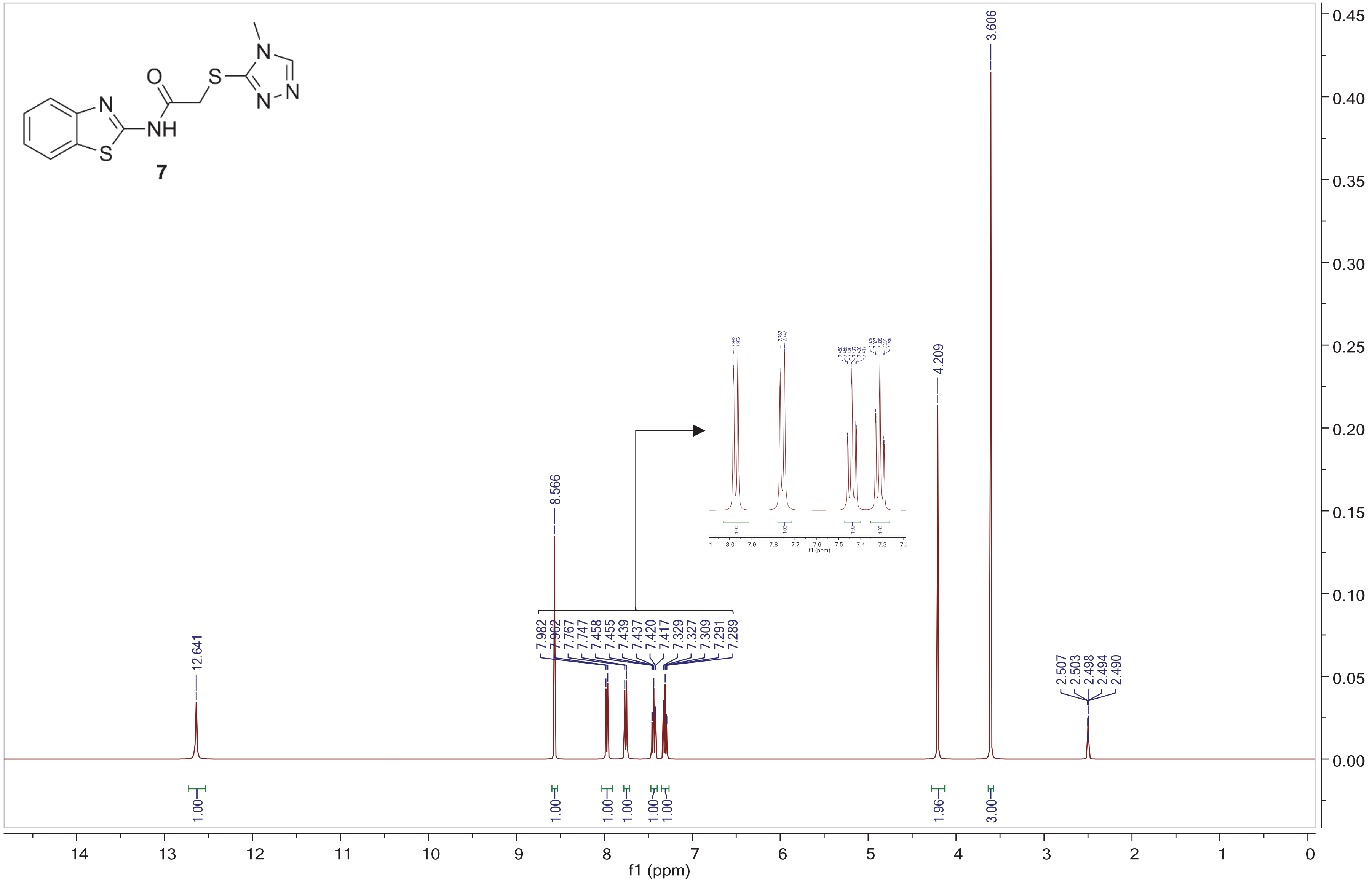

S17. ^{1}H NMR spectrum of derivative **7**

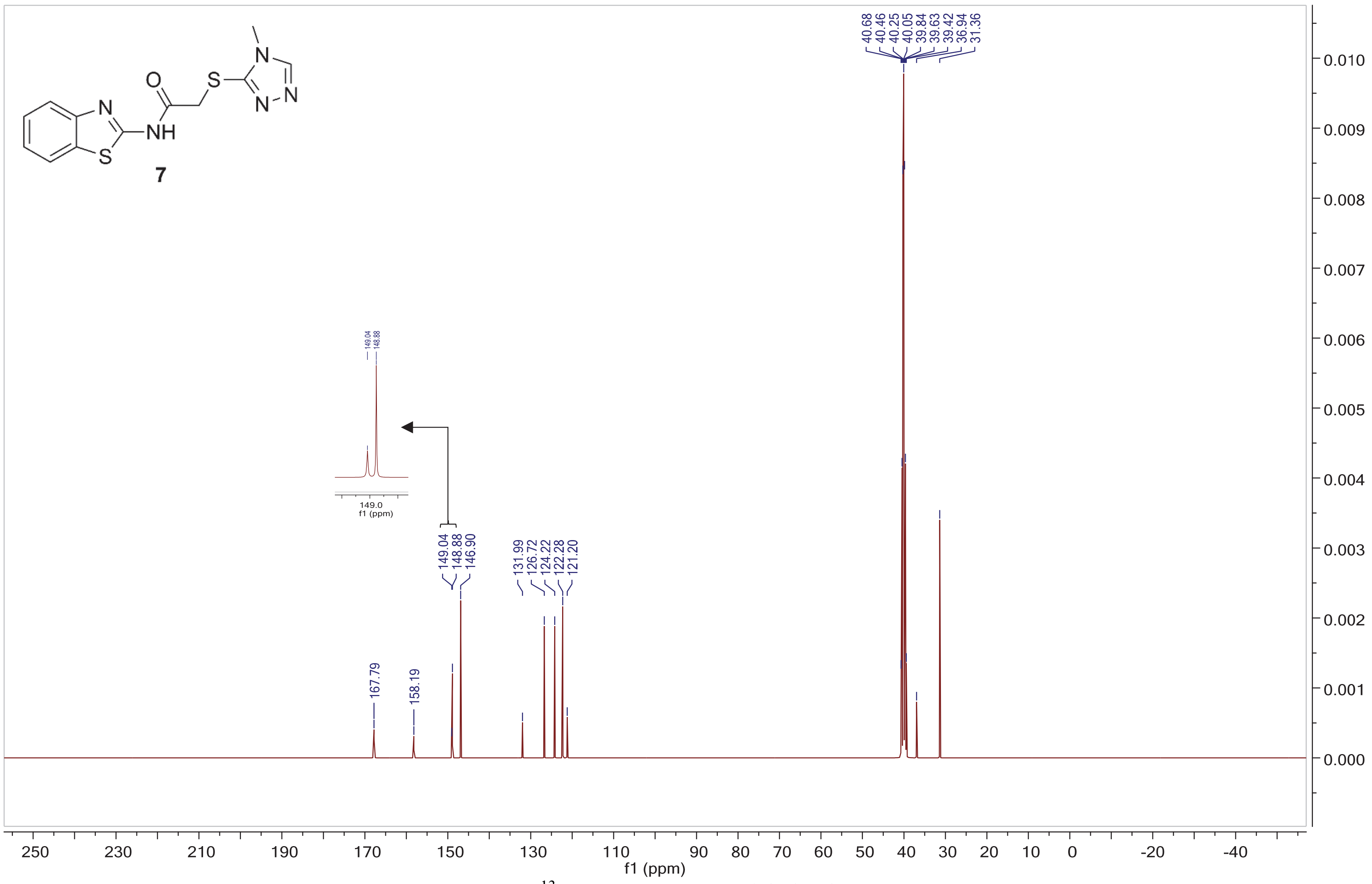

S18. ^{13}C NMR spectrum of derivative **7**

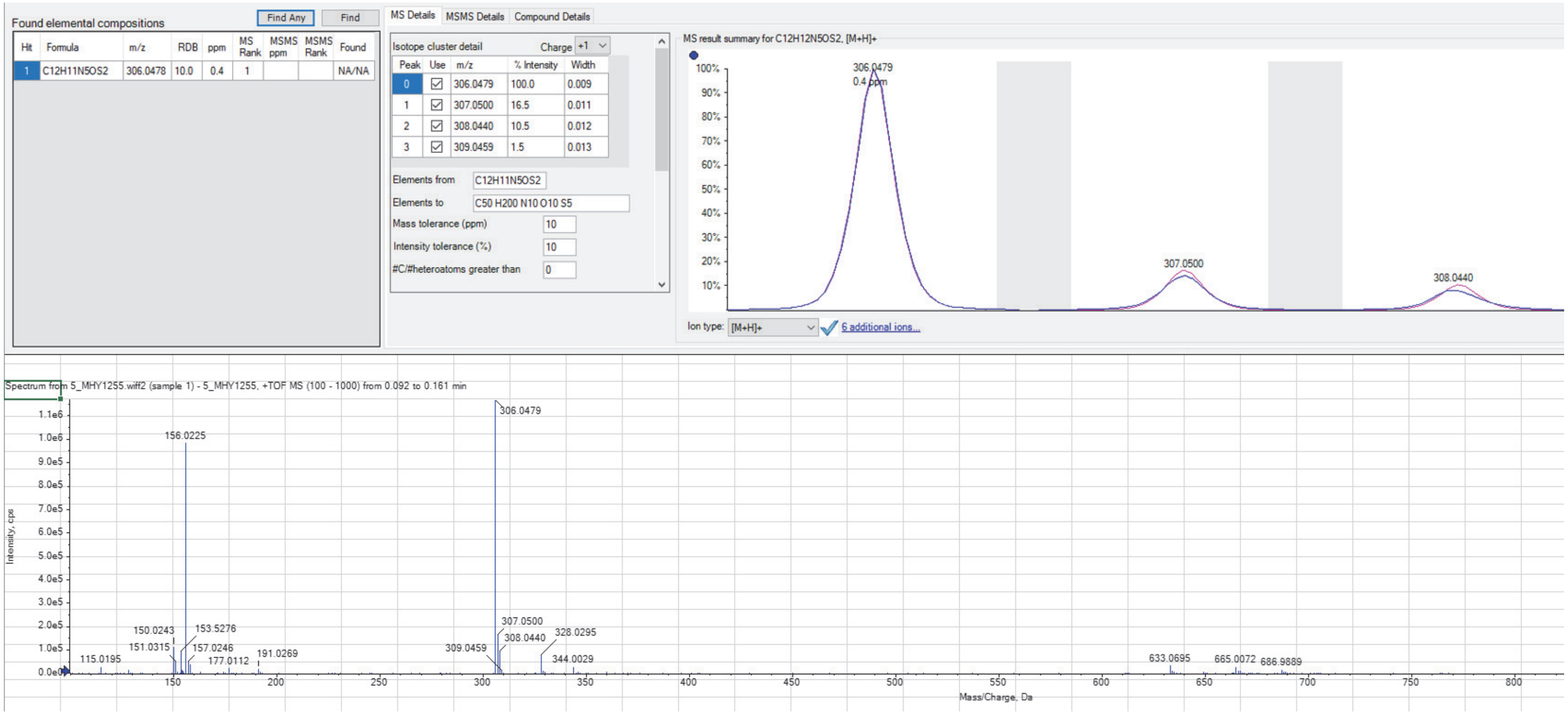

S19. HRMS spectrum of derivative **7**

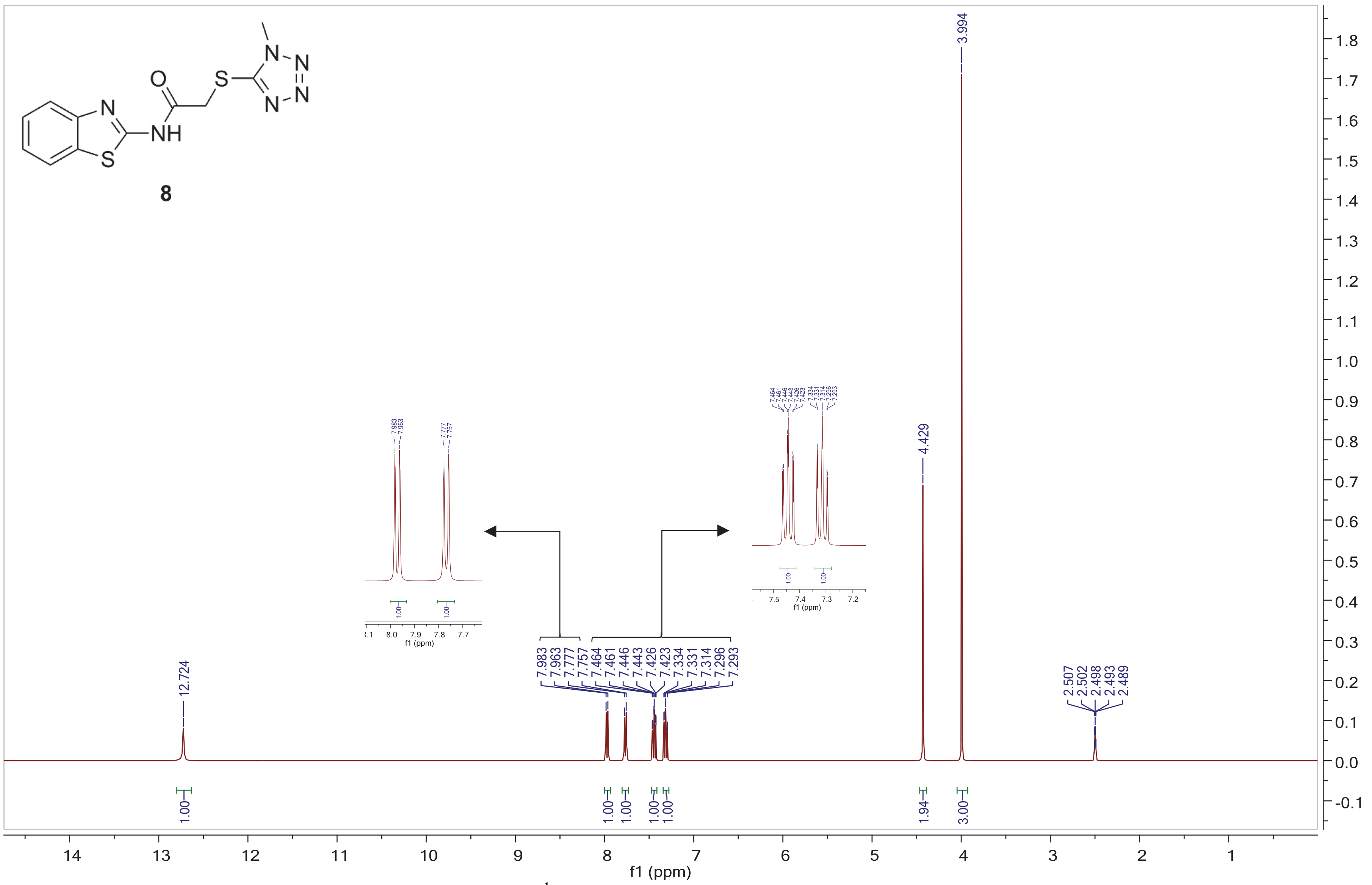

S20. ^{1}H NMR spectrum of derivative **8**

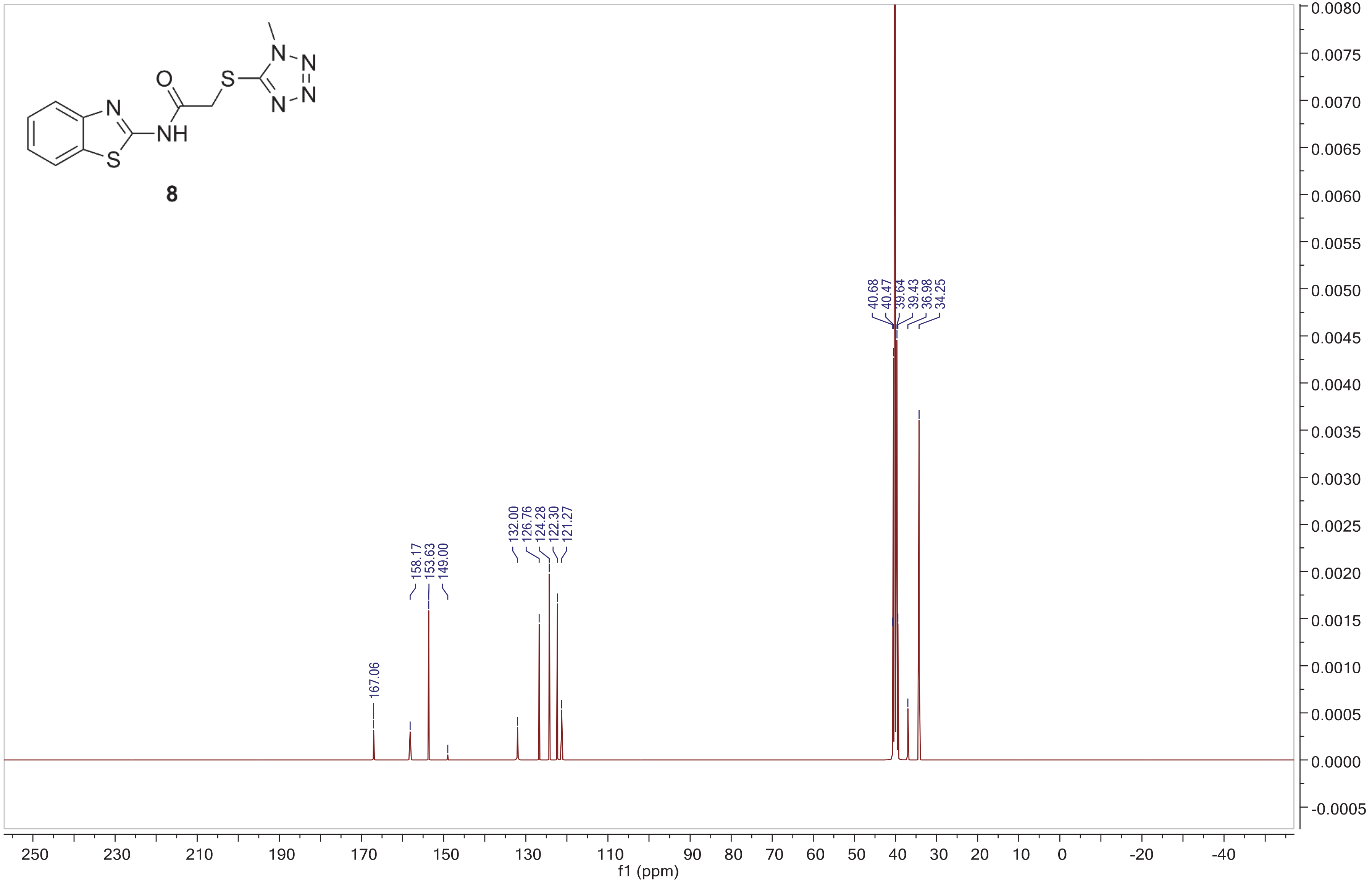

S21. ^{13}C NMR spectrum of derivative **8**

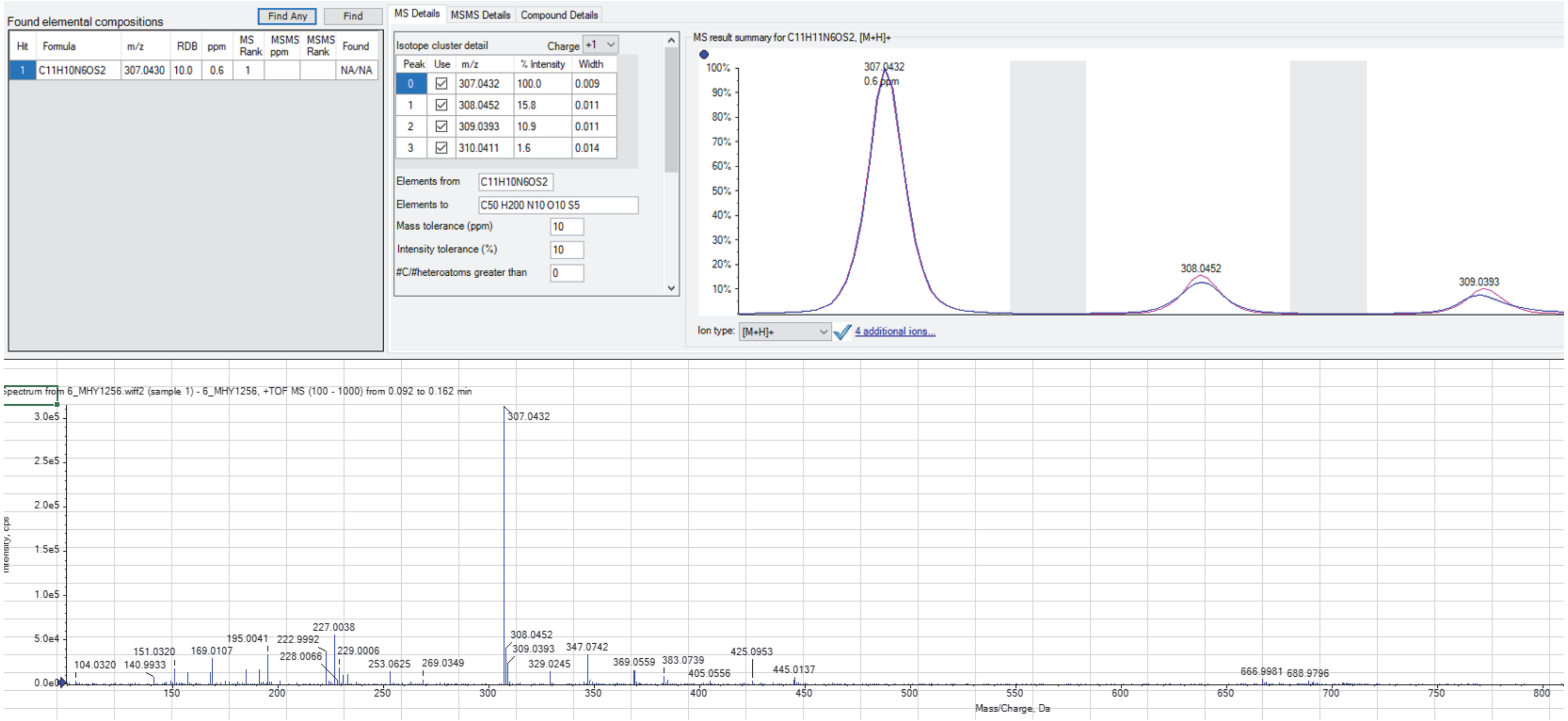

S22. HRMS spectrum of derivative **8**

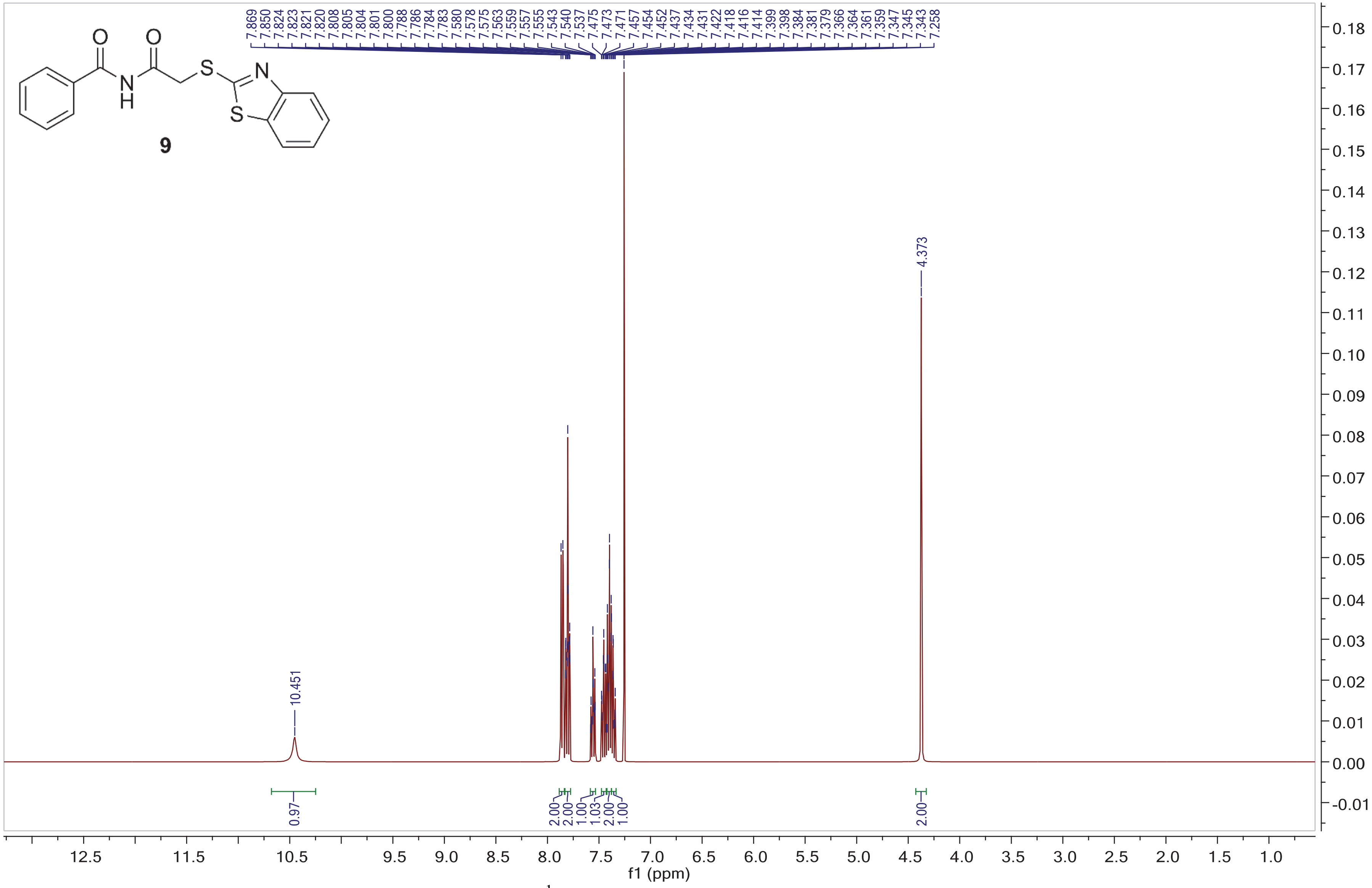

S23. ^{1}H NMR spectrum of derivative **9**

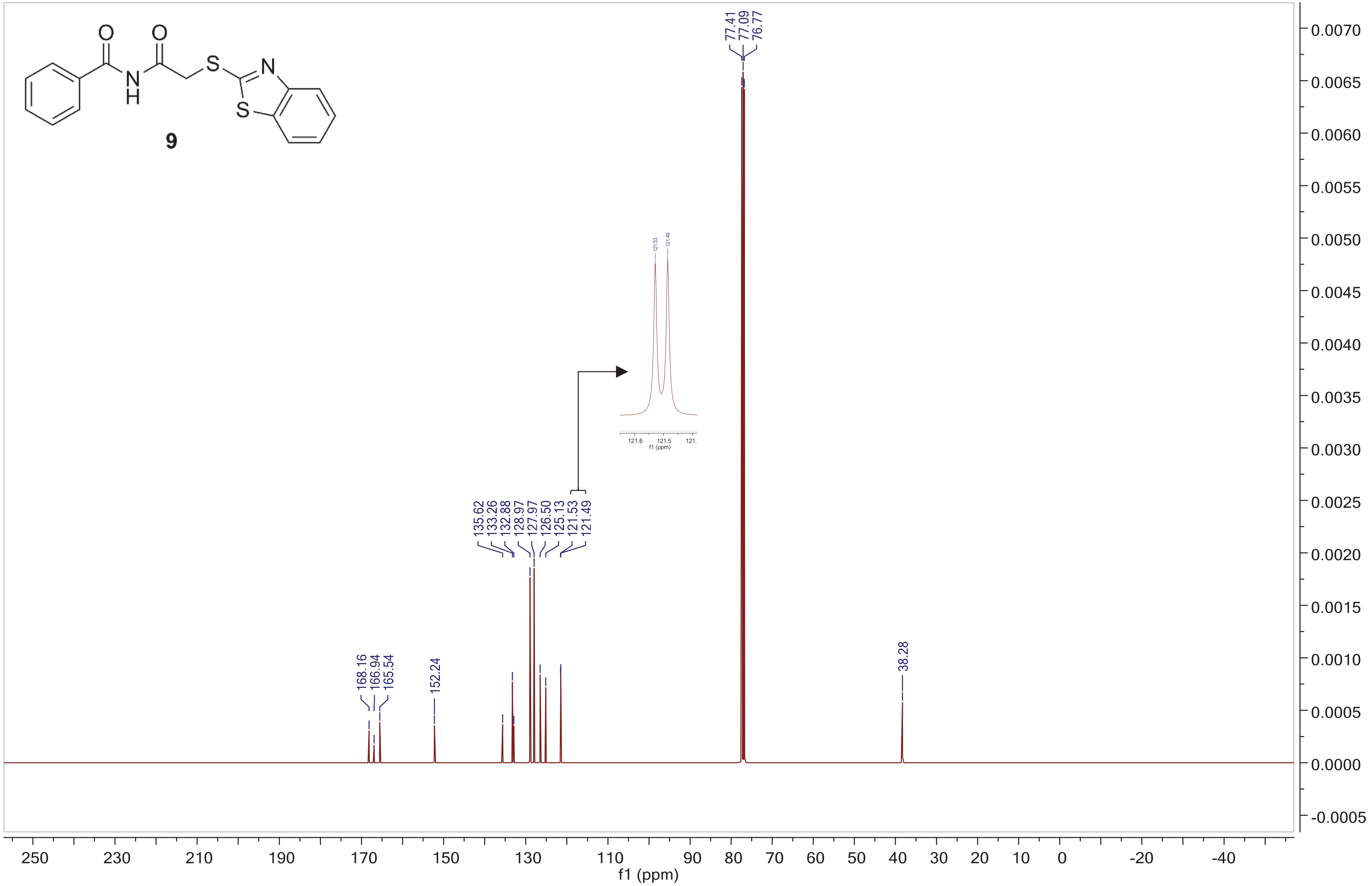

S24. ^{13}C NMR spectrum of derivative **9**

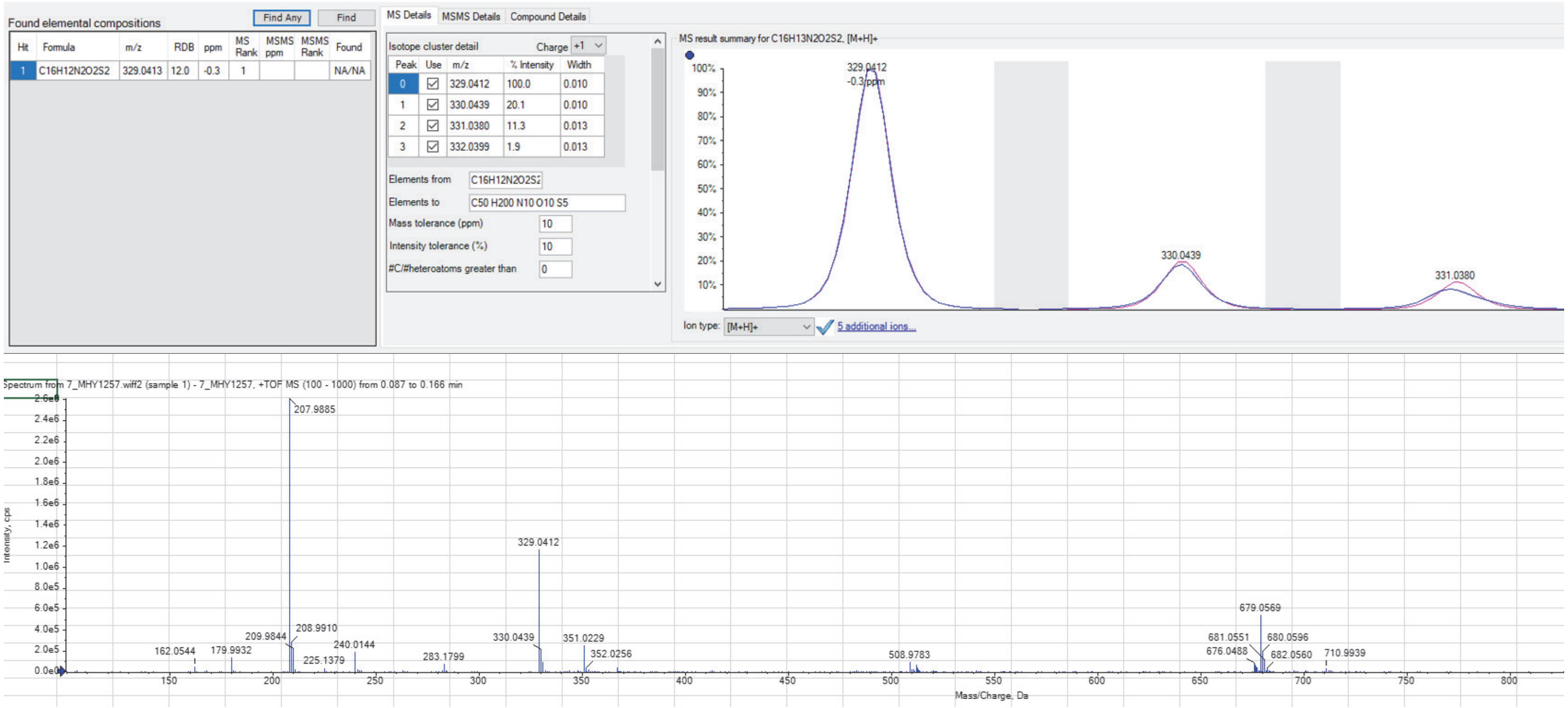

S25. HRMS spectrum of derivative **9**

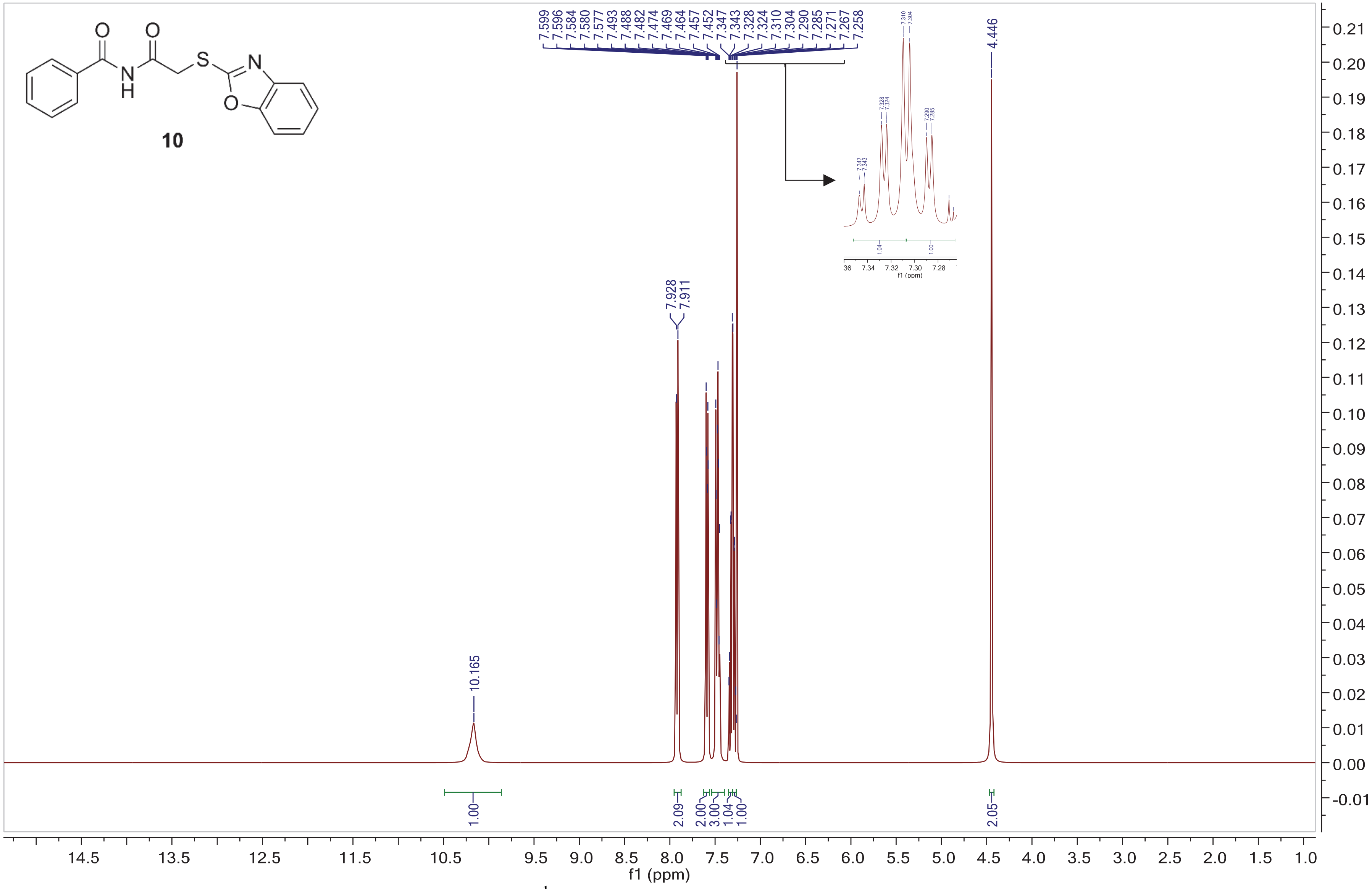

S26. ^{1}H NMR spectrum of derivative **10**

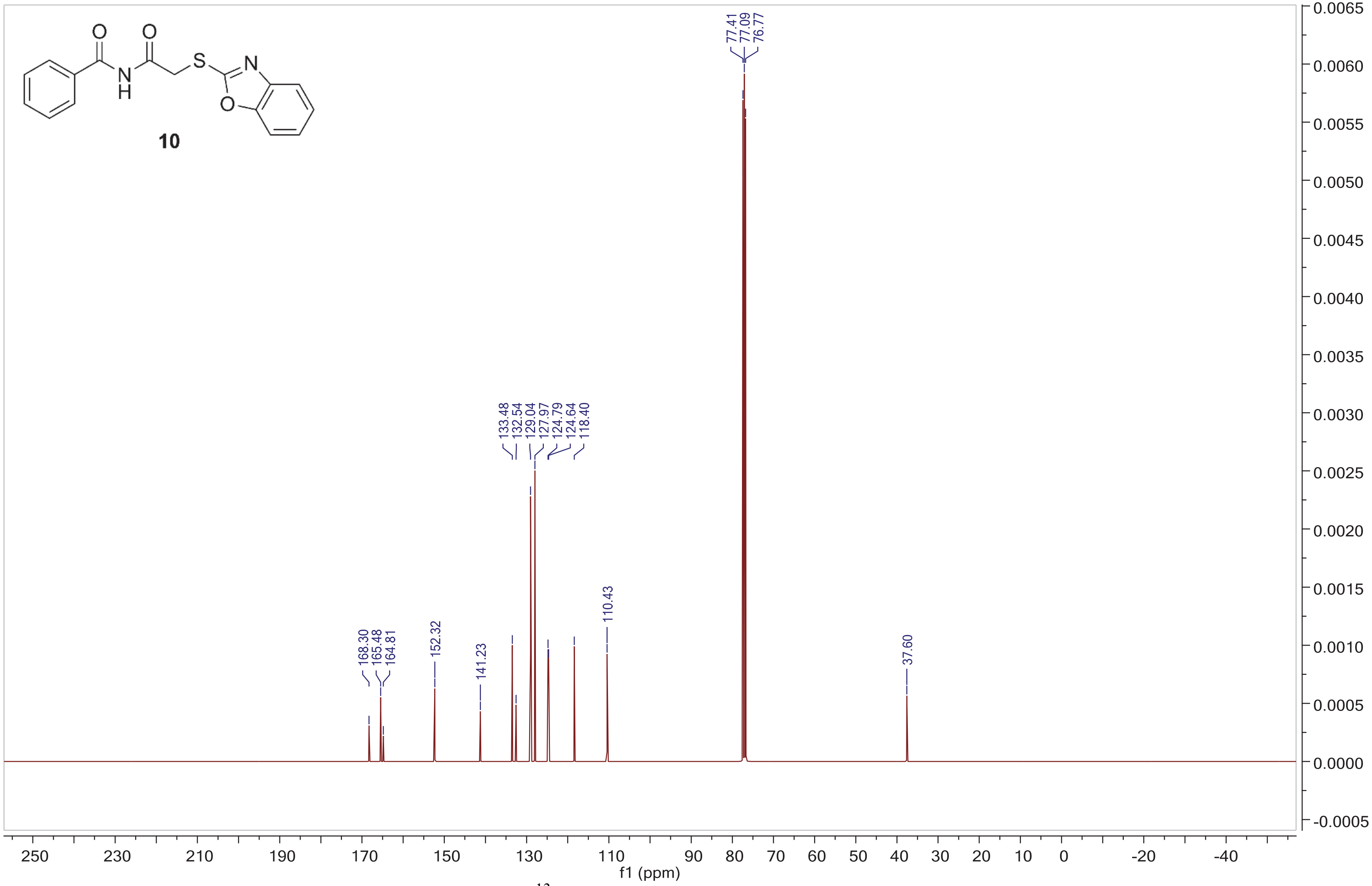

S27. ^{13}C NMR spectrum of derivative **10**

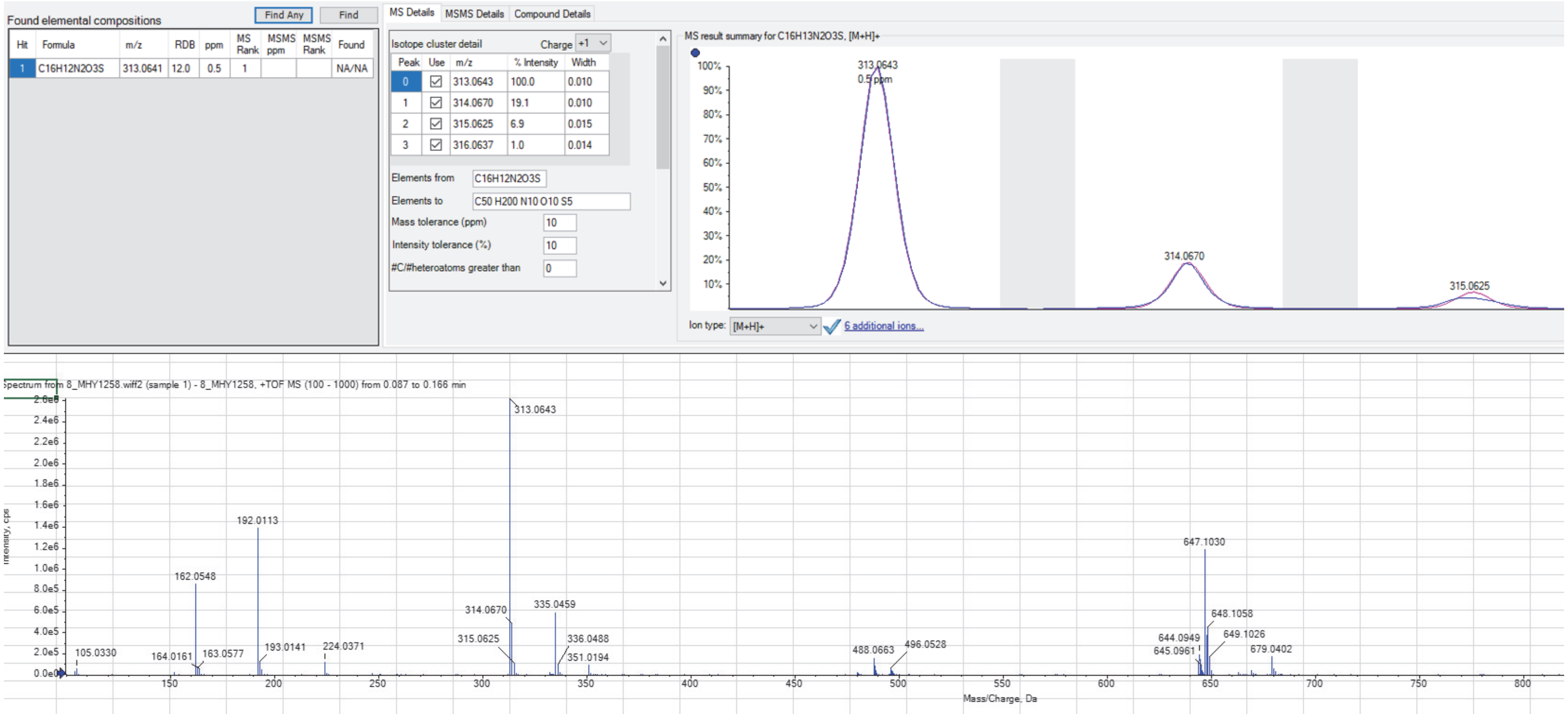

S28. HRMS spectrum of derivative **10**

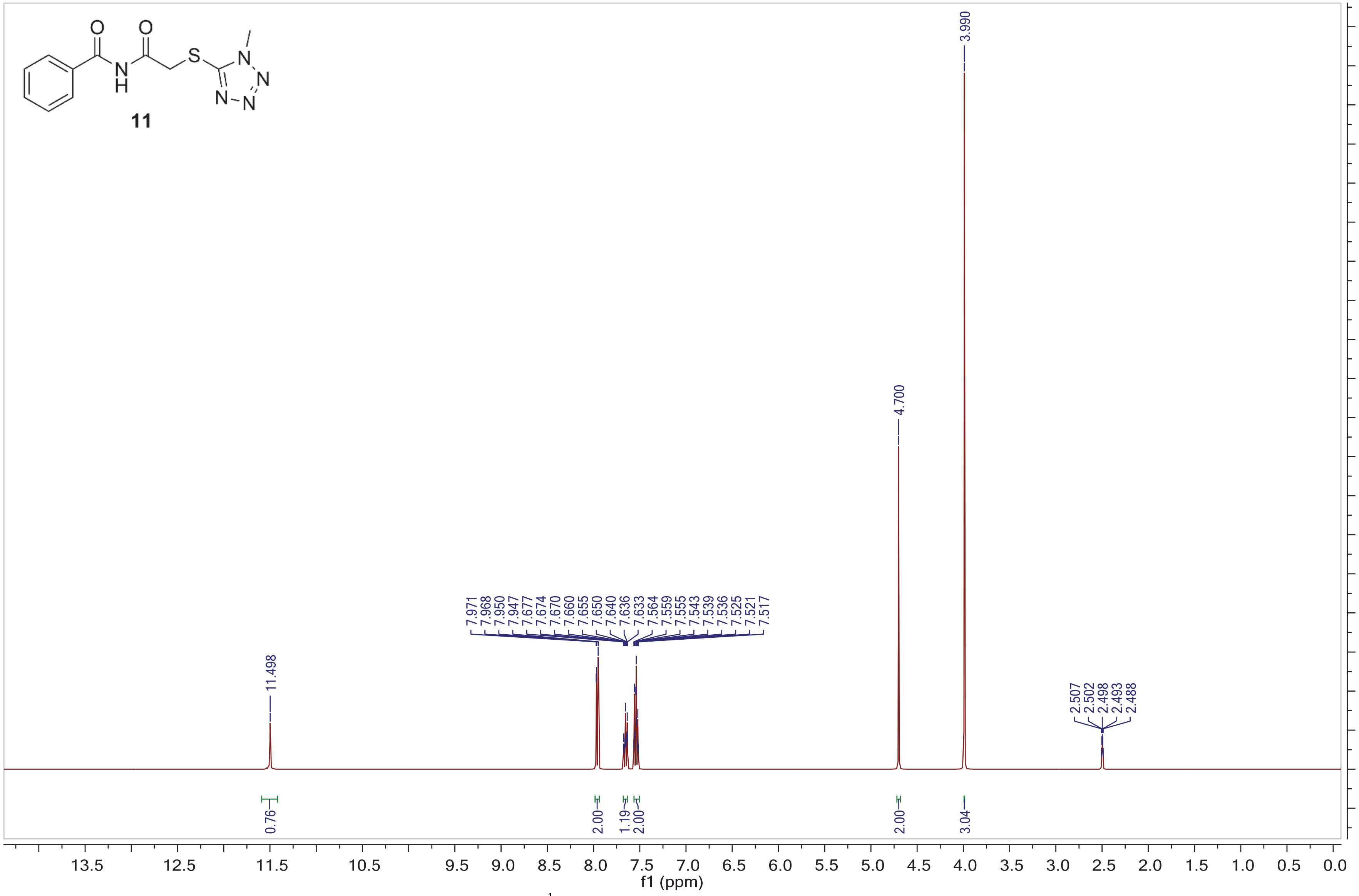

S29. ^{1}H NMR spectrum of derivative **11**

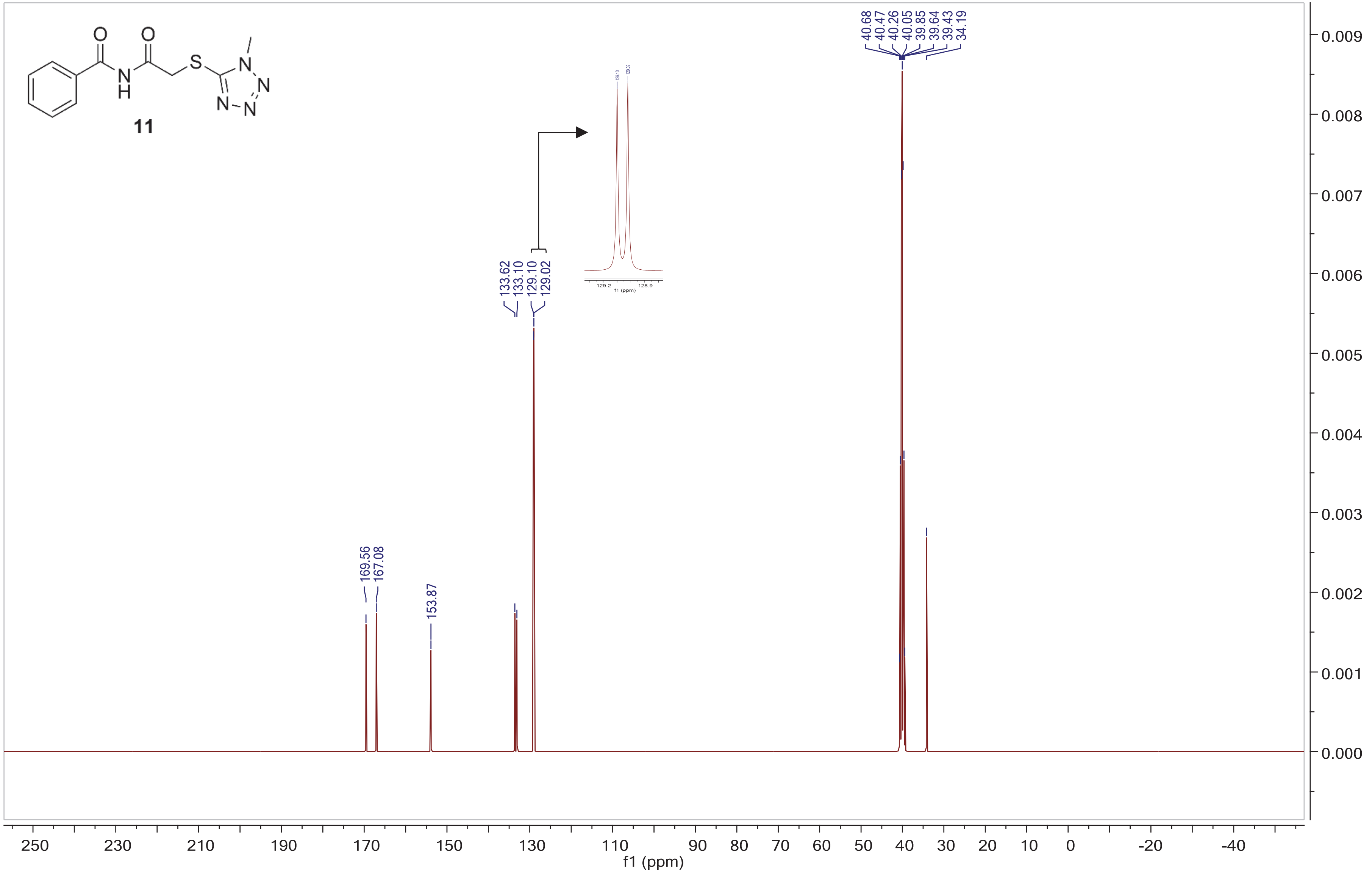

S30. ^{13}C NMR spectrum of derivative **11**

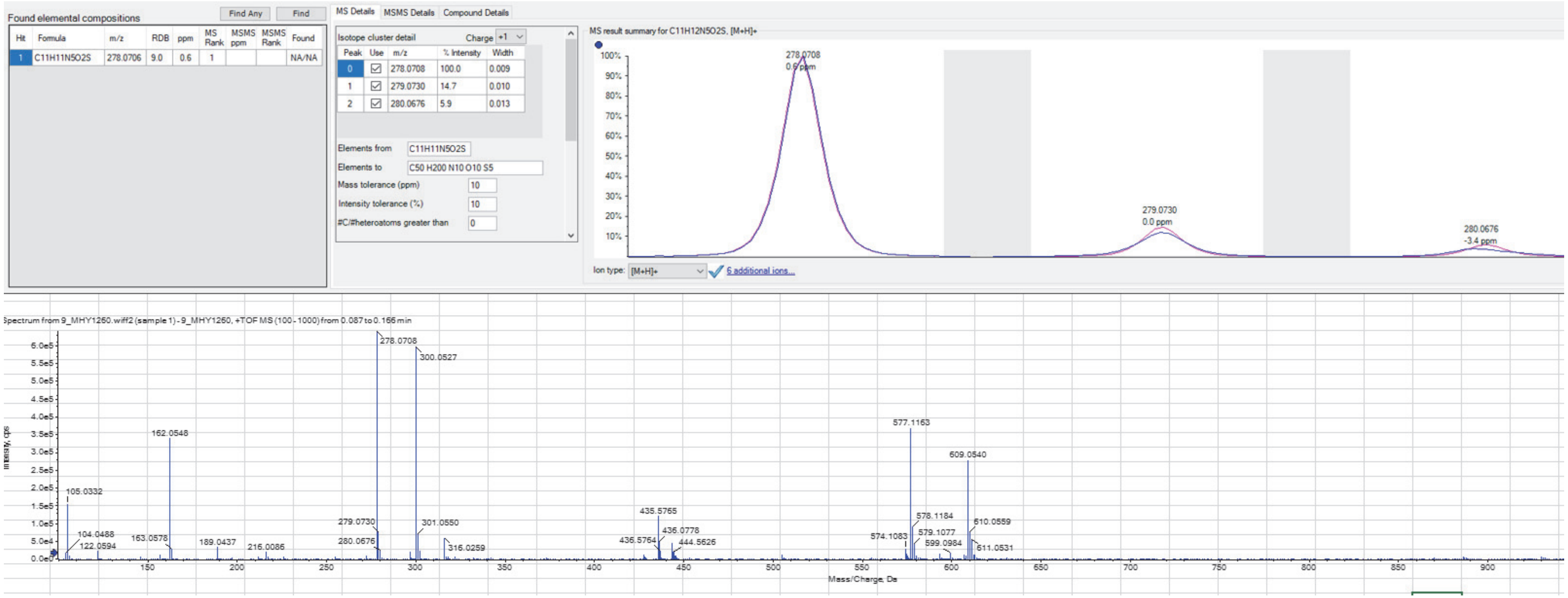

S31. HRMS spectrum of derivative **11**

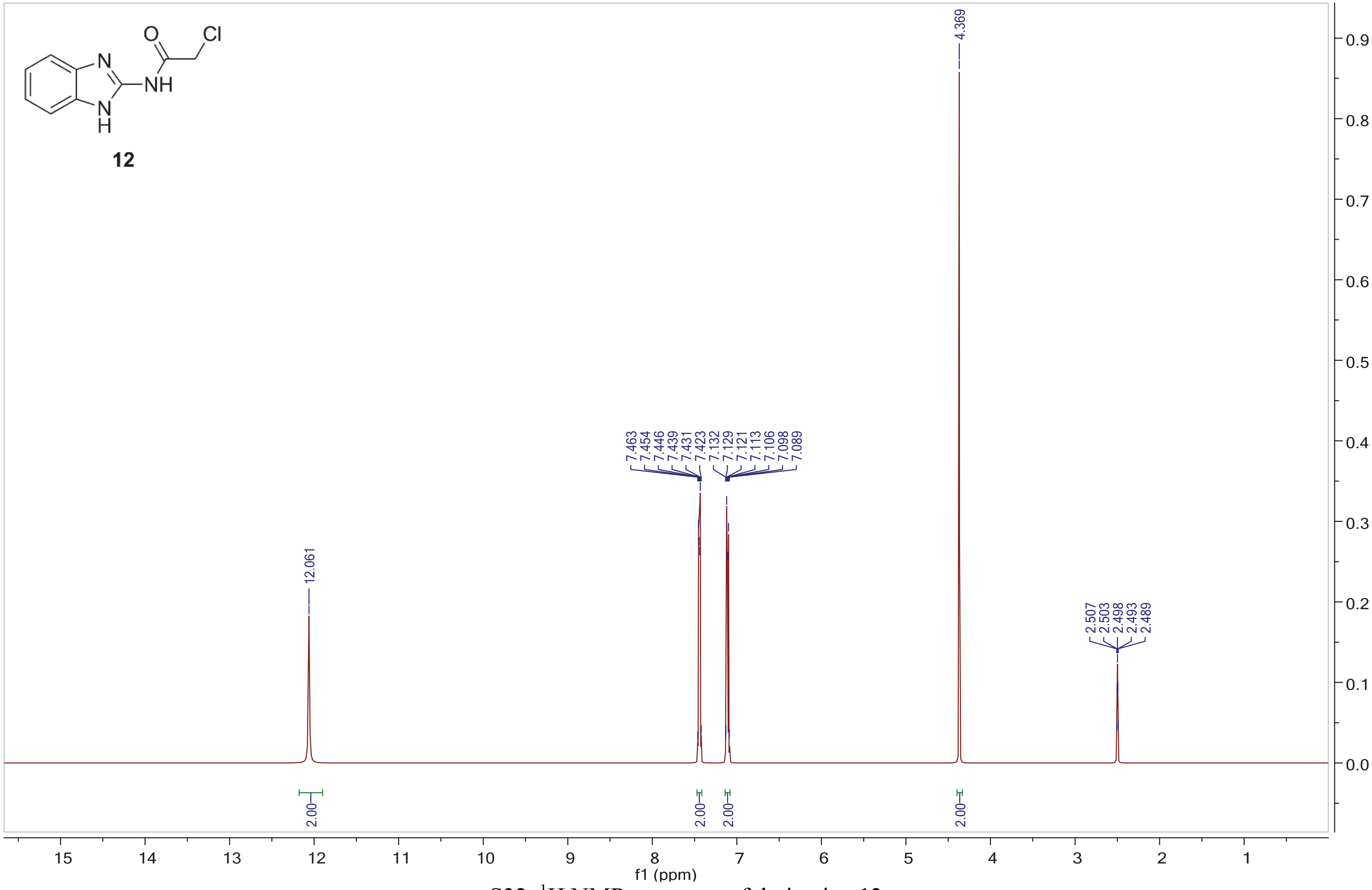

12

S32. ^{1}H NMR spectrum of derivative **12**

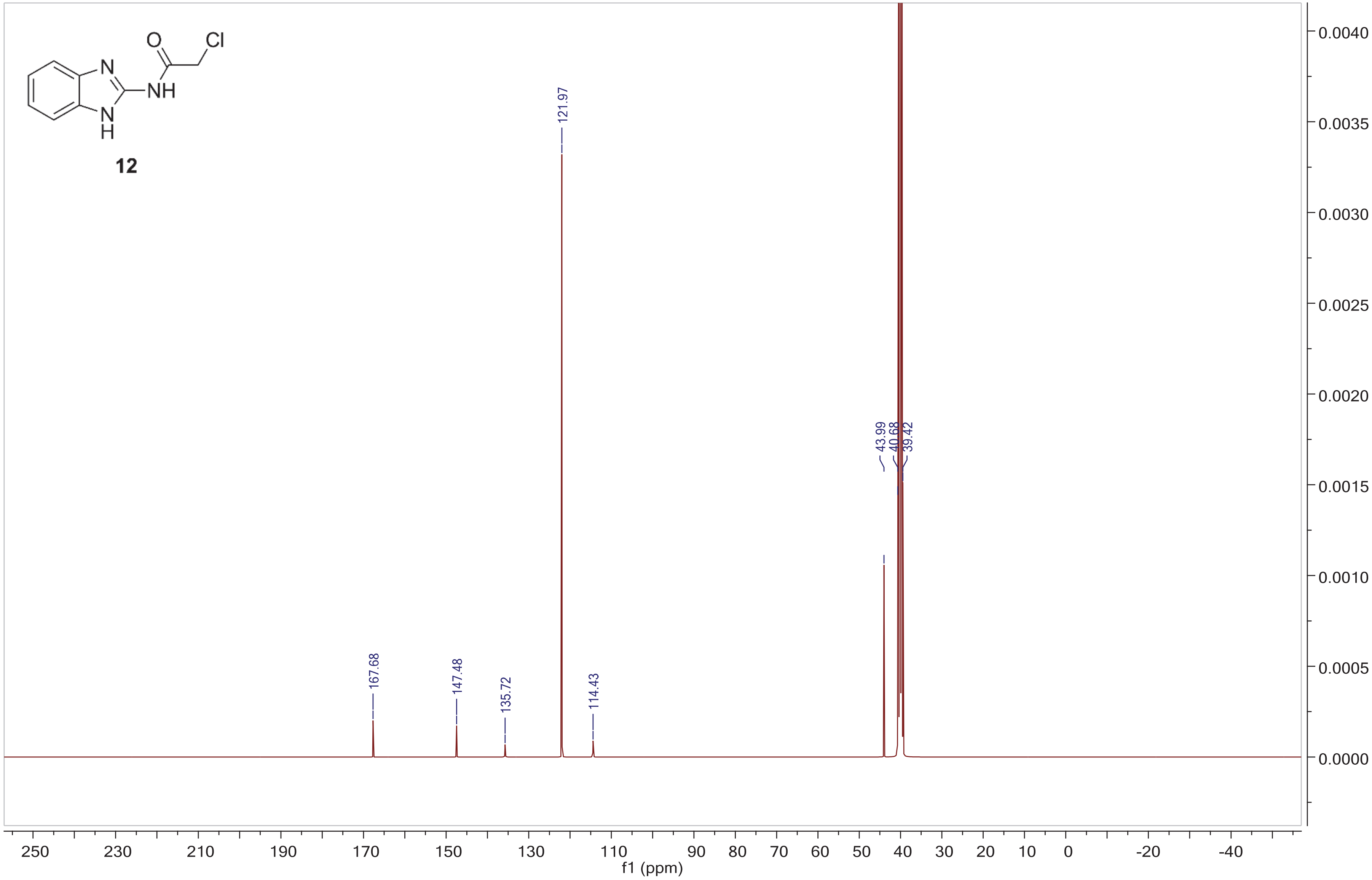

S33. ^{13}C NMR spectrum of derivative **12**

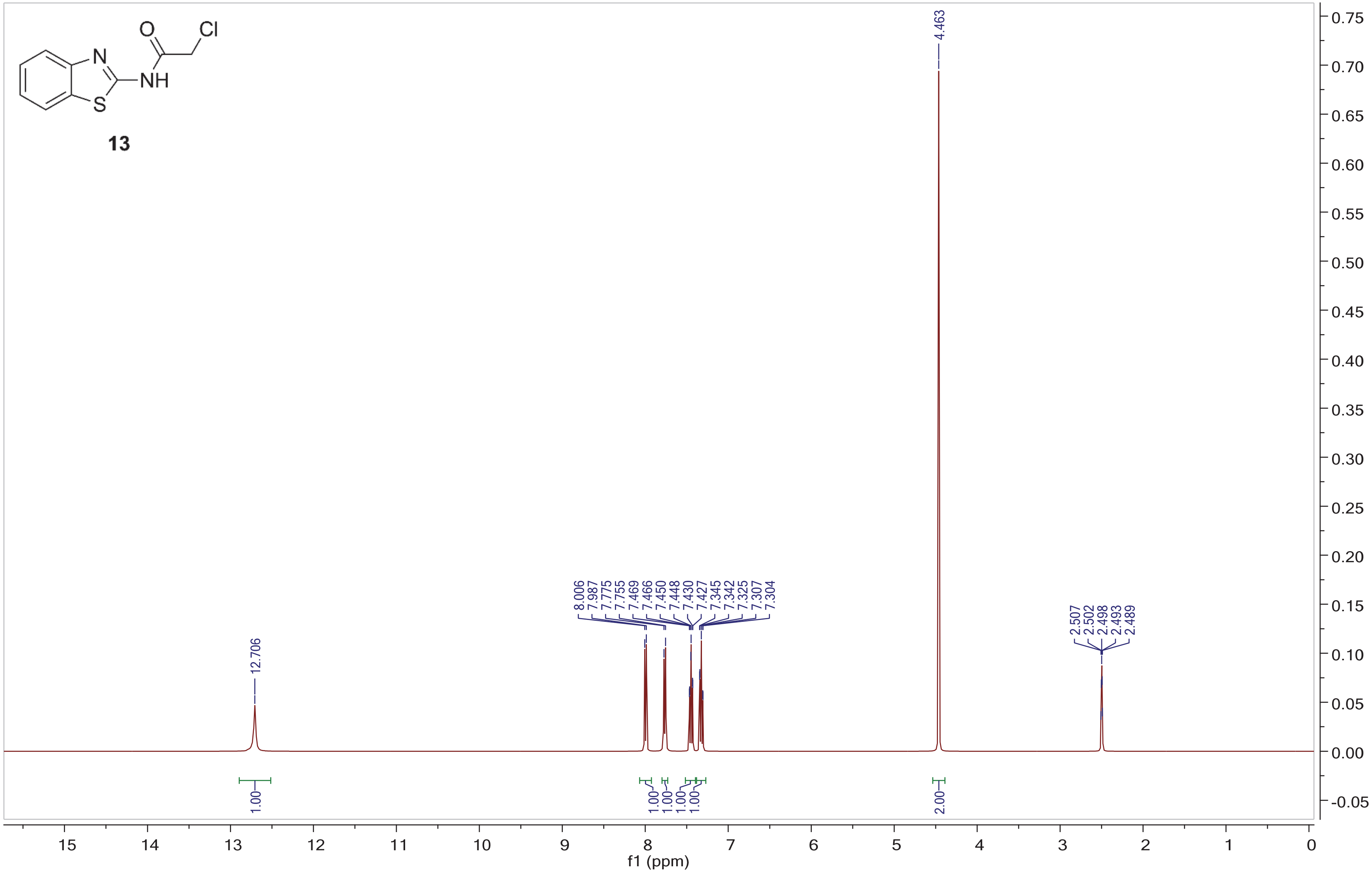

S34. ^{1}H NMR spectrum of derivative **13**

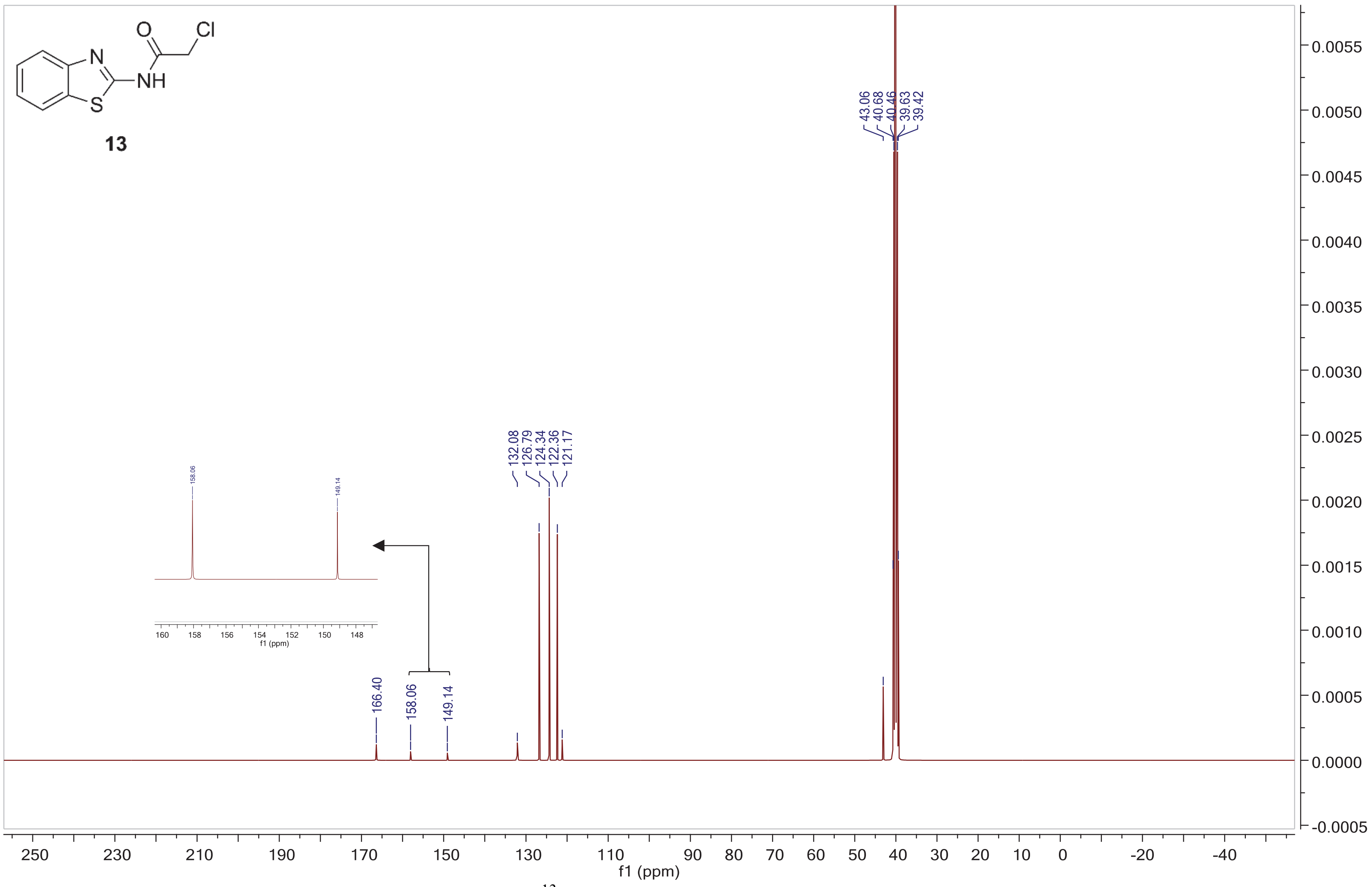

S35. ^{13}C NMR spectrum of derivative **13**

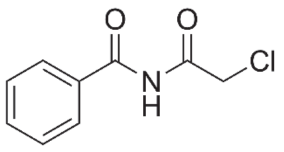

14

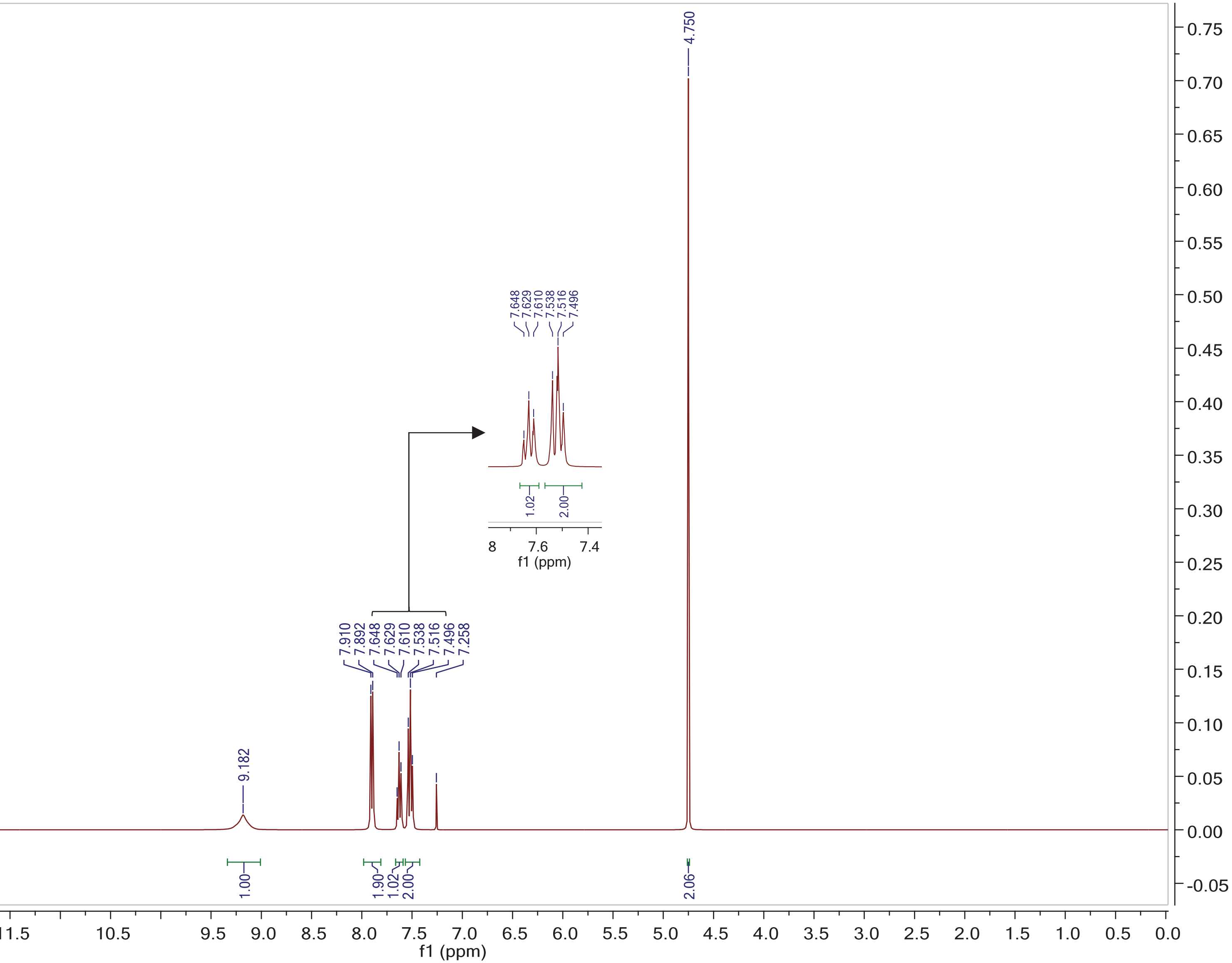

S36. ^{1}H NMR spectrum of derivative **14**

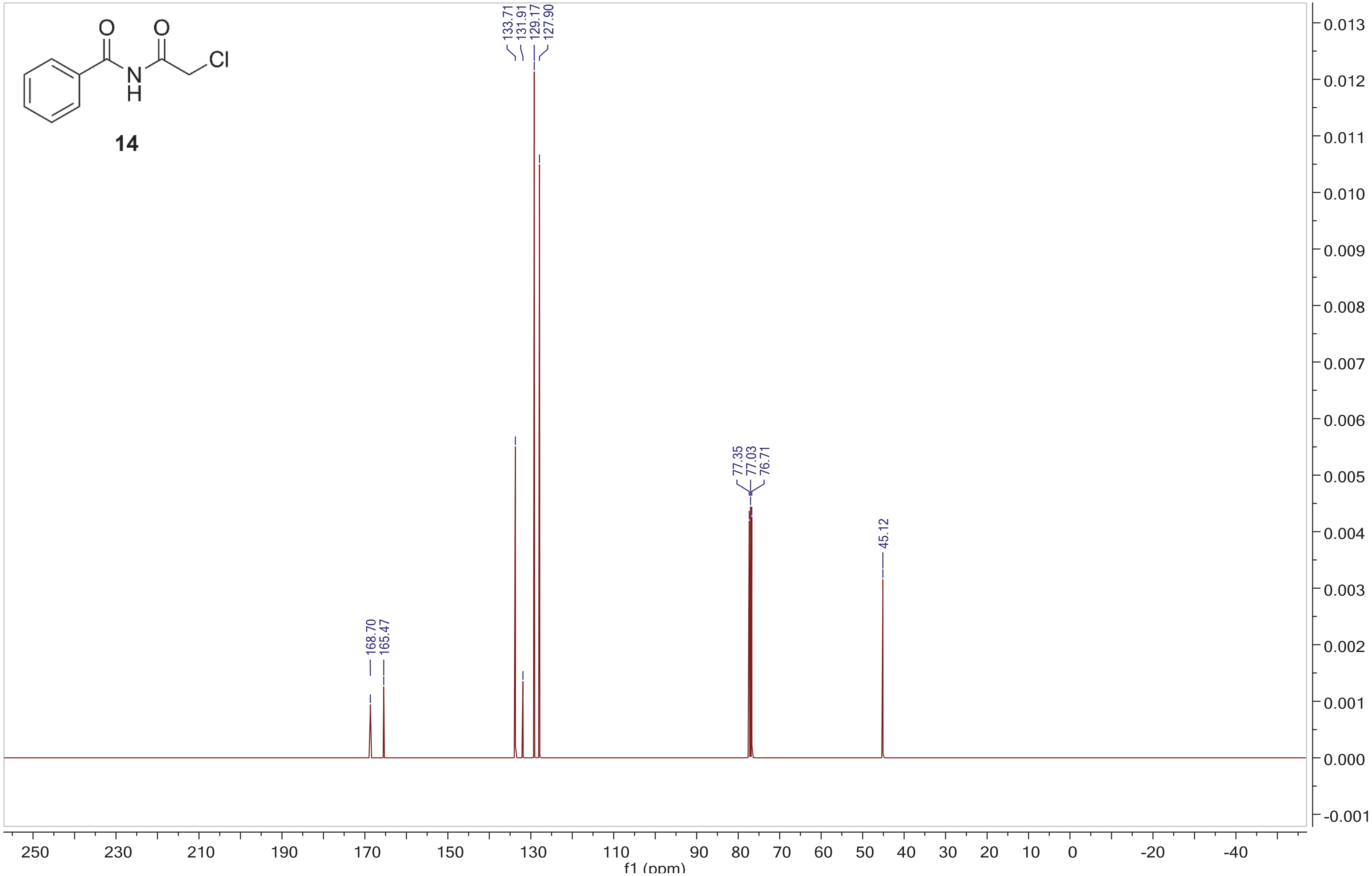

S37. ^{13}C NMR spectrum of derivative **14**

Substrate: L-Tyrosine

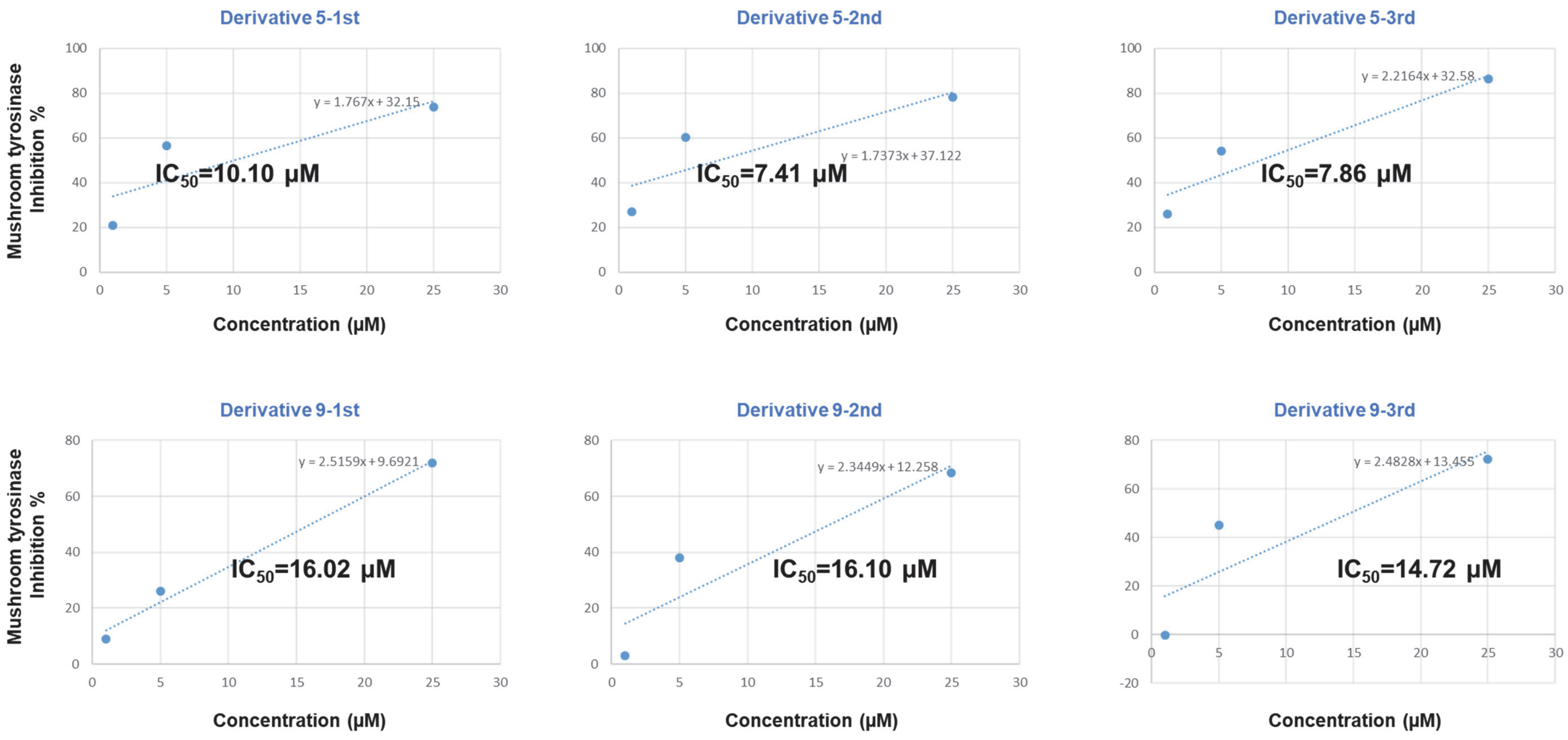

S38. Graphs used to calculate the IC_{50} values for derivatives **5** and **9** in the presence of L-tyrosine

Substrate: L-Tyrosine

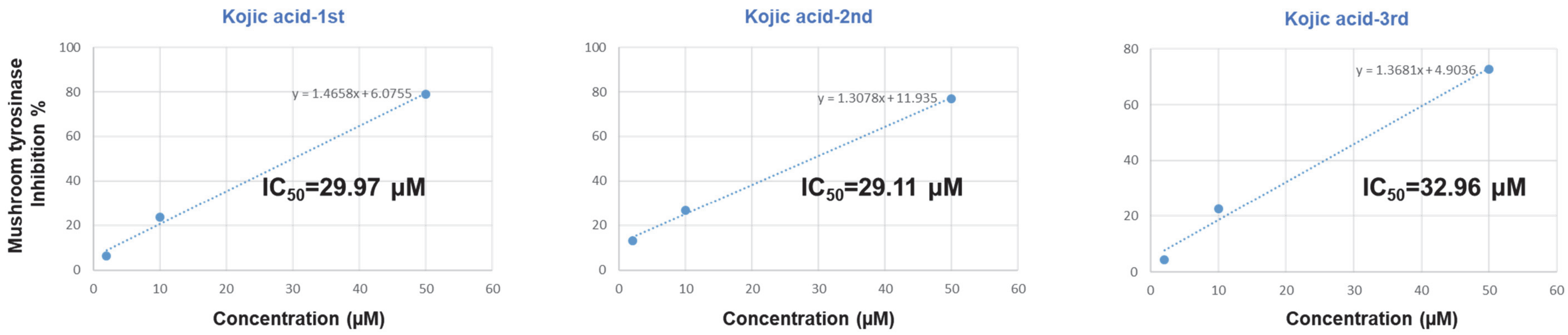

S39. Graphs used to calculate the IC_{50} value for kojic acid in the presence of L-tyrosine

Substrate: L-Dopa

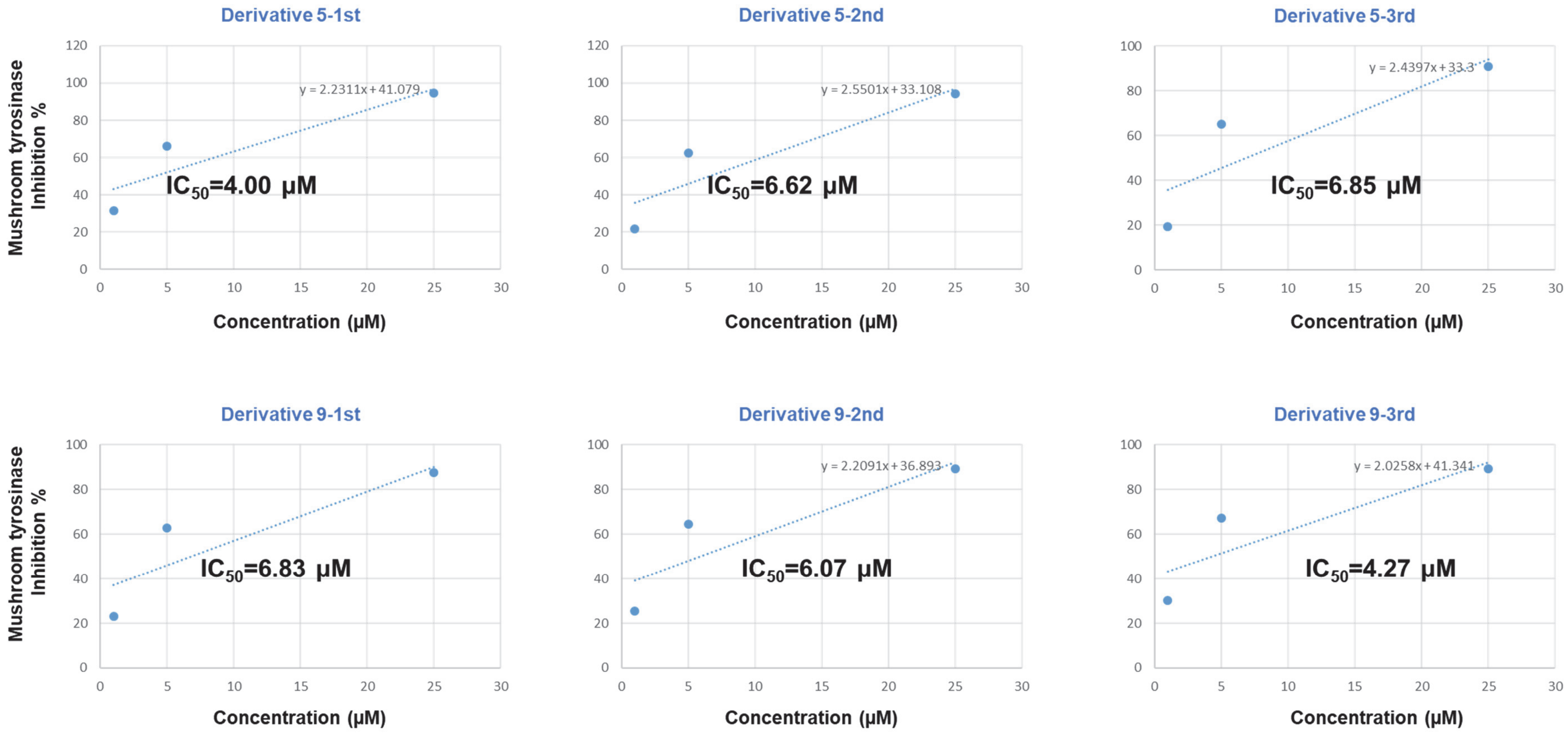

S40. Graphs used to calculate the IC_{50} values for derivatives **5** and **9** in the presence of L-dopa

Substrate: L-Dopa

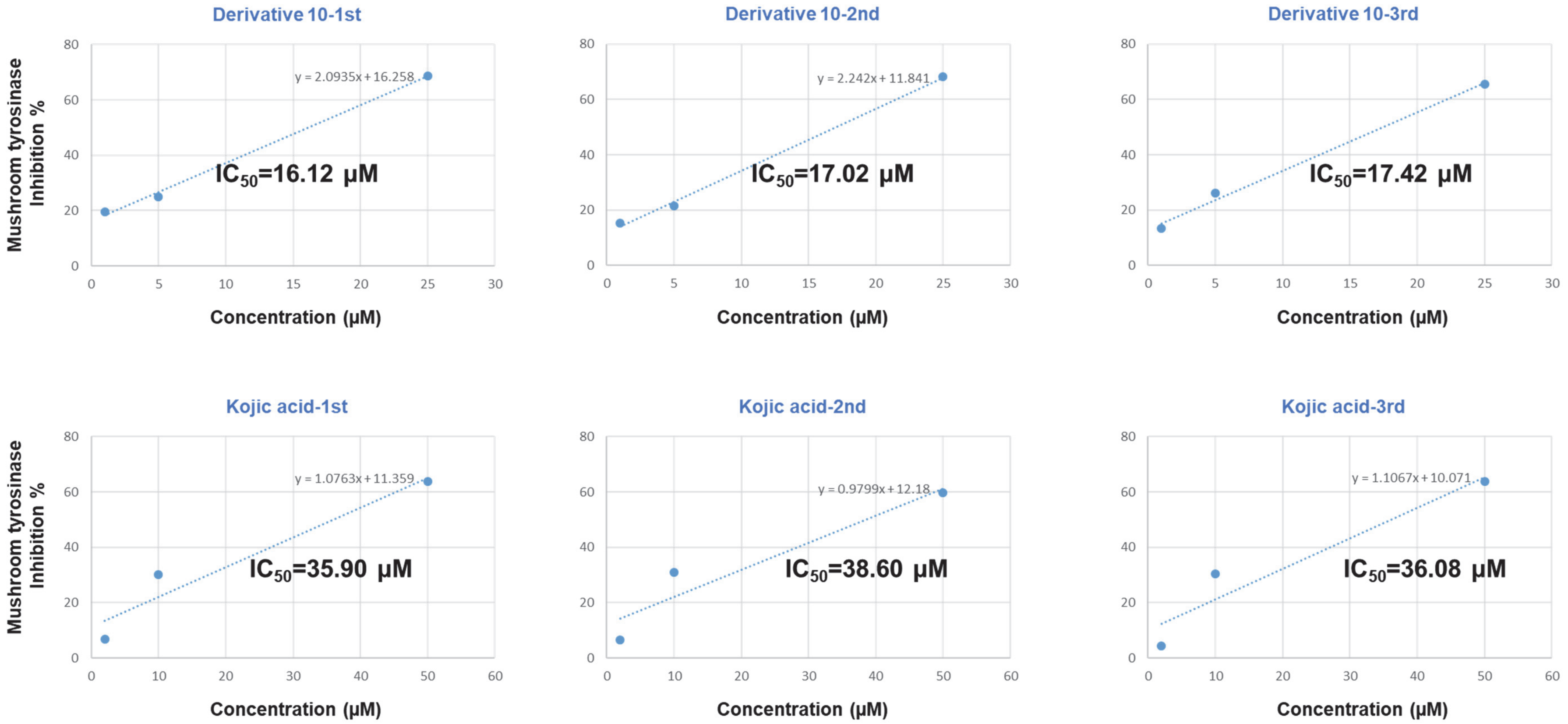

S41. Graphs used to calculate the IC_{50} values for derivative **10** and kojic acid in the presence of L-dopa

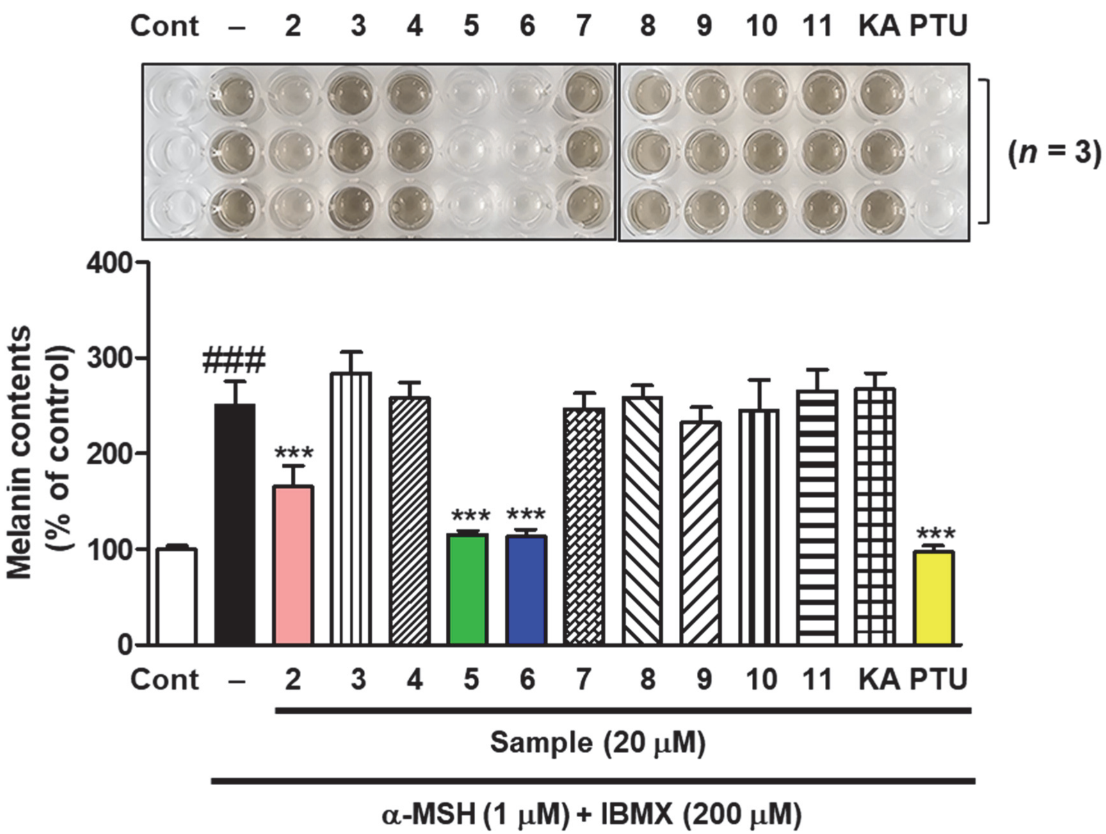

S42. Melanin content levels in the presence of AAMA derivatives **2**–**11** in B16F10 cells. Their melanin inhibition effects were compared using kojic acid (KA: 20 μM) and phenylthiourea (PTU)

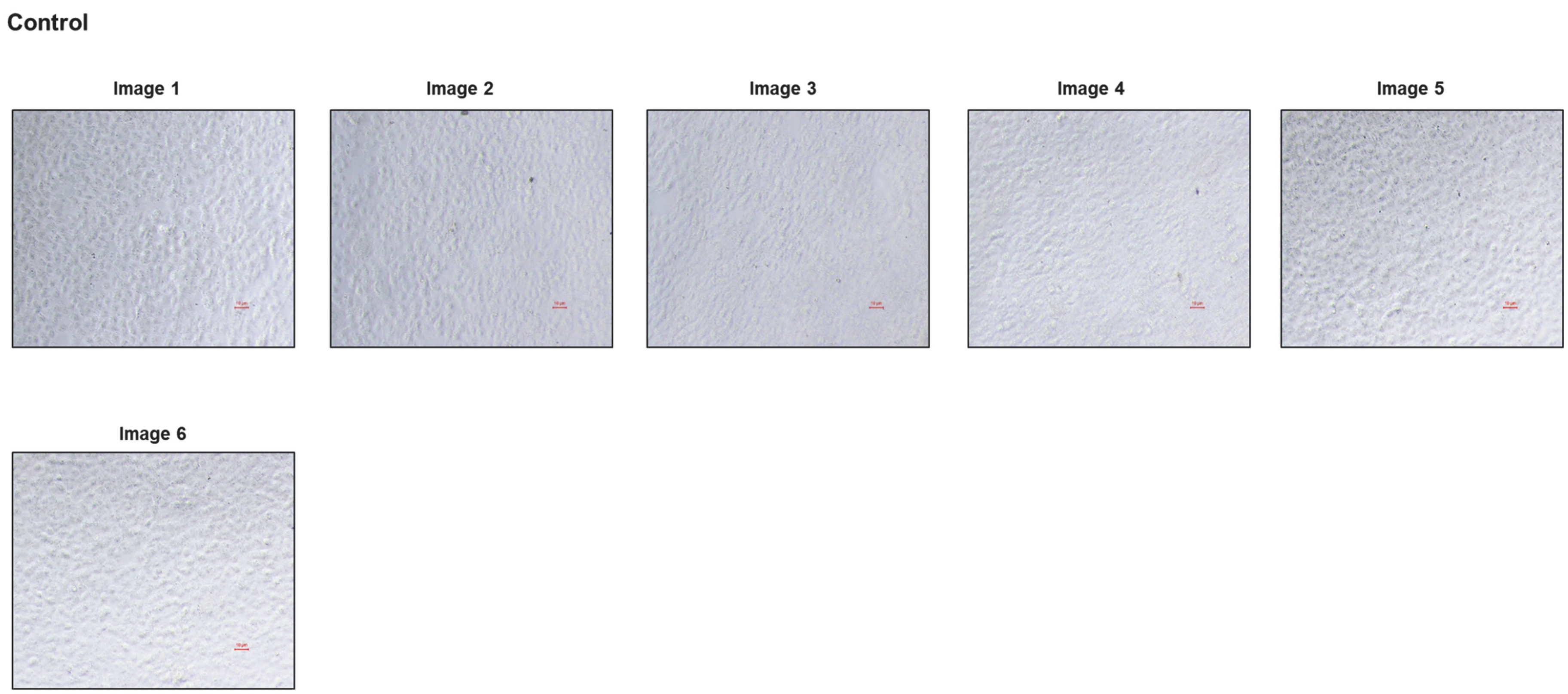

S43. Images of the control group (n = 6) in the in situ B16F10 cellular tyrosinase activity experiments

α-MSH + IBMX

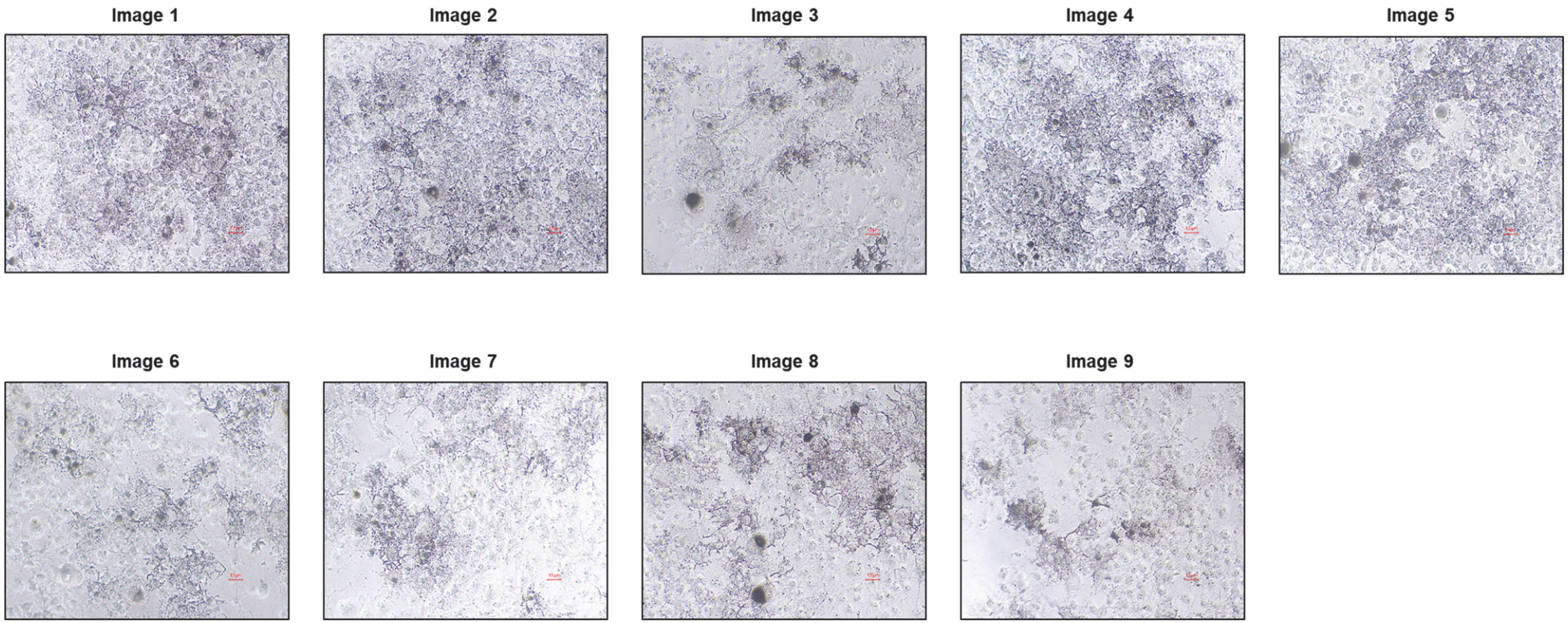

S44. Images of the α-MSH + IBMX group (*n* = 9) in the in situ B16F10 cellular tyrosinase activity experiments

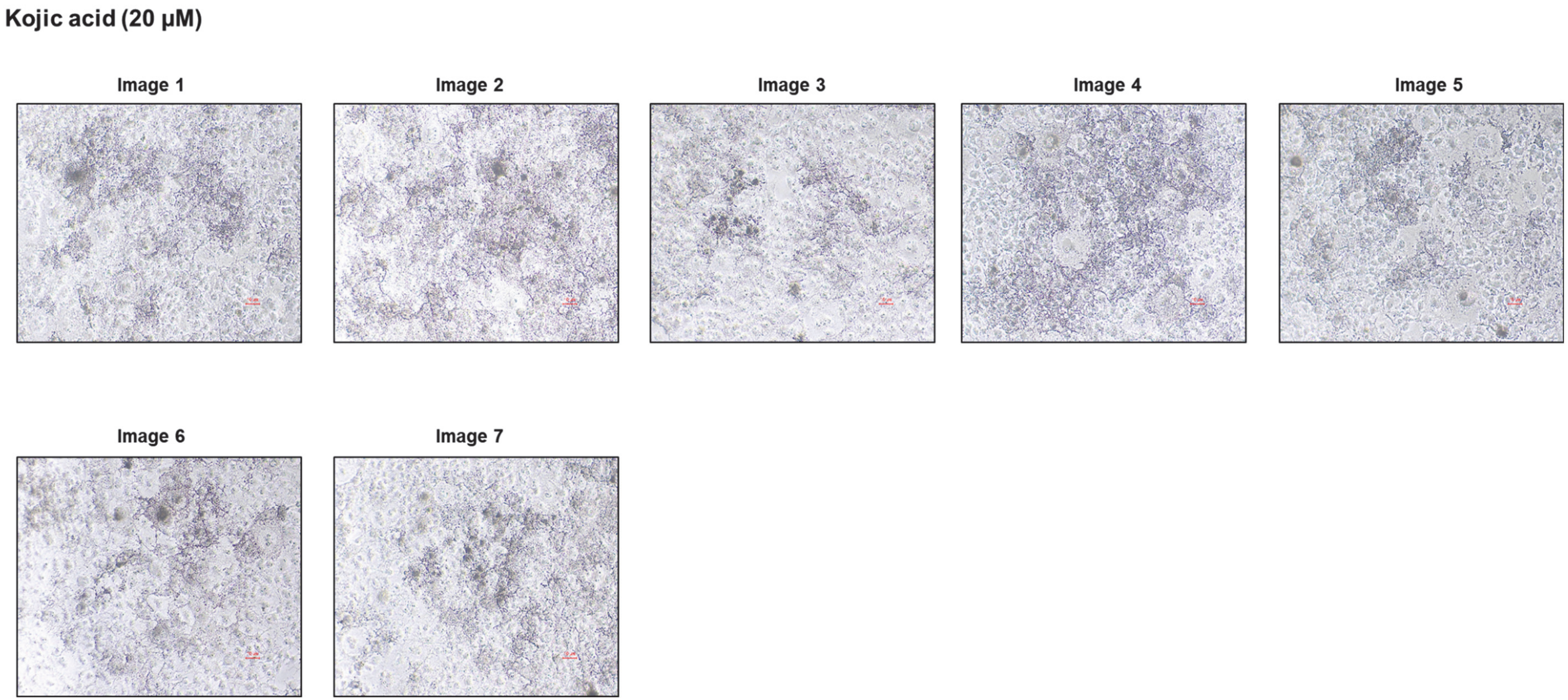

S45. Images of the kojic acid (20 μM) group (n = 7) in the in situ B16F10 cellular tyrosinase activity experiments

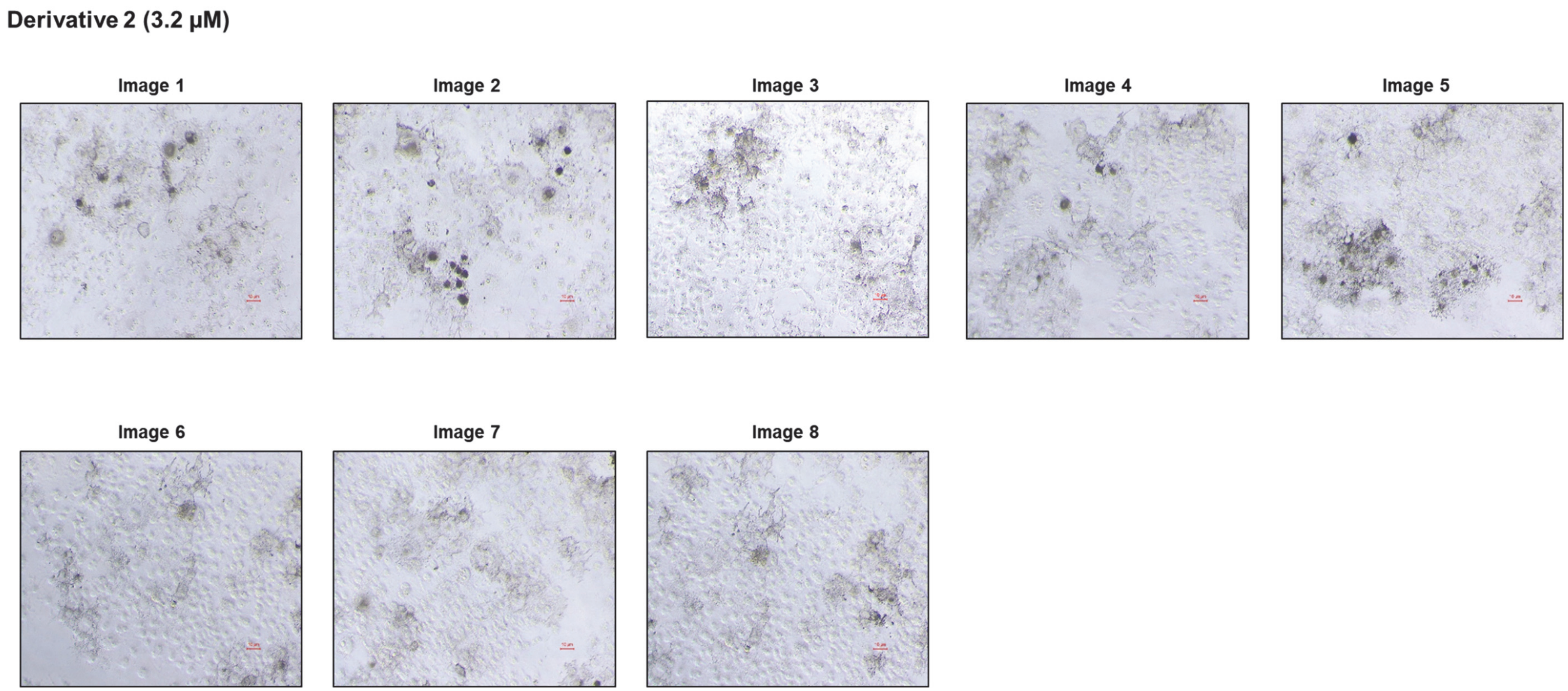

S46. Images of the derivative **2** (3.2 µM) group (n = 8) in the in situ B16F10 cellular tyrosinase activity experiments

Derivative 2 (8 μM)

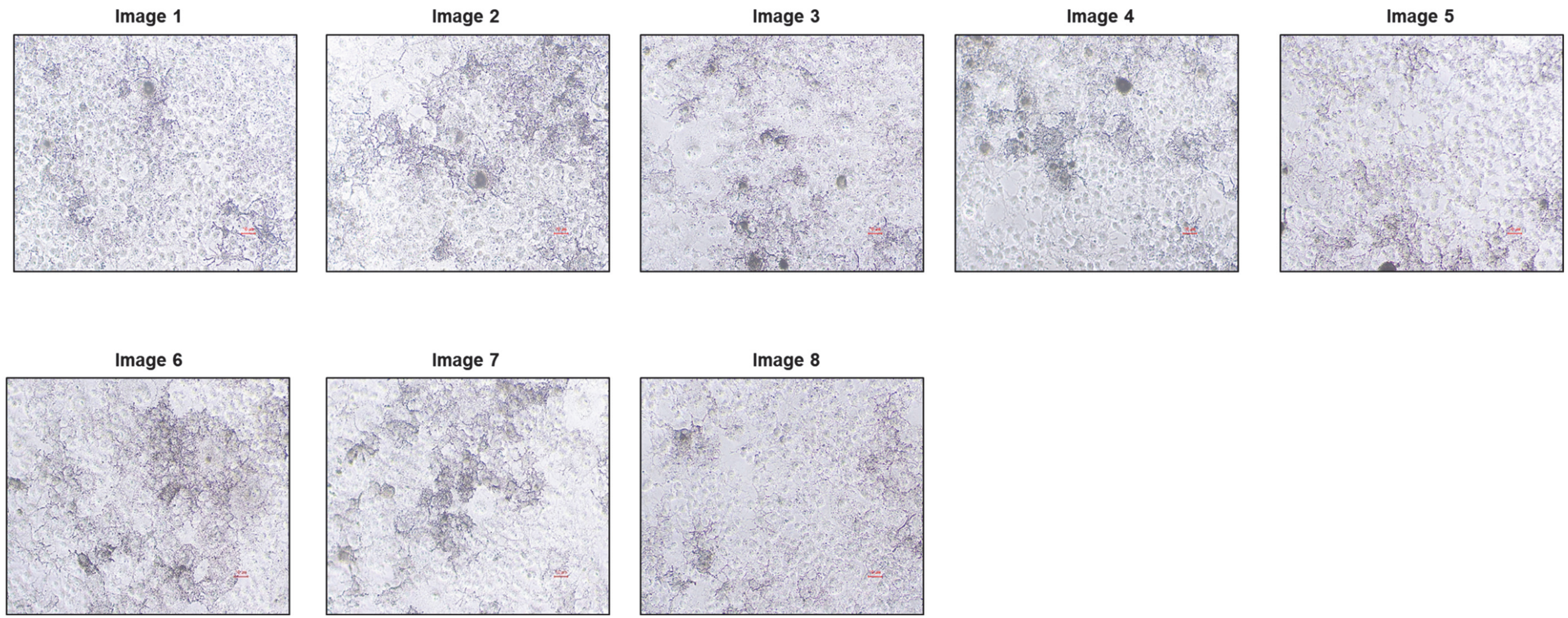

S47. Images of the derivative **2** (8 μM) group (n = 8) in the in situ B16F10 cellular tyrosinase activity experiments

Derivative 2 (20 μM)

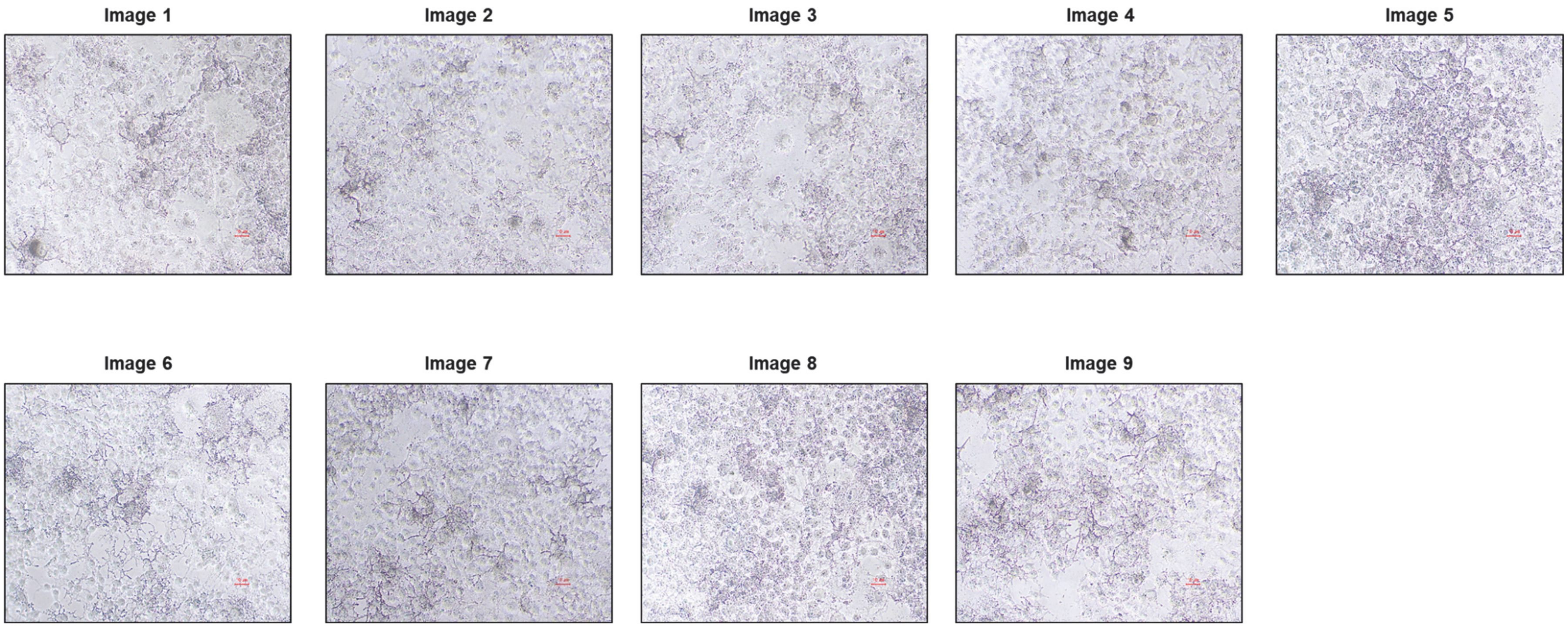

S48. Images of the the derivative **2** (20 μM) group (n = 9) in the in situ B16F10 cellular tyrosinase activity experiments

Derivative 5 (3.2 μM)

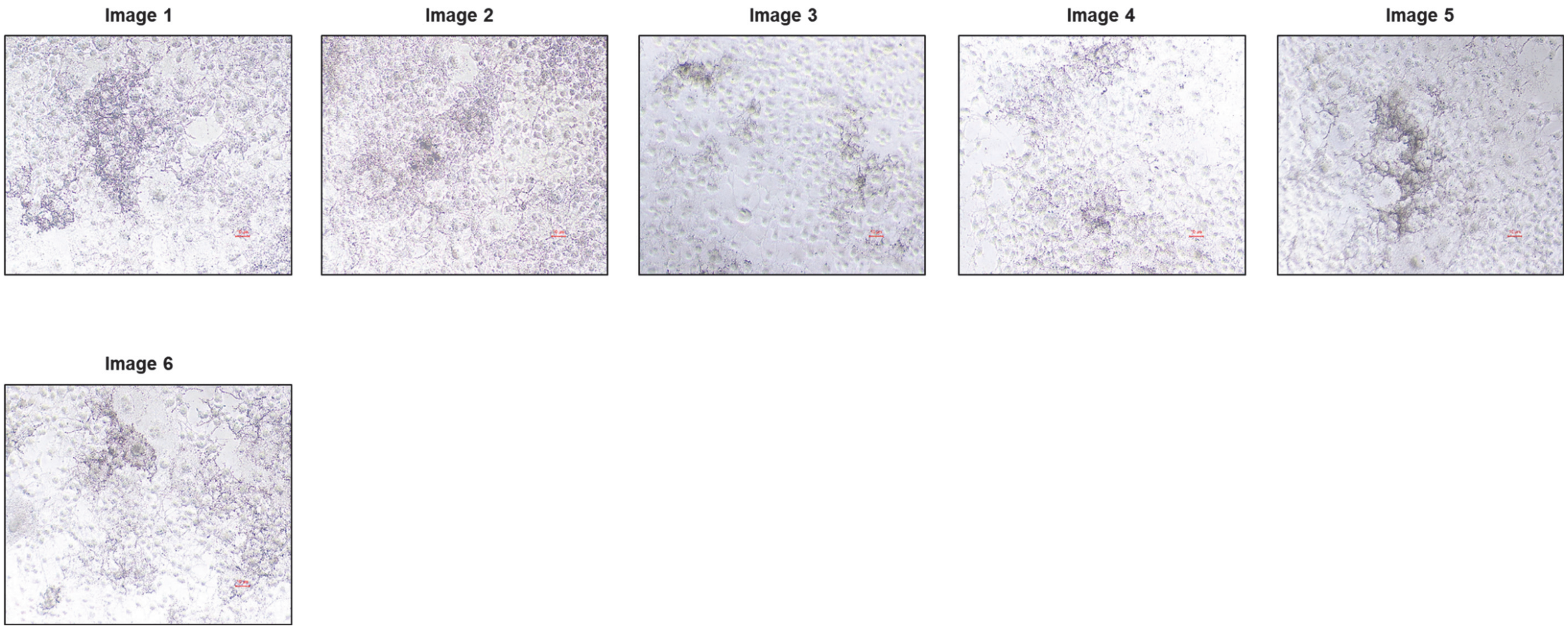

S49. Images of the derivative **5** (3.2 μM) group (n = 6) in the in situ B16F10 cellular tyrosinase activity experiments

Derivative 5 (8 μM)

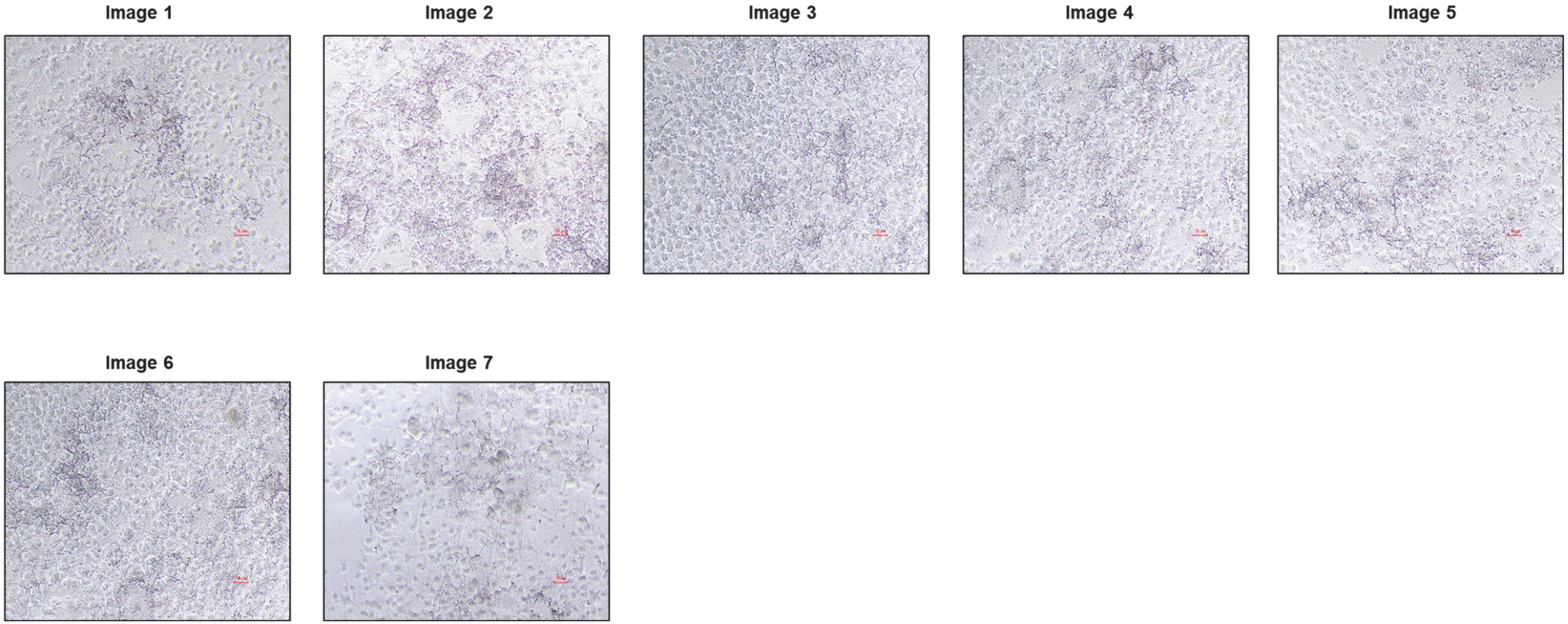

S50. Images of the derivative **5** (8 μM) group (n = 7) in the in situ B16F10 cellular tyrosinase activity experiments

Derivative 5 (20 µM)

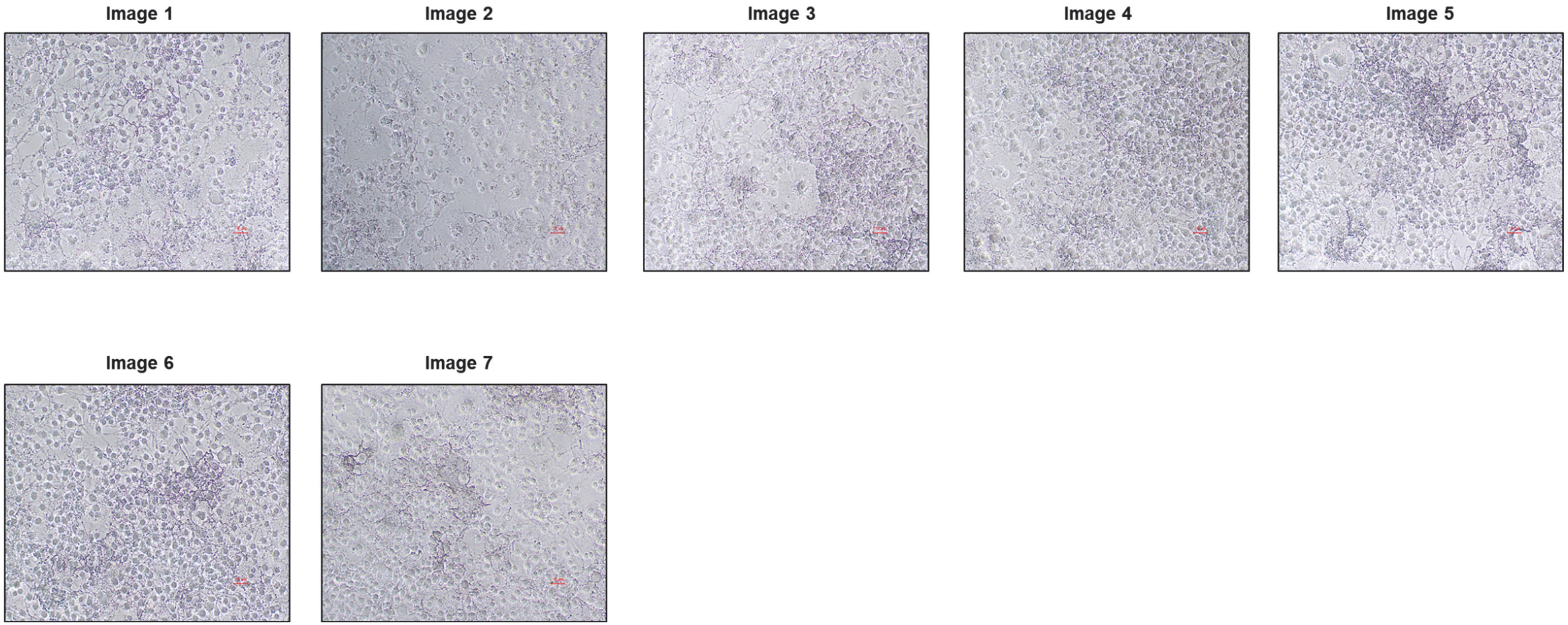

S51. Images of the the derivative **5** (20 µM) group (n = 7) in the in situ B16F10 cellular tyrosinase activity experiments

Derivative 6 (3.2 μM)

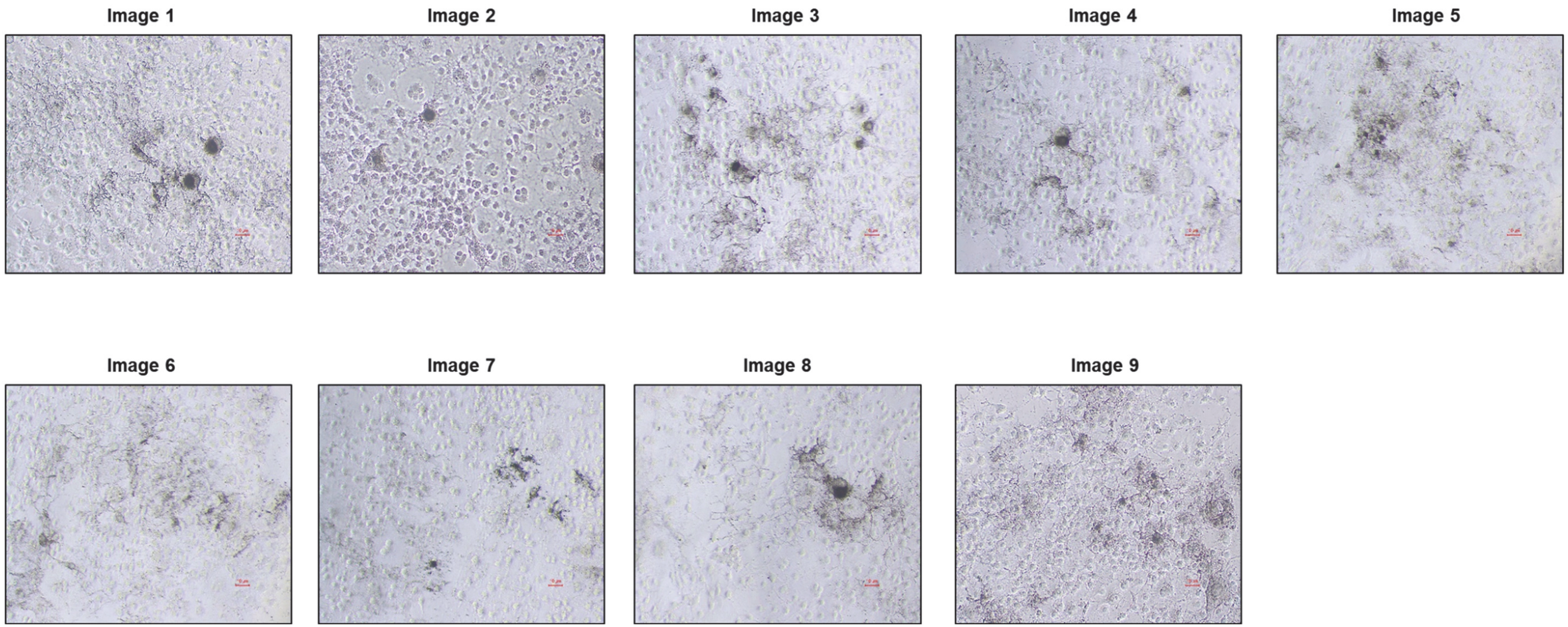

S52. Images of the derivative **6** (3.2 μM) group (n = 9) in the in situ B16F10 cellular tyrosinase activity experiments

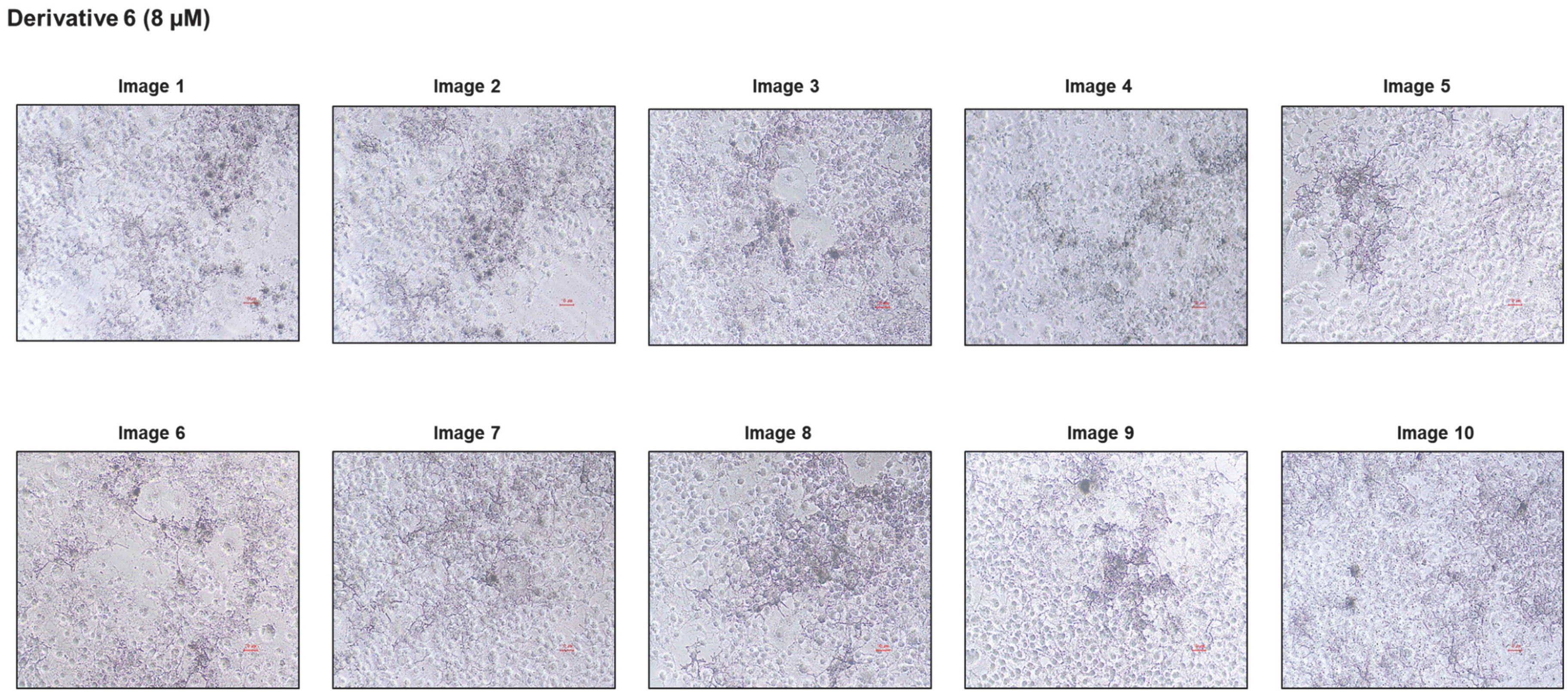

S53. Images of the derivative **6** (8 µM) group (*n* = 10) in the in situ B16F10 cellular tyrosinase activity experiments

Derivative 6 (20 μM)

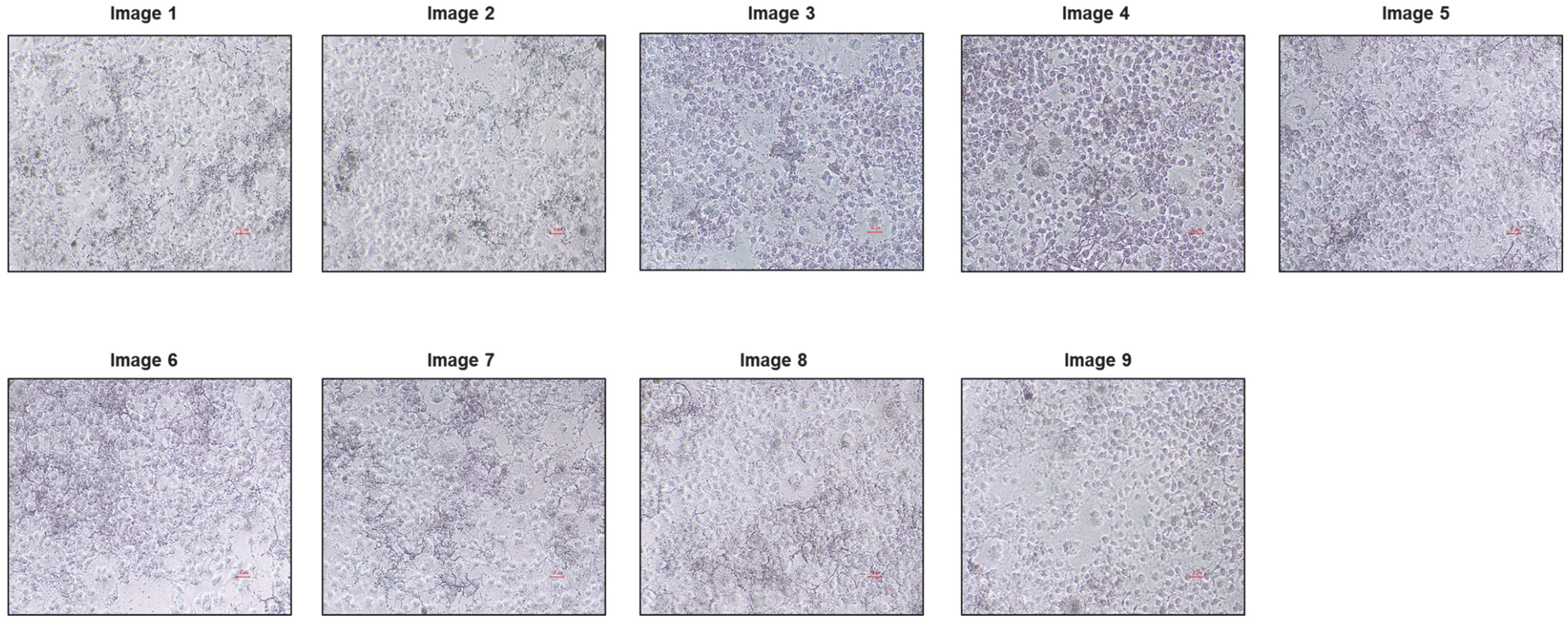

S54. Images of the the derivative **6** (20 μM) group (n = 9) in the in situ B16F10 cellular tyrosinase activity experiments

PTU (3.2 μM)

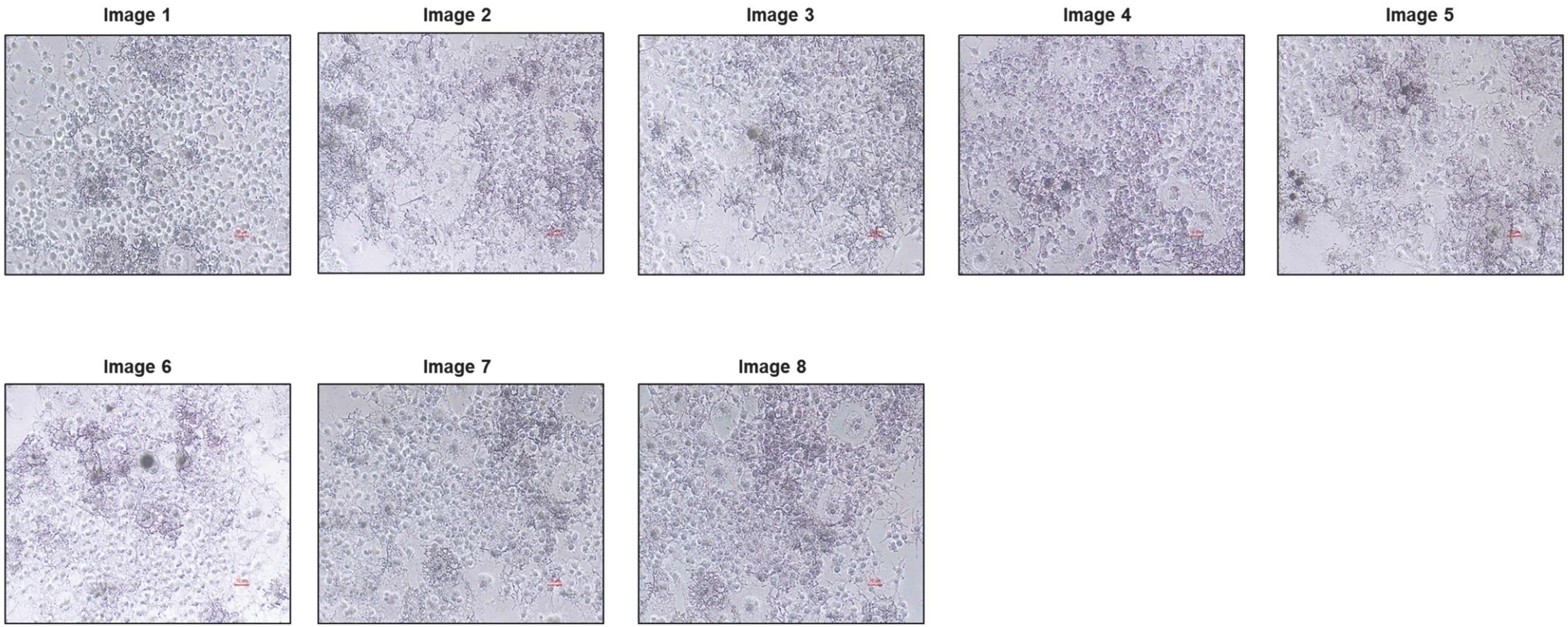

S55. Images of the PTU (3.2 μM) group (n = 8) in the in situ B16F10 cellular tyrosinase activity experiments

PTU (8 μM)

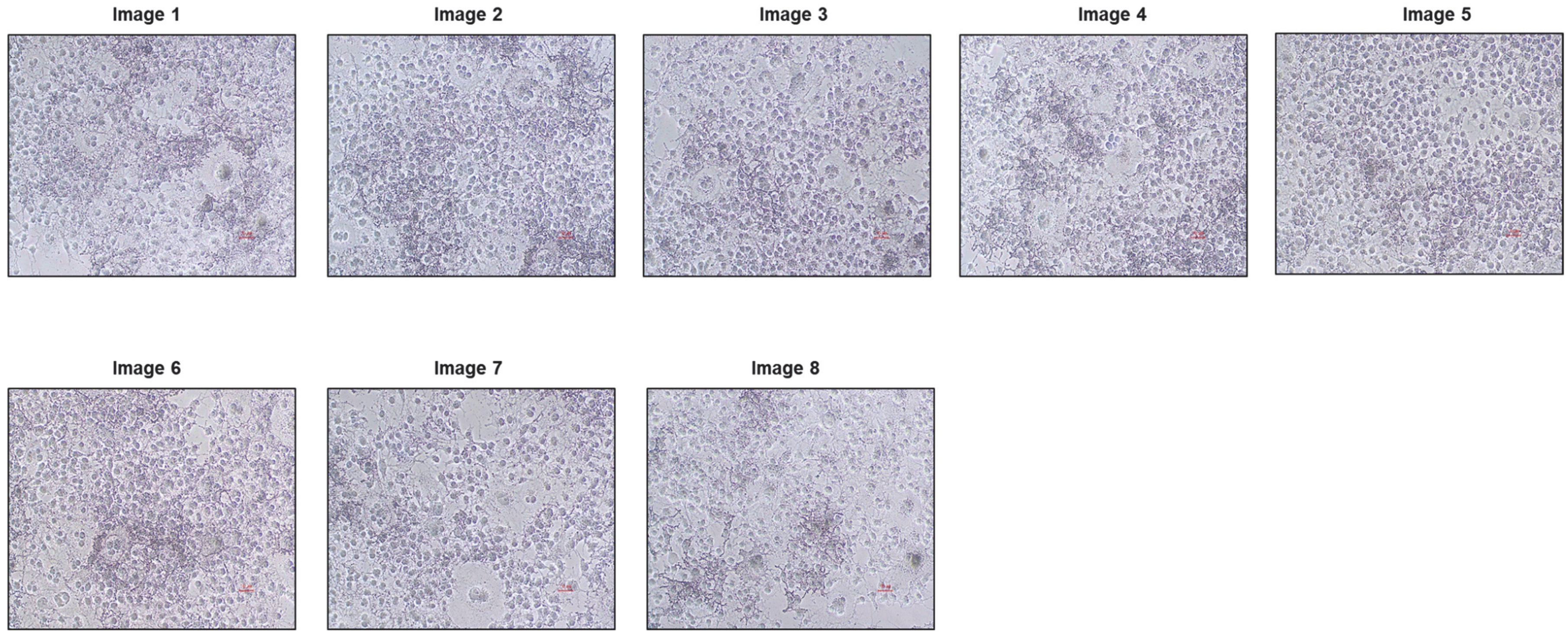

S56. Images of the PTU (8 μM) group (n = 8) in the in situ B16F10 cellular tyrosinase activity experiments

PTU (20 µM)

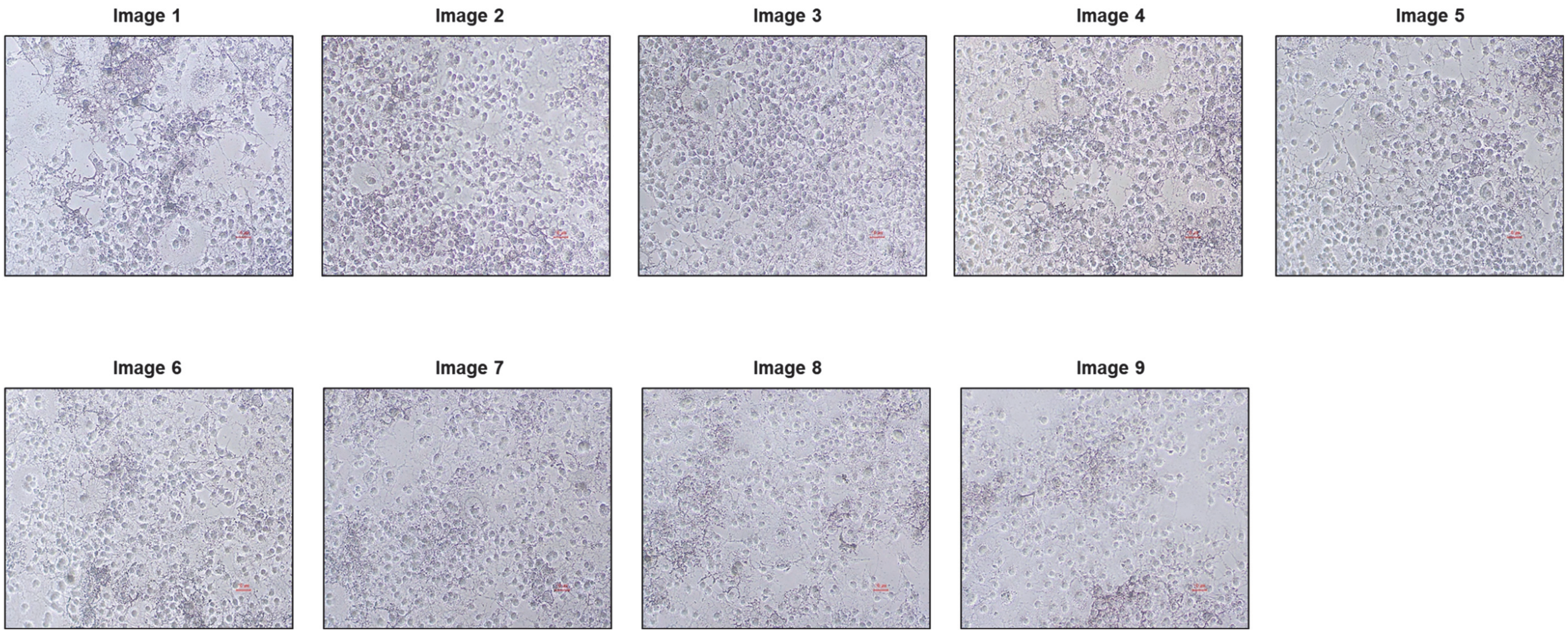

S57. Images of the the PTU (20 μM) group (n = 9) in the in situ B16F10 cellular tyrosinase activity experiments

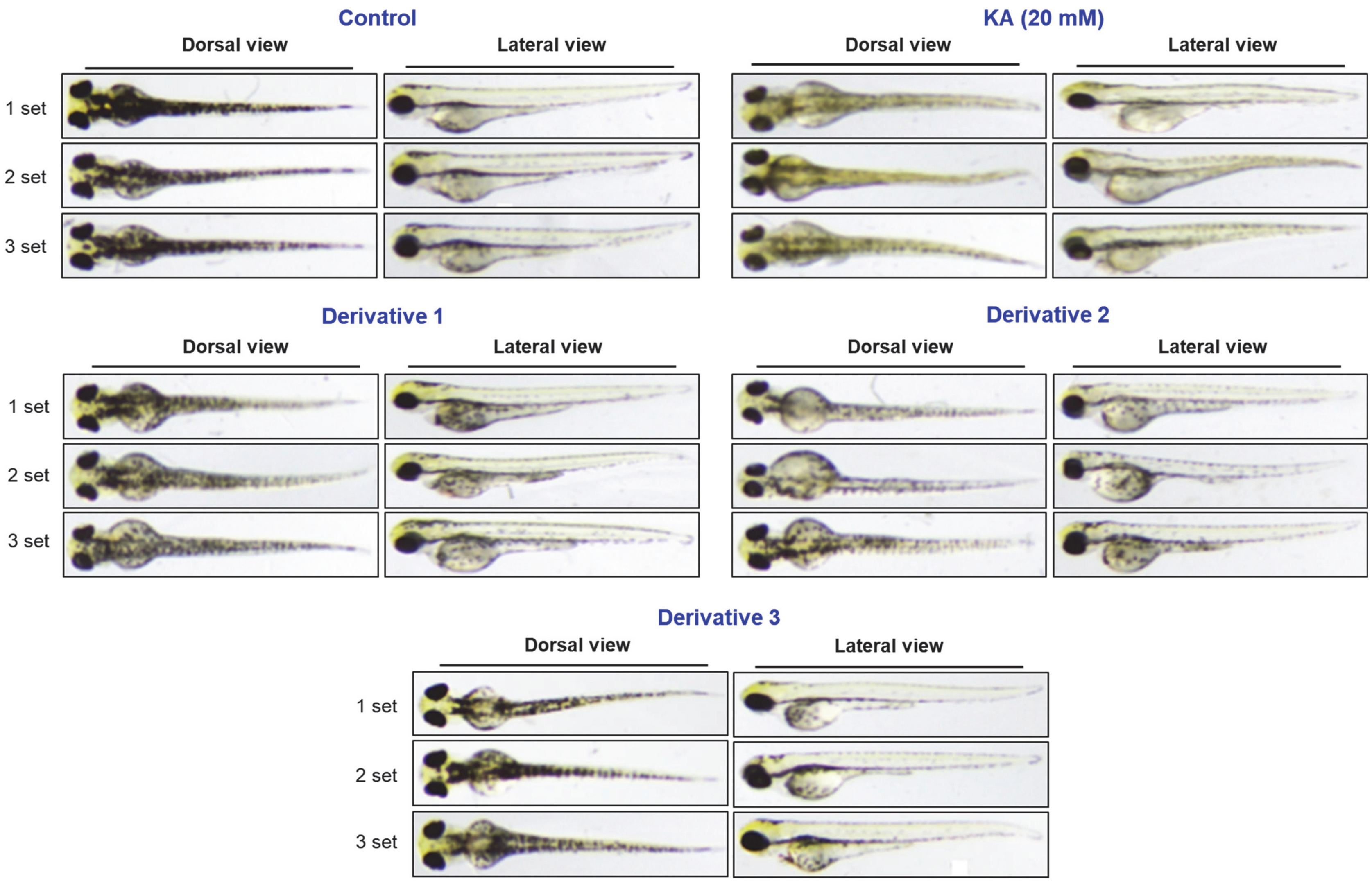

S58. Pigment-reducing effects of derivatives **1**–**3** and kojic acid in zebrafish larvae. Dorsal and lateral views of zebrafish larvae treated with control, KA (kojic acid, 20 mM), and derivatives **1**–**3** (0.1 mM).

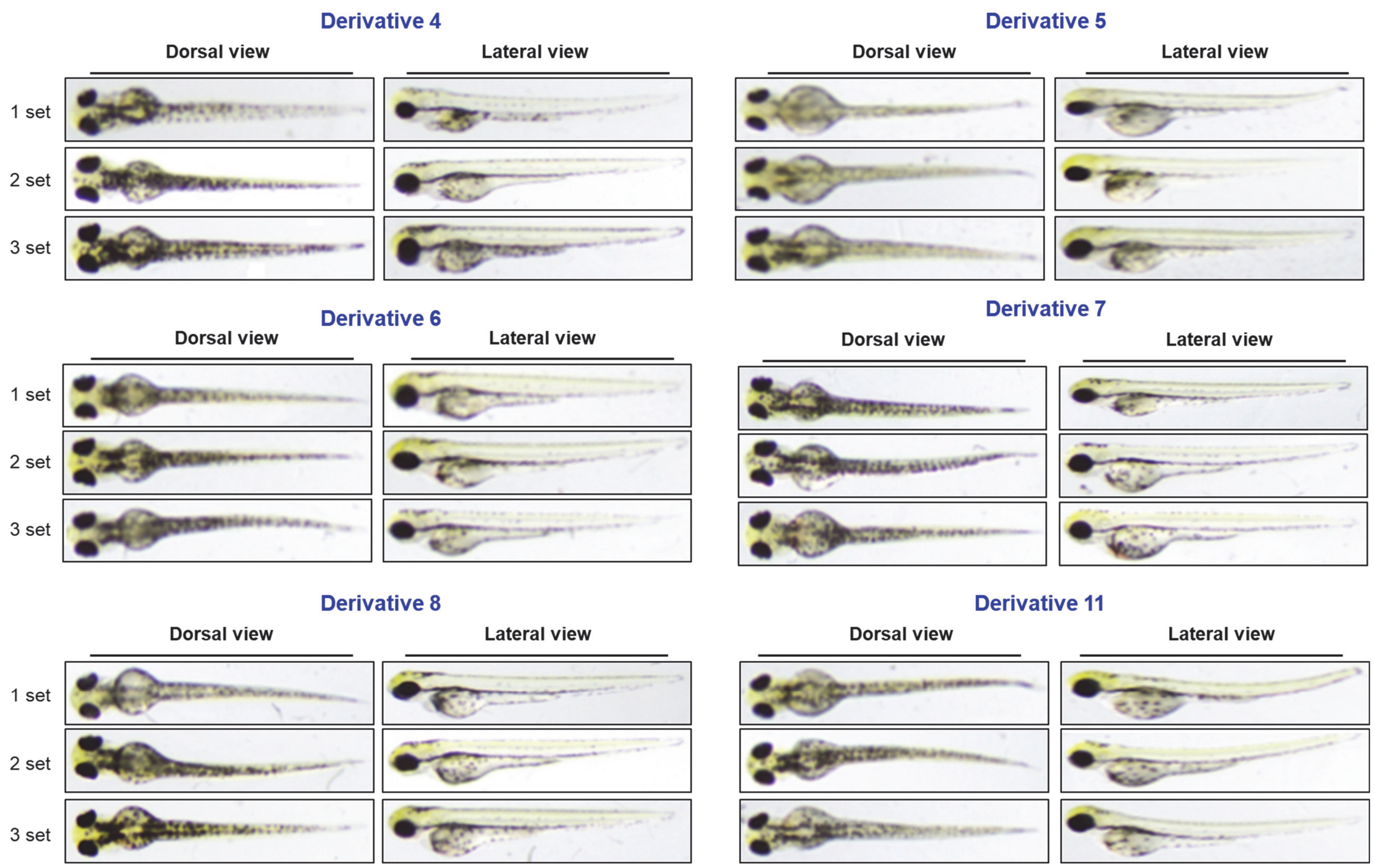

S59. Pigment-reducing effects of derivatives **4**–**11** in zebrafish larvae. Dorsal and lateral views of zebrafish larvae treated with derivatives **4**–**11** (0.1 mM). Images of derivatives **9** and **10** were not observed due to toxicity.

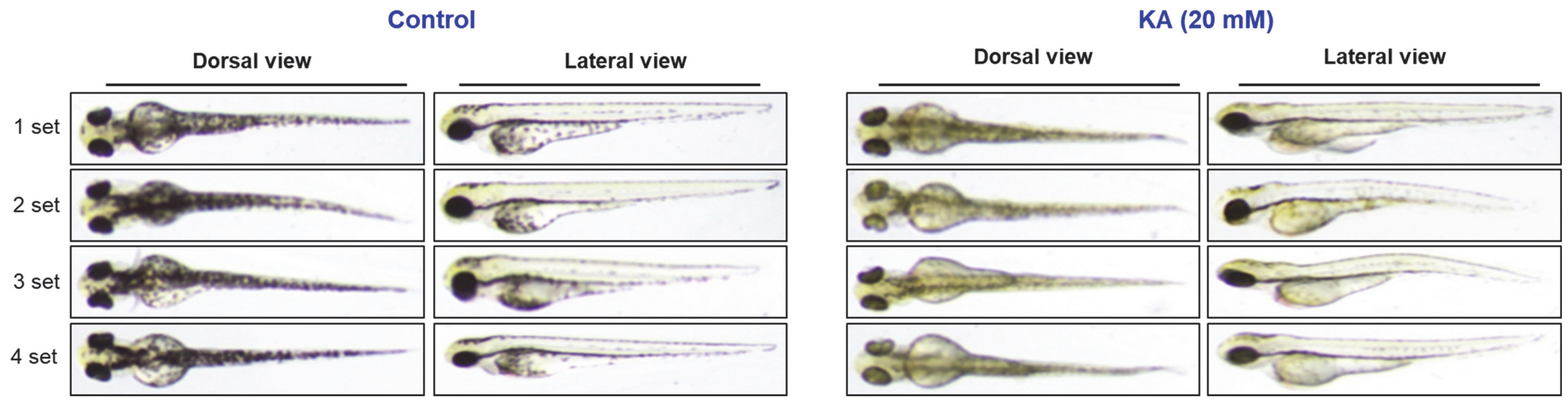

S60. Pigment-reducing effect of kojic acid in zebrafish larvae. Dorsal and lateral views of zebrafish larvae treated with control and KA (kojic acid, 20 mM).

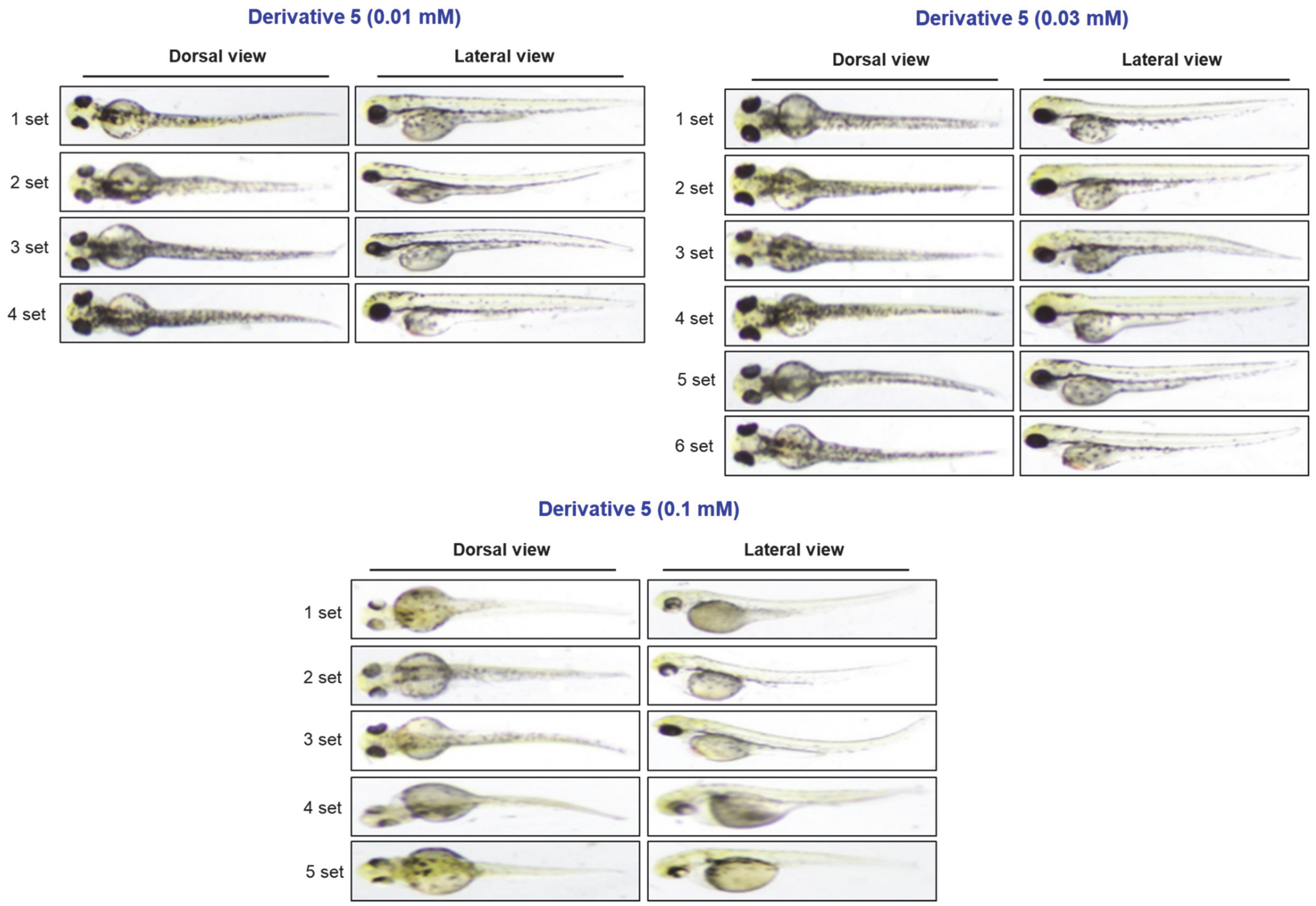

S61. Pigment-reducing effect of derivative **5** in zebrafish larvae. Dorsal and lateral views of zebrafish larvae treated with derivative **5** (0.01, 0.03, and 0.1 mM).

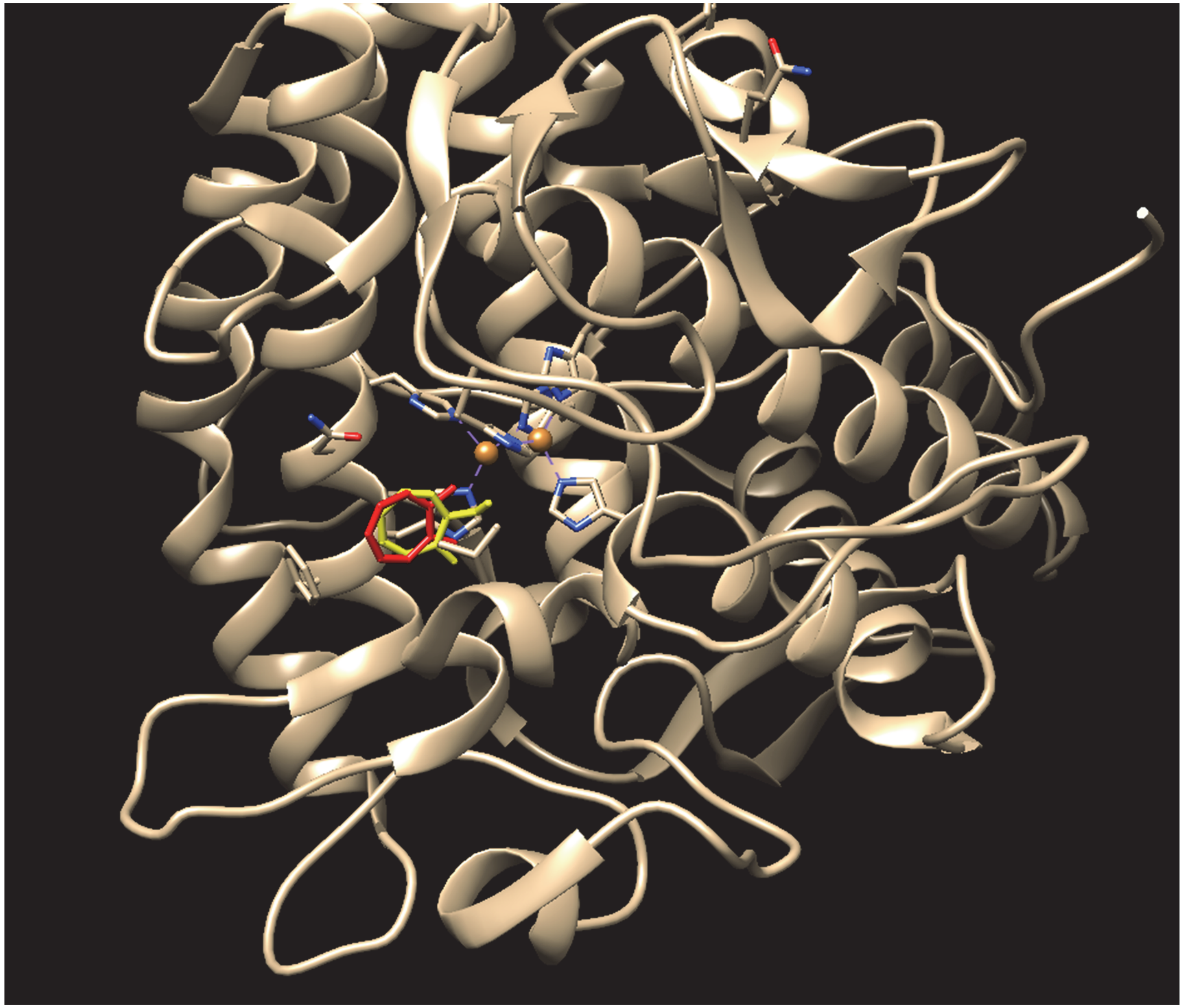

S62. Alignment of the redocked ligand (yellow; tropolone) and co-crystallized ligand (red: tropolone) with the 2Y9X protein.

In the redocking results, it was found that the redocked tropolone reproduced the binding pose with a binding affinity of −5.5 kcal/mol.

Table Analyzed	Copper				
One-way analysis of variance					
P value	< 0.0001				
P value summary	***				
Are means signif. different? (P < 0.05)	Yes				
Number of groups	14				
F	92.55				
R square	0.9773				
ANOVA Table	SS	df	MS		
Treatment (between columns)	15503	13	1193		
Residual (within columns)	360.8	28	12.89		
Total	15864	41			
Bonferroni's Multiple Comparison Test	Mean Diff.	t	Significant? P < 0.05?	Summary	95% CI of diff
Control vs deriv. **1**	-7.554	2.594	No	ns	-18.91 to 3.804
Control vs deriv. **2**	-13.37	4.590	Yes	**	-24.73 to -2.011
Control vs deriv. **3**	-52.90	18.16	Yes	***	-64.26 to -41.54
Control vs deriv. **4**	-35.71	12.26	Yes	***	-47.07 to -24.35
Control vs deriv. **5**	-11.15	3.827	No	ns	-22.50 to 0.2126
Control vs deriv. **6**	-2.303	0.7908	No	ns	-13.66 to 9.055
Control vs deriv. **7**	-53.60	18.40	Yes	***	-64.96 to -42.24
Control vs deriv. **8**	-34.85	11.97	Yes	***	-46.21 to -23.50
Control vs deriv. **9**	-4.019	1.380	No	ns	-15.38 to 7.340
Control vs deriv. **10**	-2.649	0.9095	No	ns	-14.01 to 8.709
Control vs deriv. **11**	-0.3528	0.1211	No	ns	-11.71 to 11.01
Control vs KA	-31.88	10.94	Yes	***	-43.23 to -20.52
Control vs PTU	-38.87	13.34	Yes	***	-50.23 to -27.51
deriv. **1** vs deriv. **2**	-5.815	1.997	No	ns	-17.17 to 5.543
deriv. **1** vs deriv. **3**	-45.34	15.57	Yes	***	-56.70 to -33.98
deriv. **1** vs deriv. **4**	-28.16	9.668	Yes	***	-39.52 to -16.80
deriv. **1** vs deriv. **5**	-3.592	1.233	No	ns	-14.95 to 7.767
deriv. **1** vs deriv. **6**	5.251	1.803	No	ns	-6.108 to 16.61
deriv. **1** vs deriv. **7**	-46.05	15.81	Yes	***	-57.40 to -34.69
deriv. **1** vs deriv. **8**	-27.30	9.373	Yes	***	-38.66 to -15.94
deriv. **1** vs deriv. **9**	3.536	1.214	No	ns	-7.823 to 14.89
deriv. **1** vs deriv. **10**	4.905	1.684	No	ns	-6.453 to 16.26
deriv. **1** vs deriv. **11**	7.201	2.472	No	ns	-4.157 to 18.56
deriv. **1** vs KA	-24.32	8.351	Yes	***	-35.68 to -12.96
deriv. **1** vs PTU	-31.31	10.75	Yes	***	-42.67 to -19.95
deriv. **2** vs deriv. **3**	-39.53	13.57	Yes	***	-50.89 to -28.17
deriv. **2** vs deriv. **4**	-22.34	7.671	Yes	***	-33.70 to -10.98
deriv. **2** vs deriv. **5**	2.224	0.7635	No	ns	-9.135 to 13.58

deriv. **2** vs deriv. **6**	11.07	3.799	No	ns	-0.2922 to 22.42
deriv. **2** vs deriv. **7**	-40.23	13.81	Yes	***	-51.59 to -28.87
deriv. **2** vs deriv. **8**	-21.48	7.376	Yes	***	-32.84 to -10.13
deriv. **2** vs deriv. **9**	9.351	3.211	No	ns	-2.007 to 20.71
deriv. **2** vs deriv. **10**	10.72	3.681	No	ns	-0.6378 to 22.08
deriv. **2** vs deriv. **11**	13.02	4.469	Yes	*	1.658 to 24.37
deriv. **2** vs KA	-18.51	6.354	Yes	***	-29.86 to -7.148
deriv. **2** vs PTU	-25.50	8.754	Yes	***	-36.86 to -14.14
deriv. **3** vs deriv. **4**	17.19	5.900	Yes	***	5.827 to 28.54
deriv. **3** vs deriv. **5**	41.75	14.33	Yes	***	30.39 to 53.11
deriv. **3** vs deriv. **6**	50.59	17.37	Yes	***	39.24 to 61.95
deriv. **3** vs deriv. **7**	-0.7038	0.2417	No	ns	-12.06 to 10.65
deriv. **3** vs deriv. **8**	18.04	6.195	Yes	***	6.685 to 29.40
deriv. **3** vs deriv. **9**	48.88	16.78	Yes	***	37.52 to 60.24
deriv. **3** vs deriv. **10**	50.25	17.25	Yes	***	38.89 to 61.61
deriv. **3** vs deriv. **11**	52.54	18.04	Yes	***	41.19 to 63.90
deriv. **3** vs KA	21.02	7.217	Yes	***	9.663 to 32.38
deriv. **3** vs PTU	14.03	4.817	Yes	**	2.672 to 25.39
deriv. **4** vs deriv. **5**	24.57	8.434	Yes	***	13.21 to 35.92
deriv. **4** vs deriv. **6**	33.41	11.47	Yes	***	22.05 to 44.77
deriv. **4** vs deriv. **7**	-17.89	6.142	Yes	***	-29.25 to -6.531
deriv. **4** vs deriv. **8**	0.8576	0.2945	No	ns	-10.50 to 12.22
deriv. **4** vs deriv. **9**	31.69	10.88	Yes	***	20.33 to 43.05
deriv. **4** vs deriv. **10**	33.06	11.35	Yes	***	21.70 to 44.42
deriv. **4** vs deriv. **11**	35.36	12.14	Yes	***	24.00 to 46.72
deriv. **4** vs KA	3.836	1.317	No	ns	-7.522 to 15.19
deriv. **4** vs PTU	-3.156	1.083	No	ns	-14.51 to 8.203
deriv. **5** vs deriv. **6**	8.842	3.036	No	ns	-2.516 to 20.20
deriv. **5** vs deriv. **7**	-42.45	14.58	Yes	***	-53.81 to -31.10
deriv. **5** vs deriv. **8**	-23.71	8.140	Yes	***	-35.07 to -12.35
deriv. **5** vs deriv. **9**	7.127	2.447	No	ns	-4.231 to 18.49
deriv. **5** vs deriv. **10**	8.497	2.917	No	ns	-2.862 to 19.86
deriv. **5** vs deriv. **11**	10.79	3.706	No	ns	-0.5654 to 22.15
deriv. **5** vs KA	-20.73	7.117	Yes	***	-32.09 to -9.372
deriv. **5** vs PTU	-27.72	9.518	Yes	***	-39.08 to -16.36
deriv. **6** vs deriv.**7**	-51.30	17.61	Yes	***	-62.66 to -39.94
deriv. **6** vs deriv.**8**	-32.55	11.18	Yes	***	-43.91 to -21.19
deriv. **6** vs deriv.**9**	-1.715	0.5889	No	ns	-13.07 to 9.643
deriv. **6** vs deriv.**10**	-0.3456	0.1187	No	ns	-11.70 to 11.01
deriv. **6** vs deriv.**11**	1.950	0.6697	No	ns	-9.408 to 13.31
deriv. **6** vs KA	-29.57	10.15	Yes	***	-40.93 to -18.21
deriv. **6** vs PTU	-36.56	12.55	Yes	***	-47.92 to -25.21
deriv. **7** vs deriv. **8**	18.75	6.437	Yes	***	7.389 to 30.11
deriv. **7** vs deriv. **9**	49.58	17.02	Yes	***	38.22 to 60.94
deriv. **7** vs deriv. **10**	50.95	17.49	Yes	***	39.59 to 62.31

deriv. **7** vs deriv. **11**	53.25	18.28	Yes	***	41.89 to 64.61
deriv. **7** vs KA	21.73	7.459	Yes	***	10.37 to 33.08
deriv. **7** vs PTU	14.73	5.059	Yes	**	3.375 to 26.09
deriv. **8** vs deriv. **9**	30.84	10.59	Yes	***	19.48 to 42.19
deriv. **8** vs deriv. **10**	32.20	11.06	Yes	***	20.85 to 43.56
deriv. **8** vs deriv. **11**	34.50	11.85	Yes	***	23.14 to 45.86
deriv. **8** vs KA	2.978	1.023	No	ns	-8.380 to 14.34
deriv. **8** vs PTU	-4.013	1.378	No	ns	-15.37 to 7.345
deriv. **9** vs deriv. **10**	1.370	0.4703	No	ns	-9.989 to 12.73
deriv. **9** vs deriv. **11**	3.666	1.259	No	ns	-7.693 to 15.02
deriv. **9** vs KA	-27.86	9.564	Yes	***	-39.22 to -16.50
deriv. **9** vs PTU	-34.85	11.96	Yes	***	-46.21 to -23.49
deriv. **10** vs deriv. **11**	2.296	0.7883	No	ns	-9.062 to 13.65
deriv. **10** vs KA	-29.23	10.03	Yes	***	-40.58 to -17.87
deriv. **10** vs PTU	-36.22	12.44	Yes	***	-47.58 to -24.86
deriv. **11** vs KA	-31.52	10.82	Yes	***	-42.88 to -20.16
deriv. **11** vs PTU	-38.51	13.22	Yes	***	-49.87 to -27.16
KA vs PTU	-6.991	2.400	No	ns	-18.35 to 4.367

KA: kojic acid, PTU: phenylthiourea

S63. Statistical analysis of Cu^{2+}-chelation efficacy in AAMA derivatives **1–11**, KA, and PTU

Table Analyzed	Deriv.1				
One-way analysis of variance					
P value	< 0.0001				
P value summary	***				
Are means signif. different? (P < 0.05)	Yes				
Number of groups	4				
F	48.49				
R square	0.9009				
Bartlett's test for equal variances					
Bartlett's statistic (corrected)	0.9490				
P value	0.8136				
P value summary	ns				
Do the variances differ signif. (P < 0.05)	No				
ANOVA Table	SS	df	MS		
Treatment (between columns)	1196	3	398.7		
Residual (within columns)	131.6	16	8.224		
Total	1328	19			
Bonferroni's Multiple Comparison Test	Mean Diff.	t	Significant? P < 0.05?	Summary	95% CI of diff
0 µM vs 3.2 µM	12.01	6.622	Yes	***	6.554 to 17.47
0 µM vs 8 µM	15.68	8.643	Yes	***	10.22 to 21.13
0 µM vs 20 µM	21.05	11.61	Yes	***	15.59 to 26.50
3.2 µM vs 8 µM	3.665	2.021	No	ns	-1.792 to 9.121
3.2 µM vs 20 µM	9.038	4.983	Yes	***	3.582 to 14.49
8 µM vs 20 µM	5.374	2.963	No	ns	-0.08256 to 10.83

S64. Statistical analysis of the cytotoxicity of derivative **1** in B16F10 cells

Table Analyzed	Deriv.2				
One-way analysis of variance					
P value	0.1140				
P value summary	ns				
Are means signif. different? (P < 0.05)	No				
Number of groups	4				
F	2.321				
R square	0.3032				
Bartlett's test for equal variances					
Bartlett's statistic (corrected)	2.462				
P value	0.4823				
P value summary	ns				
Do the variances differ signif. (P < 0.05)	No				
ANOVA Table	SS	df	MS		
Treatment (between columns)	55.40	3	18.47		
Residual (within columns)	127.3	16	7.957		
Total	182.7	19			
Bonferroni's Multiple Comparison Test	Mean Diff.	t	Significant? P < 0.05?	Summary	95% CI of diff
0 μM vs 3.2 μM	-1.665	0.9335	No	ns	-7.032 to 3.702
0 μM vs 8 μM	-4.424	2.480	No	ns	-9.791 to 0.9427
0 μM vs 20 μM	-3.238	1.815	No	ns	-8.605 to 2.129
3.2 μM vs 8 μM	-2.759	1.546	No	ns	-8.126 to 2.608
3.2 μM vs 20 μM	-1.572	0.8812	No	ns	-6.939 to 3.795
8 μM vs 20 μM	1.187	0.6652	No	ns	-4.180 to 6.554

S65. Statistical analysis of the cytotoxicity of derivative **2** in B16F10 cells

Table Analyzed	Deriv.3				
One-way analysis of variance					
P value	0.5364				
P value summary	ns				
Are means signif. different? (P < 0.05)	No				
Number of groups	4				
F	0.7534				
R square	0.1238				
Bartlett's test for equal variances					
Bartlett's statistic (corrected)	8.028				
P value	0.0454				
P value summary	*				
Do the variances differ signif. (P < 0.05)	Yes				
ANOVA Table	SS	df	MS		
Treatment (between columns)	16.47	3	5.491		
Residual (within columns)	116.6	16	7.289		
Total	133.1	19			
Bonferroni's Multiple Comparison Test	Mean Diff.	t	Significant? P < 0.05?	Summary	95% CI of diff
0 µM vs 3.2 µM	1.132	0.6630	No	ns	-4.005 to 6.269
0 µM vs 8 µM	-0.5167	0.3026	No	ns	-5.653 to 4.620
0 µM vs 20 µM	-1.375	0.8052	No	ns	-6.512 to 3.762
3.2 µM vs 8 µM	-1.649	0.9656	No	ns	-6.785 to 3.488
3.2 µM vs 20 µM	-2.507	1.468	No	ns	-7.644 to 2.630
8 µM vs 20 µM	-0.8582	0.5026	No	ns	-5.995 to 4.278

S66. Statistical analysis of the cytotoxicity of derivative **3** in B16F10 cells

Table Analyzed	Deriv.4				
One-way analysis of variance					
P value	0.3072				
P value summary	ns				
Are means signif. different? (P < 0.05)	No				
Number of groups	4				
F	1.305				
R square	0.1966				
Bartlett's test for equal variances					
Bartlett's statistic (corrected)	2.191				
P value	0.5336				
P value summary	ns				
Do the variances differ signif. (P < 0.05)	No				
ANOVA Table	SS	df	MS		
Treatment (between columns)	10.48	3	3.493		
Residual (within columns)	42.83	16	2.677		
Total	53.31	19			
Bonferroni's Multiple Comparison Test	Mean Diff.	t	Significant? P < 0.05?	Summary	95% CI of diff
0 µM vs 3.2 µM	0.06401	0.06186	No	ns	-3.049 to 3.177
0 µM vs 8 µM	0.9647	0.9323	No	ns	-2.148 to 4.078
0 µM vs 20 µM	-1.078	1.042	No	ns	-4.191 to 2.035
3.2 µM vs 8 µM	0.9007	0.8704	No	ns	-2.212 to 4.014
3.2 µM vs 20 µM	-1.142	1.103	No	ns	-4.255 to 1.971
8 µM vs 20 µM	-2.043	1.974	No	ns	-5.156 to 1.071

S67. Statistical analysis of the cytotoxicity of derivative **4** in B16F10 cells

Table Analyzed	Deriv.5				
One-way analysis of variance					
P value	0.6274				
P value summary	ns				
Are means signif. different? (P < 0.05)	No				
Number of groups	4				
F	0.5949				
R square	0.1003				
Bartlett's test for equal variances					
Bartlett's statistic (corrected)	6.733				
P value	0.0809				
P value summary	ns				
Do the variances differ signif. (P < 0.05)	No				
ANOVA Table	SS	df	MS		
Treatment (between columns)	9.049	3	3.016		
Residual (within columns)	81.13	16	5.071		
Total	90.18	19			
Bonferroni's Multiple Comparison Test	Mean Diff.	t	Significant? P < 0.05?	Summary	95% CI of diff
0 μM vs 3.2 μM	0.6751	0.4741	No	ns	-3.609 to 4.960
0 μM vs 8 μM	0.4861	0.3413	No	ns	-3.798 to 4.771
0 μM vs 20 μM	1.833	1.287	No	ns	-2.452 to 6.117
3.2 μM vs 8 μM	-0.1891	0.1328	No	ns	-4.474 to 4.095
3.2 μM vs 20 μM	1.158	0.8127	No	ns	-3.127 to 5.442
8 μM vs 20 μM	1.347	0.9455	No	ns	-2.938 to 5.631

S68. Statistical analysis of the cytotoxicity of derivative **5** in B16F10 cells

Table Analyzed	Deriv.6				
One-way analysis of variance					
P value	0.1004				
P value summary	ns				
Are means signif. different? (P < 0.05)	No				
Number of groups	4				
F	2.457				
R square	0.3154				
Bartlett's test for equal variances					
Bartlett's statistic (corrected)	1.955				
P value	0.5818				
P value summary	ns				
Do the variances differ signif. (P < 0.05)	No				
ANOVA Table	SS	df	MS		
Treatment (between columns)	37.19	3	12.40		
Residual (within columns)	80.73	16	5.045		
Total	117.9	19			
Bonferroni's Multiple Comparison Test	Mean Diff.	t	Significant? P < 0.05?	Summary	95% CI of diff
0 µM vs 3.2 µM	0.2739	0.1928	No	ns	-4.000 to 4.548
0 µM vs 8 µM	0.4481	0.3154	No	ns	-3.826 to 4.722
0 µM vs 20 µM	3.368	2.371	No	ns	-0.9054 to 7.642
3.2 µM vs 8 µM	0.1742	0.1226	No	ns	-4.100 to 4.448
3.2 µM vs 20 µM	3.094	2.178	No	ns	-1.179 to 7.368
8 µM vs 20 µM	2.920	2.056	No	ns	-1.354 to 7.194

S69. Statistical analysis of the cytotoxicity of derivative **6** in B16F10 cells

Table Analyzed	Deriv.7				
One-way analysis of variance					
P value	0.1262				
P value summary	ns				
Are means signif. different? (P < 0.05)	No				
Number of groups	4				
F	2.213				
R square	0.2933				
Bartlett's test for equal variances					
Bartlett's statistic (corrected)	0.5698				
P value	0.9033				
P value summary	ns				
Do the variances differ signif. (P < 0.05)	No				
ANOVA Table	SS	df	MS		
Treatment (between columns)	34.32	3	11.44		
Residual (within columns)	82.72	16	5.170		
Total	117.0	19			
Bonferroni's Multiple Comparison Test	Mean Diff.	t	Significant? P < 0.05?	Summary	95% CI of diff
0 µM vs 3.2 µM	1.952	1.358	No	ns	-2.374 to 6.279
0 µM vs 8 µM	2.455	1.707	No	ns	-1.871 to 6.781
0 µM vs 20 µM	3.630	2.524	No	ns	-0.6966 to 7.956
3.2 µM vs 8 µM	0.5027	0.3496	No	ns	-3.823 to 4.829
3.2 µM vs 20 µM	1.677	1.166	No	ns	-2.649 to 6.003
8 µM vs 20 µM	1.174	0.8166	No	ns	-3.152 to 5.501

S70. Statistical analysis of the cytotoxicity of derivative **7** in B16F10 cells

Table Analyzed	Deriv.8				
One-way analysis of variance					
P value	0.0324				
P value summary	*				
Are means signif. different? (P < 0.05)	Yes				
Number of groups	4				
F	3.757				
R square	0.4133				
Bartlett's test for equal variances					
Bartlett's statistic (corrected)	2.080				
P value	0.5560				
P value summary	ns				
Do the variances differ signif. (P < 0.05)	No				
ANOVA Table	SS	df	MS		
Treatment (between columns)	49.95	3	16.65		
Residual (within columns)	70.91	16	4.432		
Total	120.9	19			
Bonferroni's Multiple Comparison Test	Mean Diff.	t	Significant? P < 0.05?	Summary	95% CI of diff
0 µM vs 3.2 µM	-2.086	1.566	No	ns	-6.091 to 1.920
0 µM vs 8 µM	-1.721	1.292	No	ns	-5.726 to 2.285
0 µM vs 20 µM	1.895	1.423	No	ns	-2.110 to 5.901
3.2 µM vs 8 µM	0.3649	0.2740	No	ns	-3.641 to 4.370
3.2 µM vs 20 µM	3.981	2.990	No	ns	-0.02453 to 7.986
8 µM vs 20 µM	3.616	2.716	No	ns	-0.3894 to 7.621

S71. Statistical analysis of the cytotoxicity of derivative **8** in B16F10 cells

Table Analyzed	Deriv.9				
One-way analysis of variance					
P value	0.4885				
P value summary	ns				
Are means signif. different? (P < 0.05)	No				
Number of groups	4				
F	0.8489				
R square	0.1451				
ANOVA Table	SS	df	MS		
Treatment (between columns)	8.640	3	2.880		
Residual (within columns)	50.89	15	3.393		
Total	59.53	18			
Bonferroni's Multiple Comparison Test	Mean Diff.	t	Significant? P < 0.05?	Summary	95% CI of diff
0 μM vs 3.2 μM	1.625	1.315	No	ns	-2.127 to 5.376
0 μM vs 8 μM	0.1616	0.1388	No	ns	-3.375 to 3.699
0 μM vs 20 μM	-0.1705	0.1464	No	ns	-3.708 to 3.367
3.2 μM vs 8 μM	-1.463	1.184	No	ns	-5.215 to 2.289
3.2 μM vs 20 μM	-1.795	1.453	No	ns	-5.547 to 1.957
8 μM vs 20 μM	-0.3321	0.2851	No	ns	-3.869 to 3.205

S72. Statistical analysis of the cytotoxicity of derivative **9** in B16F10 cells

Table Analyzed	Deriv.10				
One-way analysis of variance					
P value	0.1938				
P value summary	ns				
Are means signif. different? (P < 0.05)	No				
Number of groups	4				
F	1.768				
R square	0.2489				
Bartlett's test for equal variances					
Bartlett's statistic (corrected)	2.271				
P value	0.5182				
P value summary	ns				
Do the variances differ signif. (P < 0.05)	No				
ANOVA Table	SS	df	MS		
Treatment (between columns)	19.66	3	6.552		
Residual (within columns)	59.31	16	3.707		
Total	78.97	19			
Bonferroni's Multiple Comparison Test	Mean Diff.	t	Significant? P < 0.05?	Summary	95% CI of diff
0 µM vs 3.2 µM	-2.651	2.177	No	ns	-6.314 to 1.012
0 µM vs 8 µM	-2.011	1.651	No	ns	-5.674 to 1.653
0 µM vs 20 µM	-1.927	1.583	No	ns	-5.591 to 1.736
3.2 µM vs 8 µM	0.6403	0.5258	No	ns	-3.023 to 4.303
3.2 µM vs 20 µM	0.7236	0.5942	No	ns	-2.940 to 4.387
8 µM vs 20 µM	0.08333	0.06843	No	ns	-3.580 to 3.747

S73. Statistical analysis of the cytotoxicity of derivative **10** in B16F10 cells

Table Analyzed	Deriv.11				
One-way analysis of variance					
P value	0.6701				
P value summary	ns				
Are means signif. different? (P < 0.05)	No				
Number of groups	4				
F	0.5301				
R square	0.1170				
ANOVA Table	SS	df	MS		
Treatment (between columns)	33.57	3	11.19		
Residual (within columns)	253.3	12	21.11		
Total	286.8	15			
Bonferroni's Multiple Comparison Test	Mean Diff.	t	Significant? P < 0.05?	Summary	95% CI of diff
0 µM vs 3.2 µM	-2.237	0.7257	No	ns	-11.95 to 7.480
0 µM vs 8 µM	-4.125	1.229	No	ns	-14.70 to 6.453
0 µM vs 20 µM	-1.414	0.4587	No	ns	-11.13 to 8.302
3.2 µM vs 8 µM	-1.888	0.5382	No	ns	-12.95 to 9.174
3.2 µM vs 20 µM	0.8228	0.2533	No	ns	-9.419 to 11.06
8 µM vs 20 µM	2.711	0.7727	No	ns	-8.351 to 13.77

S74. Statistical analysis of the cytotoxicity of derivative **11** in B16F10 cells

Table Analyzed	Melanin screening				
One-way analysis of variance					
P value	< 0.0001				
P value summary	***				
Are means signif. different? (P < 0.05)	Yes				
Number of groups	14				
F	46.95				
R square	0.9561				
ANOVA Table	SS	df	MS		
Treatment (between columns)	198409	13	15262		
Residual (within columns)	9102	28	325.1		
Total	207511	41			
Bonferroni's Multiple Comparison Test	Mean Diff.	t	Significant? P < 0.05?	Summary	95% CI of diff
Control vs α-MSH+IBMX	-150.4	10.22	Yes	***	-207.8 to -93.00
Control vs deriv. **2**	-65.17	4.427	Yes	*	-122.6 to -7.756
Control vs deriv. **3**	-184.1	12.50	Yes	***	-241.5 to -126.6
Control vs deriv. **4**	-157.9	10.73	Yes	***	-215.3 to -100.5
Control vs deriv. **5**	-15.46	1.050	No	ns	-72.87 to 41.95
Control vs deriv. **6**	-12.90	0.8763	No	ns	-70.31 to 44.51
Control vs deriv. **7**	-146.4	9.943	Yes	***	-203.8 to -88.97
Control vs deriv. **8**	-158.3	10.75	Yes	***	-215.7 to -100.8
Control vs deriv. **9**	-132.7	9.016	Yes	***	-190.1 to -75.32
Control vs deriv. **10**	-144.5	9.817	Yes	***	-201.9 to -87.11
Control vs deriv. **11**	-165.2	11.22	Yes	***	-222.6 to -107.8
Control vs KA	-167.2	11.36	Yes	***	-224.6 to -109.8
Control vs PTU	2.688	0.1826	No	ns	-54.72 to 60.10
α-MSH+IBMX vs deriv. **2**	85.24	5.790	Yes	***	27.83 to 142.7
α-MSH+IBMX vs deriv. **3**	-33.64	2.285	No	ns	-91.05 to 23.77
α-MSH+IBMX vs deriv. **4**	-7.496	0.5092	No	ns	-64.91 to 49.91
α-MSH+IBMX vs deriv. **5**	134.9	9.167	Yes	***	77.54 to 192.4
α-MSH+IBMX vs deriv. **6**	137.5	9.341	Yes	***	80.10 to 194.9
α-MSH+IBMX vs deriv. **7**	4.032	0.2739	No	ns	-53.38 to 61.44
α-MSH+IBMX vs deriv. **8**	-7.843	0.5327	No	ns	-65.25 to 49.57
α-MSH+IBMX vs deriv. **9**	17.68	1.201	No	ns	-39.73 to 75.09
α-MSH+IBMX vs deriv. **10**	5.889	0.4000	No	ns	-51.52 to 63.30
α-MSH+IBMX vs deriv. **11**	-14.78	1.004	No	ns	-72.19 to 42.63
α-MSH+IBMX vs KA	-16.81	1.142	No	ns	-74.22 to 40.60
α-MSH+IBMX vs PTU	153.1	10.40	Yes	***	95.69 to 210.5
deriv. **2** vs deriv. **3**	-118.9	8.076	Yes	***	-176.3 to -61.48
deriv. **2** vs deriv. **4**	-92.74	6.300	Yes	***	-150.1 to -35.33
deriv. **2** vs deriv. **5**	49.70	3.376	No	ns	-7.708 to 107.1

deriv. **2** vs deriv. **6**	52.27	3.550	No	ns	-5.144 to 109.7
deriv. **2** vs deriv. **7**	-81.21	5.517	Yes	***	-138.6 to -23.80
deriv. **2** vs deriv. **8**	-93.09	6.323	Yes	***	-150.5 to -35.68
deriv. **2** vs deriv. **9**	-67.56	4.589	Yes	**	-125.0 to -10.15
deriv. **2** vs deriv. **10**	-79.35	5.390	Yes	***	-136.8 to -21.94
deriv. **2** vs deriv. **11**	-100.0	6.795	Yes	***	-157.4 to -42.62
deriv. **2** vs KA	-102.1	6.932	Yes	***	-159.5 to -44.64
deriv. **2** vs PTU	67.85	4.609	Yes	**	10.44 to 125.3
deriv. **3** vs deriv. **4**	26.15	1.776	No	ns	-31.26 to 83.56
deriv. **3** vs deriv. **5**	168.6	11.45	Yes	***	111.2 to 226.0
deriv. **3** vs deriv. **6**	171.2	11.63	Yes	***	113.7 to 228.6
deriv. **3** vs deriv. **7**	37.67	2.559	No	ns	-19.73 to 95.08
deriv. **3** vs deriv. **8**	25.80	1.753	No	ns	-31.61 to 83.21
deriv. **3** vs deriv. **9**	51.32	3.486	No	ns	-6.086 to 108.7
deriv. **3** vs deriv. **10**	39.53	2.685	No	ns	-17.88 to 96.94
deriv. **3** vs deriv. **11**	18.86	1.281	No	ns	-38.55 to 76.27
deriv. **3** vs KA	16.84	1.144	No	ns	-40.57 to 74.24
deriv. **3** vs PTU	186.7	12.68	Yes	***	129.3 to 244.1
deriv. **4** vs deriv. **5**	142.4	9.676	Yes	***	85.03 to 199.9
deriv. **4** vs deriv. **6**	145.0	9.850	Yes	***	87.60 to 202.4
deriv. **4** vs deriv. **7**	11.53	0.7831	No	ns	-45.88 to 68.94
deriv. **4** vs deriv. **8**	-0.3464	0.02353	No	ns	-57.76 to 57.06
deriv. **4** vs deriv. **9**	25.18	1.710	No	ns	-32.23 to 82.59
deriv. **4** vs deriv. **10**	13.39	0.9092	No	ns	-44.02 to 70.79
deriv. **4** vs deriv. **11**	-7.288	0.4951	No	ns	-64.70 to 50.12
deriv. **4** vs KA	-9.311	0.6325	No	ns	-66.72 to 48.10
deriv. **4** vs PTU	160.6	10.91	Yes	***	103.2 to 218.0
deriv. **5** vs deriv. **6**	2.563	0.1741	No	ns	-54.85 to 59.97
deriv. **5** vs deriv. **7**	-130.9	8.893	Yes	***	-188.3 to -73.50
deriv. **5** vs deriv. **8**	-142.8	9.699	Yes	***	-200.2 to -85.38
deriv. **5** vs deriv. **9**	-117.3	7.966	Yes	***	-174.7 to -59.86
deriv. **5** vs deriv. **10**	-129.1	8.767	Yes	***	-186.5 to -71.65
deriv. **5** vs deriv. **11**	-149.7	10.17	Yes	***	-207.1 to -92.32
deriv. **5** vs KA	-151.8	10.31	Yes	***	-209.2 to -94.34
deriv. **5** vs PTU	18.15	1.233	No	ns	-39.26 to 75.56
deriv. **6** vs deriv. **7**	-133.5	9.067	Yes	***	-190.9 to -76.07
deriv. **6** vs deriv. **8**	-145.4	9.873	Yes	***	-202.8 to -87.94
deriv. **6** vs deriv. **9**	-119.8	8.140	Yes	***	-177.2 to -62.42
deriv. **6** vs deriv. **10**	-131.6	8.941	Yes	***	-189.0 to -74.21
deriv. **6** vs deriv. **11**	-152.3	10.35	Yes	***	-209.7 to -94.88
deriv. **6** vs KA	-154.3	10.48	Yes	***	-211.7 to -96.91
deriv. **6** vs PTU	15.59	1.059	No	ns	-41.82 to 73.00
deriv. **7** vs deriv. **8**	-11.87	0.8066	No	ns	-69.28 to 45.53
deriv. **7** vs deriv. **9**	13.65	0.9271	No	ns	-43.76 to 71.06
deriv. **7** vs deriv. **10**	1.857	0.1261	No	ns	-55.55 to 59.27

deriv. **7** vs deriv. **11**	-18.82	1.278	No	ns	-76.23 to 38.59
deriv. **7** vs KA	-20.84	1.416	No	ns	-78.25 to 36.57
deriv. **7** vs PTU	149.1	10.13	Yes	***	91.66 to 206.5
deriv. **8** vs deriv. **9**	25.52	1.734	No	ns	-31.89 to 82.93
deriv. **8** vs deriv. **10**	13.73	0.9328	No	ns	-43.68 to 71.14
deriv. **8** vs deriv. **11**	-6.942	0.4716	No	ns	-64.35 to 50.47
deriv. **8** vs KA	-8.965	0.6090	No	ns	-66.37 to 48.44
deriv. **8** vs PTU	160.9	10.93	Yes	***	103.5 to 218.3
deriv. **9** vs deriv. **10**	-11.79	0.8010	No	ns	-69.20 to 45.62
deriv. **9** vs deriv. **11**	-32.47	2.205	No	ns	-89.87 to 24.94
deriv. **9** vs KA	-34.49	2.343	No	ns	-91.90 to 22.92
deriv. **9** vs PTU	135.4	9.199	Yes	***	78.01 to 192.8
deriv. **10** vs deriv. **11**	-20.67	1.404	No	ns	-78.08 to 36.74
deriv. **10** vs KA	-22.70	1.542	No	ns	-80.11 to 34.71
deriv. **10** vs PTU	147.2	10.00	Yes	***	89.80 to 204.6
deriv. **11** vs KA	-2.023	0.1374	No	ns	-59.43 to 55.39
deriv. **11** vs PTU	167.9	11.40	Yes	***	110.5 to 225.3
KA vs PTU	169.9	11.54	Yes	***	112.5 to 227.3

KA: kojic acid, PTU: phenylthiourea

S75. Statistical analysis of the inhibitory effect of derivatives **2–11**, KA, and PTU on melanin formation in B16F10 cells

Table Analyzed	Deriv. **2**_mel				
One-way analysis of variance					
P value	< 0.0001				
P value summary	***				
Are means signif. different? (P < 0.05)	Yes				
Number of groups	9				
F	56.33				
R square	0.9616				
ANOVA Table	SS	df	MS		
Treatment (between columns)	152886	8	19111		
Residual (within columns)	6107	18	339.3		
Total	158993	26			
Bonferroni's Multiple Comparison Test	Mean Diff.	t	Significant? P < 0.05?	Summary	95% CI of diff
Control vs α-MSH+IBMX	-204.2	13.58	Yes	***	-260.9 to -147.4
Control vs deriv. **2**-3.2 μM	-199.3	13.25	Yes	***	-256.1 to -142.6
Control vs deriv. **2**-8 μM	-147.7	9.823	Yes	***	-204.5 to -90.97
Control vs deriv. **2**-20 μM	-91.37	6.075	Yes	***	-148.1 to -34.61
Control vs PTU-3.2 μM	-135.7	9.023	Yes	***	-192.5 to -78.93
Control vs PTU-8 μM	-48.59	3.231	No	ns	-105.4 to 8.169
Control vs PTU-20 μM	10.82	0.7197	No	ns	-45.94 to 67.58
Control vs KA-20 μM	-146.0	9.710	Yes	***	-202.8 to -89.27
α-MSH+IBMX vs deriv. **2**-3.2 μM	4.841	0.3219	No	ns	-51.92 to 61.60
α-MSH+IBMX vs deriv. **2**-8 μM	56.45	3.754	No	ns	-0.3093 to 113.2
α-MSH+IBMX vs deriv. **2**-20 μM	112.8	7.501	Yes	***	56.05 to 169.6
α-MSH+IBMX vs PTU-3.2 μM	68.49	4.554	Yes	**	11.73 to 125.2
α-MSH+IBMX vs PTU-8 μM	155.6	10.35	Yes	***	98.83 to 212.3
α-MSH+IBMX vs PTU-20 μM	215.0	14.30	Yes	***	158.2 to 271.8
α-MSH+IBMX vs KA-20 μM	58.14	3.866	Yes	*	1.384 to 114.9
deriv. **2**-3.2 μM vs deriv. **2**-8 μM	51.61	3.432	No	ns	-5.150 to 108.4
deriv. **2**-3.2 μM vs deriv. **2**-20 μM	108.0	7.179	Yes	***	51.21 to 164.7
deriv. **2**-3.2 μM vs PTU-3.2 μM	63.65	4.232	Yes	*	6.887 to 120.4
deriv. **2**-3.2 μM vs PTU-8 μM	150.7	10.02	Yes	***	93.98 to 207.5
deriv. **2**-3.2 μM vs PTU-20 μM	210.2	13.97	Yes	***	153.4 to 266.9
deriv. **2**-3.2 μM vs KA-20 μM	53.30	3.544	No	ns	-3.457 to 110.1
deriv. **2**-8 μM vs deriv. **2**-20 μM	56.36	3.748	No	ns	-0.4014 to 113.1
deriv. **2**-8 μM vs PTU-3.2 μM	12.04	0.8004	No	ns	-44.72 to 68.80
deriv. **2**-8 μM vs PTU-8 μM	99.13	6.592	Yes	***	42.37 to 155.9
deriv. **2**-8 μM vs PTU-20 μM	158.5	10.54	Yes	***	101.8 to 215.3
deriv. **2**-8 μM vs KA-20 μM	1.693	0.1126	No	ns	-55.07 to 58.45
deriv. **2**-20 μM vs PTU-3.2 μM	-44.32	2.947	No	ns	-101.1 to 12.44
deriv. **2**-20 μM vs PTU-8 μM	42.78	2.844	No	ns	-13.99 to 99.54

deriv. **2**-20 µM vs PTU-20 µM	102.2	6.795	Yes	***	45.43 to 159.0
deriv. **2**-20 µM vs KA-20 µM	-54.67	3.635	No	ns	-111.4 to 2.095
PTU-3.2 µM vs PTU-8 µM	87.10	5.791	Yes	***	30.34 to 143.9
PTU-3.2 µM vs PTU-20 µM	146.5	9.742	Yes	***	89.75 to 203.3
PTU-3.2 µM vs KA-20 µM	-10.34	0.6878	No	ns	-67.10 to 46.42
PTU-8 µM vs PTU-20 µM	59.41	3.951	Yes	*	2.654 to 116.2
PTU-8 µM vs KA-20 µM	-97.44	6.479	Yes	***	-154.2 to -40.68
PTU-20 µM vs KA-20 µM	-156.9	10.43	Yes	***	-213.6 to -100.1

KA: kojic acid, PTU: phenylthiourea

S76. Statistical analysis of the inhibitory effect of derivative **2**, PTU, and KA on melanin formation in B16F10 cells

Table Analyzed	Deriv. **5**-mel				
One-way analysis of variance					
P value	< 0.0001				
P value summary	***				
Are means signif. different? (P < 0.05)	Yes				
Number of groups	9				
F	125.8				
R square	0.9824				
ANOVA Table	SS	df	MS		
Treatment (between columns)	146791	8	18349		
Residual (within columns)	2626	18	145.9		
Total	149417	26			
Bonferroni's Multiple Comparison Test	Mean Diff.	t	Significant? P < 0.05?	Summary	95% CI of diff
Control vs α-MSH+IBMX	-204.2	20.70	Yes	***	-241.4 to -167.0
Control vs deriv. **5**-3.2 µM	-148.5	15.06	Yes	***	-185.8 to -111.3
Control vs deriv. **5**-8 µM	-34.09	3.457	No	ns	-71.31 to 3.133
Control vs deriv. **5**-20 µM	-19.90	2.018	No	ns	-57.12 to 17.32
Control vs PTU-3.2 µM	-135.7	13.76	Yes	***	-172.9 to -98.47
Control vs PTU-8 µM	-48.59	4.927	Yes	**	-85.81 to -11.37
Control vs PTU-20 µM	10.82	1.097	No	ns	-26.40 to 48.04
Control vs KA-20 µM	-146.0	14.81	Yes	***	-183.3 to -108.8
α-MSH+IBMX vs deriv. **5**-3.2 µM	55.64	5.642	Yes	***	18.42 to 92.86
α-MSH+IBMX vs deriv. **5**-8 µM	170.1	17.25	Yes	***	132.9 to 207.3
α-MSH+IBMX vs deriv. **5**-20 µM	184.3	18.69	Yes	***	147.1 to 221.5
α-MSH+IBMX vs PTU-3.2 µM	68.49	6.945	Yes	***	31.27 to 105.7
α-MSH+IBMX vs PTU-8 µM	155.6	15.78	Yes	***	118.4 to 192.8
α-MSH+IBMX vs PTU-20 µM	215.0	21.80	Yes	***	177.8 to 252.2
α-MSH+IBMX vs KA-20 µM	58.14	5.896	Yes	***	20.92 to 95.37
deriv. **5**-3.2 µM vs deriv. **5**-8 µM	114.4	11.61	Yes	***	77.23 to 151.7
deriv. **5**-3.2 µM vs deriv. **5**-20 µM	128.6	13.04	Yes	***	91.42 to 165.9
deriv. **5**-3.2 µM vs PTU-3.2 µM	12.85	1.303	No	ns	-24.37 to 50.07
deriv. **5**-3.2 µM vs PTU-8 µM	99.94	10.13	Yes	***	62.72 to 137.2
deriv. **5**-3.2 µM vs PTU-20 µM	159.4	16.16	Yes	***	122.1 to 196.6
deriv. **5**-3.2 µM vs KA-20 µM	2.503	0.2538	No	ns	-34.72 to 39.72
deriv. **5**-8 µM vs deriv. **5**-20 µM	14.19	1.439	No	ns	-23.03 to 51.41
deriv. **5**-8 µM vs PTU-3.2 µM	-101.6	10.30	Yes	***	-138.8 to -64.38
deriv. **5**-8 µM vs PTU-8 µM	-14.50	1.471	No	ns	-51.72 to 22.72
deriv. **5**-8 µM vs PTU-20 µM	44.91	4.554	Yes	**	7.690 to 82.13
deriv. **5**-8 µM vs KA-20 µM	-111.9	11.35	Yes	***	-149.2 to -74.72
deriv. **5**-20 µM vs PTU-3.2 µM	-115.8	11.74	Yes	***	-153.0 to -78.57
deriv. **5**-20 µM vs PTU-8 µM	-28.70	2.910	No	ns	-65.92 to 8.526

deriv. **5**-20 µM vs PTU-20 µM	30.72	3.115	No	ns	-6.501 to 67.94
deriv. **5**-20 µM vs KA-20 µM	-126.1	12.79	Yes	***	-163.4 to -88.92
PTU-3.2 µM vs PTU-8 µM	87.10	8.832	Yes	***	49.88 to 124.3
PTU-3.2 µM vs PTU-20 µM	146.5	14.86	Yes	***	109.3 to 183.7
PTU-3.2 µM vs KA-20 µM	-10.34	1.049	No	ns	-47.57 to 26.88
PTU-8 µM vs PTU-20 µM	59.41	6.025	Yes	***	22.19 to 96.64
PTU-8 µM vs KA-20 µM	-97.44	9.881	Yes	***	-134.7 to -60.22
PTU-20 µM vs KA-20 µM	-156.9	15.91	Yes	***	-194.1 to -119.6

KA: kojic acid, PTU: phenylthiourea

S77. Statistical analysis of the inhibitory effect of derivative **5**, PTU, and KA on melanin formation in B16F10 cells

Table Analyzed	Deriv. **6**-mel				
One-way analysis of variance					
P value	< 0.0001				
P value summary	***				
Are means signif. different? (P < 0.05)	Yes				
Number of groups	9				
F	108.9				
R square	0.9798				
ANOVA Table	SS	df	MS		
Treatment (between columns)	150501	8	18813		
Residual (within columns)	3110	18	172.8		
Total	153611	26			
Bonferroni's Multiple Comparison Test	Mean Diff.	t	Significant? P < 0.05?	Summary	95% CI of diff
Control vs α-MSH+IBMX	-204.2	19.03	Yes	***	-244.7 to -163.7
Control vs deriv. **6**-3.2 μM	-151.9	14.15	Yes	***	-192.4 to -111.4
Control vs deriv. **6**-8 μM	-45.67	4.255	Yes	*	-86.17 to -5.161
Control vs deriv. **6**-20 μM	-7.105	0.6620	No	ns	-47.61 to 33.40
Control vs PTU-3.2 μM	-135.7	12.64	Yes	***	-176.2 to -95.18
Control vs PTU-8 μM	-48.59	4.528	Yes	**	-89.10 to -8.087
Control vs PTU-20 μM	10.82	1.008	No	ns	-29.68 to 51.33
Control vs KA-20 μM	-146.0	13.61	Yes	***	-186.5 to -105.5
α-MSH+IBMX vs deriv. **6**-3.2 μM	52.31	4.874	Yes	**	11.81 to 92.81
α-MSH+IBMX vs deriv. **6**-8 μM	158.5	14.77	Yes	***	118.0 to 199.0
α-MSH+IBMX vs deriv. **6**-20 μM	197.1	18.36	Yes	***	156.6 to 237.6
α-MSH+IBMX vs PTU-3.2 μM	68.49	6.382	Yes	***	27.98 to 109.0
α-MSH+IBMX vs PTU-8 μM	155.6	14.50	Yes	***	115.1 to 196.1
α-MSH+IBMX vs PTU-20 μM	215.0	20.03	Yes	***	174.5 to 255.5
α-MSH+IBMX vs KA-20 μM	58.14	5.418	Yes	**	17.64 to 98.65
deriv. **6**-3.2 μM vs deriv. **6**-8 μM	106.2	9.896	Yes	***	65.70 to 146.7
deriv. **6**-3.2 μM vs deriv. **6**-20 μM	144.8	13.49	Yes	***	104.3 to 185.3
deriv. **6**-3.2 μM vs PTU-3.2 μM	16.18	1.508	No	ns	-24.33 to 56.68
deriv. **6**-3.2 μM vs PTU-8 μM	103.3	9.623	Yes	***	62.77 to 143.8
deriv. **6**-3.2 μM vs PTU-20 μM	162.7	15.16	Yes	***	122.2 to 203.2
deriv. **6**-3.2 μM vs KA-20 μM	5.835	0.5437	No	ns	-34.67 to 46.34
deriv. **6**-8 μM vs deriv. **6**-20 μM	38.56	3.593	No	ns	-1.944 to 79.07
deriv. **6**-8 μM vs PTU-3.2 μM	-90.02	8.389	Yes	***	-130.5 to -49.52
deriv. **6**-8 μM vs PTU-8 μM	-2.927	0.2727	No	ns	-43.43 to 37.58
deriv. **6**-8 μM vs PTU-20 μM	56.49	5.264	Yes	**	15.98 to 96.99
deriv. **6**-8 μM vs KA-20 μM	-100.4	9.352	Yes	***	-140.9 to -59.86
deriv. **6**-20 μM vs PTU-3.2 μM	-128.6	11.98	Yes	***	-169.1 to -88.08
deriv. **6**-20 μM vs PTU-8 μM	-41.49	3.866	Yes	*	-81.99 to -0.9825

deriv. **6**-20 µM vs PTU-20 µM	17.93	1.670	No	ns	-22.58 to 58.43
deriv. **6**-20 µM vs KA-20 µM	-138.9	12.95	Yes	***	-179.4 to -98.42
PTU-3.2 µM vs PTU-8 µM	87.10	8.116	Yes	***	46.59 to 127.6
PTU-3.2 µM vs PTU-20 µM	146.5	13.65	Yes	***	106.0 to 187.0
PTU-3.2 µM vs KA-20 µM	-10.34	0.9639	No	ns	-50.85 to 30.16
PTU-8 µM vs PTU-20 µM	59.41	5.536	Yes	**	18.91 to 99.92
PTU-8 µM vs KA-20 µM	-97.44	9.080	Yes	***	-137.9 to -56.94
PTU-20 µM vs KA-20 µM	-156.9	14.62	Yes	***	-197.4 to -116.4

KA: kojic acid, PTU: phenylthiourea

S78. Statistical analysis of the inhibitory effect of derivative **6**, PTU, and KA on melanin formation in B16F10 cells

Table Analyzed	dorsal				
One-way analysis of variance					
P value	< 0.0001				
P value summary	***				
Are means signif. different? (P < 0.05)	Yes				
Number of groups	5				
F	11.40				
R square	0.7169				
ANOVA Table	SS	df	MS		
Treatment (between columns)	58521	4	14630		
Residual (within columns)	23110	18	1284		
Total	81631	22			
Bonferroni's Multiple Comparison Test	Mean Diff.	t	Significant? P < 0.05?	Summary	95% CI of diff
Control vs KA	72.80	2.873	No	ns	-8.189 to 153.8
Control vs deriv. **5**-0.01 μM	51.89	2.048	No	ns	-29.10 to 132.9
Control vs deriv. **5**-0.03 μM	65.99	2.853	No	ns	-7.942 to 139.9
Control vs deriv. **5**-0.1 μM	156.8	6.524	Yes	***	79.99 to 233.7
KA vs deriv. **5**-0.01 μM	-20.91	0.8252	No	ns	-101.9 to 60.08
KA vs deriv. **5**-0.03 μM	-6.809	0.2944	No	ns	-80.74 to 67.12
KA vs deriv. **5**-0.1 μM	84.02	3.495	Yes	*	7.184 to 160.9
deriv. **5**-0.01 μM vs deriv. **5**-0.03 μM	14.10	0.6095	No	ns	-59.84 to 88.03
deriv. **5**-0.01 μM vs deriv. **5**-0.1 μM	104.9	4.365	Yes	**	28.09 to 181.8
deriv. **5**-0.03 μM vs deriv. **5**-0.1 μM	90.83	4.186	Yes	**	21.47 to 160.2

KA: kojic acid

S79. Statistical analysis of the pigmentation inhibition effect of derivative **5** and KA on the zebrafish dorsal view

Table Analyzed	lateral				
One-way analysis of variance					
P value	< 0.0001				
P value summary	***				
Are means signif. different? (P < 0.05)	Yes				
Number of groups	5				
F	11.74				
R square	0.7229				
ANOVA Table	SS	df	MS		
Treatment (between columns)	42059	4	10515		
Residual (within columns)	16119	18	895.5		
Total	58178	22			
Bonferroni's Multiple Comparison Test	Mean Diff.	t	Significant? P < 0.05?	Summary	95% CI of diff
Control vs KA	64.52	3.049	No	ns	-3.117 to 132.2
Control vs deriv. **5**-0.01 μM	2.498	0.1181	No	ns	-65.14 to 70.14
Control vs deriv. **5**-0.03 μM	22.58	1.169	No	ns	-39.17 to 84.32
Control vs deriv. **5**-0.1 μM	112.0	5.577	Yes	***	47.79 to 176.1
KA vs deriv. **5**-0.01 μM	-62.02	2.931	No	ns	-129.7 to 5.615
KA vs deriv. **5**-0.03 μM	-41.95	2.171	No	ns	-103.7 to 19.80
KA vs deriv. **5**-0.1 μM	47.43	2.363	No	ns	-16.73 to 111.6
deriv. **5**-0.01 μM vs deriv. **5**-0.03 μM	20.08	1.039	No	ns	-41.67 to 81.82
deriv. **5**-0.01 μM vs deriv. **5**-0.1 μM	109.5	5.453	Yes	***	45.29 to 173.6
deriv. **5**-0.03 μM vs deriv. **5**-0.1 μM	89.38	4.933	Yes	**	31.46 to 147.3

KA: kojic acid

S80. Statistical analysis of the pigmentation inhibition effect of derivative **5** and KA on the zebrafish lateral view

Table Analyzed	Deriv. 1				
One-way analysis of variance					
P value	0.2918				
P value summary	ns				
Are means signif. different? (P < 0.05)	No				
Number of groups	4				
F	1.356				
R square	0.2027				
Bartlett's test for equal variances					
Bartlett's statistic (corrected)	6.751				
P value	0.0803				
P value summary	ns				
Do the variances differ signif. (P < 0.05)	No				
ANOVA Table	SS	df	MS		
Treatment (between columns)	31.45	3	10.48		
Residual (within columns)	123.7	16	7.729		
Total	155.1	19			
Bonferroni's Multiple Comparison Test	Mean Diff.	t	Significant? P < 0.05?	Summary	95% CI of diff
0 μM vs 3.2 μM	-1.336	0.7596	No	ns	-6.625 to 3.954
0 μM vs 8 μM	-0.4928	0.2802	No	ns	-5.782 to 4.797
0 μM vs 20 μM	-3.287	1.869	No	ns	-8.577 to 2.003
3.2 μM vs 8 μM	0.8429	0.4794	No	ns	-4.447 to 6.132
3.2 μM vs 20 μM	-1.951	1.110	No	ns	-7.241 to 3.338
8 μM vs 20 μM	-2.794	1.589	No	ns	-8.084 to 2.495

S81. Statistical analysis of the cytotoxicity of derivative **1** in Hs27 cells

Table Analyzed	Deriv. 2				
One-way analysis of variance					
P value	0.1516				
P value summary	ns				
Are means signif. different? (P < 0.05)	No				
Number of groups	4				
F	2.061				
R square	0.3063				
ANOVA Table	SS	df	MS		
Treatment (between columns)	66.58	3	22.19		
Residual (within columns)	150.8	14	10.77		
Total	217.3	17			
Bonferroni's Multiple Comparison Test	Mean Diff.	t	Significant? P < 0.05?	Summary	95% CI of diff
0 μM vs 3.2 μM	-2.666	1.211	No	ns	-9.421 to 4.090
0 μM vs 8 μM	-5.450	2.476	No	ns	-12.20 to 1.306
0 μM vs 20 μM	-2.838	1.367	No	ns	-9.207 to 3.531
3.2 μM vs 8 μM	-2.784	1.200	No	ns	-9.905 to 4.337
3.2 μM vs 20 μM	-0.1726	0.07841	No	ns	-6.928 to 6.583
8 μM vs 20 μM	2.611	1.186	No	ns	-4.144 to 9.367

S82. Statistical analysis of the cytotoxicity of derivative **2** in Hs27 cells

Table Analyzed	Deriv. 3				
One-way analysis of variance					
P value	0.6112				
P value summary	ns				
Are means signif. different? (P < 0.05)	No				
Number of groups	4				
F	0.6255				
R square	0.1261				
ANOVA Table	SS	df	MS		
Treatment (between columns)	25.94	3	8.648		
Residual (within columns)	179.7	13	13.83		
Total	205.7	16			
Bonferroni's Multiple Comparison Test	Mean Diff.	t	Significant? P < 0.05?	Summary	95% CI of diff
0 µM vs 3.2 µM	-3.327	1.334	No	ns	-11.08 to 4.423
0 µM vs 8 µM	-0.9173	0.3677	No	ns	-8.667 to 6.833
0 µM vs 20 µM	-0.9118	0.3655	No	ns	-8.662 to 6.838
3.2 µM vs 8 µM	2.410	0.9165	No	ns	-5.760 to 10.58
3.2 µM vs 20 µM	2.415	0.9185	No	ns	-5.754 to 10.58
8 µM vs 20 µM	0.005463	0.002078	No	ns	-8.164 to 8.175

S83. Statistical analysis of the cytotoxicity of derivative **3** in Hs27 cells

Table Analyzed	Deriv. 4				
One-way analysis of variance					
P value	0.0434				
P value summary	*				
Are means signif. different? (P < 0.05)	Yes				
Number of groups	4				
F	3.459				
R square	0.4089				
ANOVA Table	SS	df	MS		
Treatment (between columns)	79.33	3	26.44		
Residual (within columns)	114.7	15	7.646		
Total	194.0	18			
Bonferroni's Multiple Comparison Test	Mean Diff.	t	Significant? P < 0.05?	Summary	95% CI of diff
0 µM vs 3.2 µM	-4.878	2.789	No	ns	-10.19 to 0.4324
0 µM vs 8 µM	-3.025	1.631	No	ns	-8.657 to 2.607
0 µM vs 20 µM	-0.3429	0.1961	No	ns	-5.653 to 4.967
3.2 µM vs 8 µM	1.852	0.9987	No	ns	-3.780 to 7.485
3.2 µM vs 20 µM	4.535	2.593	No	ns	-0.7753 to 9.845
8 µM vs 20 µM	2.682	1.446	No	ns	-2.950 to 8.314

S84. Statistical analysis of the cytotoxicity of derivative **4** in Hs27 cells

Table Analyzed	Deriv. 5				
One-way analysis of variance					
P value	0.1879				
P value summary	ns				
Are means signif. different? (P < 0.05)	No				
Number of groups	4				
F	1.814				
R square	0.2662				
ANOVA Table	SS	df	MS		
Treatment (between columns)	24.50	3	8.168		
Residual (within columns)	67.55	15	4.503		
Total	92.05	18			
Bonferroni's Multiple Comparison Test	Mean Diff.	t	Significant? P < 0.05?	Summary	95% CI of diff
0 µM vs 3.2 µM	2.363	1.760	No	ns	-1.712 to 6.438
0 µM vs 8 µM	2.074	1.546	No	ns	-2.001 to 6.150
0 µM vs 20 µM	-0.1015	0.07130	No	ns	-4.424 to 4.221
3.2 µM vs 8 µM	-0.2883	0.2148	No	ns	-4.363 to 3.787
3.2 µM vs 20 µM	-2.464	1.731	No	ns	-6.787 to 1.858
8 µM vs 20 µM	-2.176	1.529	No	ns	-6.498 to 2.146

S85. Statistical analysis of the cytotoxicity of derivative **5** in Hs27 cells

Table Analyzed	Deriv. 6				
One-way analysis of variance					
P value	0.3166				
P value summary	ns				
Are means signif. different? (P < 0.05)	No				
Number of groups	4				
F	1.290				
R square	0.2165				
ANOVA Table	SS	df	MS		
Treatment (between columns)	38.77	3	12.92		
Residual (within columns)	140.3	14	10.02		
Total	179.1	17			
Bonferroni's Multiple Comparison Test	Mean Diff.	t	Significant? P < 0.05?	Summary	95% CI of diff
0 µM vs 3.2 µM	-1.793	0.8441	No	ns	-8.309 to 4.724
0 µM vs 8 µM	1.599	0.7532	No	ns	-4.917 to 8.116
0 µM vs 20 µM	2.028	1.013	No	ns	-4.116 to 8.172
3.2 µM vs 8 µM	3.392	1.515	No	ns	-3.477 to 10.26
3.2 µM vs 20 µM	3.820	1.799	No	ns	-2.696 to 10.34
8 µM vs 20 µM	0.4283	0.2017	No	ns	-6.088 to 6.945

S86. Statistical analysis of the cytotoxicity of derivative **6** in Hs27 cells

Table Analyzed	Deriv. 7				
One-way analysis of variance					
P value	0.4897				
P value summary	ns				
Are means signif. different? (P < 0.05)	No				
Number of groups	4				
F	0.8495				
R square	0.1540				
ANOVA Table	SS	df	MS		
Treatment (between columns)	30.16	3	10.05		
Residual (within columns)	165.7	14	11.84		
Total	195.9	17			
Bonferroni's Multiple Comparison Test	Mean Diff.	t	Significant? P < 0.05?	Summary	95% CI of diff
0 µM vs 3.2 µM	3.182	1.379	No	ns	-3.900 to 10.26
0 µM vs 8 µM	1.304	0.5995	No	ns	-5.373 to 7.982
0 µM vs 20 µM	2.941	1.274	No	ns	-4.141 to 10.02
3.2 µM vs 8 µM	-1.877	0.8134	No	ns	-8.959 to 5.205
3.2 µM vs 20 µM	-0.2410	0.09905	No	ns	-7.706 to 7.224
8 µM vs 20 µM	1.636	0.7090	No	ns	-5.446 to 8.718

S87. Statistical analysis of the cytotoxicity of derivative **7** in Hs27 cells

Table Analyzed	Deriv. 8				
One-way analysis of variance					
P value	0.8219				
P value summary	ns				
Are means signif. different? (P < 0.05)	No				
Number of groups	4				
F	0.3042				
R square	0.05396				
Bartlett's test for equal variances					
Bartlett's statistic (corrected)	5.662				
P value	0.1293				
P value summary	ns				
Do the variances differ signif. (P < 0.05)	No				
ANOVA Table	SS	df	MS		
Treatment (between columns)	5.662	3	1.887		
Residual (within columns)	99.28	16	6.205		
Total	104.9	19			
Bonferroni's Multiple Comparison Test	Mean Diff.	t	Significant? P < 0.05?	Summary	95% CI of diff
0 µM vs 3.2 µM	0.9246	0.5869	No	ns	-3.815 to 5.664
0 µM vs 8 µM	-0.5371	0.3409	No	ns	-5.276 to 4.202
0 µM vs 20 µM	0.3575	0.2269	No	ns	-4.382 to 5.097
3.2 µM vs 8 µM	-1.462	0.9279	No	ns	-6.201 to 3.278
3.2 µM vs 20 µM	-0.5672	0.3600	No	ns	-5.306 to 4.172
8 µM vs 20 µM	0.8946	0.5679	No	ns	-3.845 to 5.634

S88. Statistical analysis of the cytotoxicity of derivative **8** in Hs27 cells

Table Analyzed	Deriv. 9				
One-way analysis of variance					
P value	0.2061				
P value summary	ns				
Are means signif. different? (P < 0.05)	No				
Number of groups	4				
F	1.705				
R square	0.2422				
Bartlett's test for equal variances					
Bartlett's statistic (corrected)	5.703				
P value	0.1270				
P value summary	ns				
Do the variances differ signif. (P < 0.05)	No				
ANOVA Table	SS	df	MS		
Treatment (between columns)	52.80	3	17.60		
Residual (within columns)	165.2	16	10.32		
Total	218.0	19			
Bonferroni's Multiple Comparison Test	Mean Diff.	t	Significant? P < 0.05?	Summary	95% CI of diff
0 µM vs 3.2 µM	1.138	0.5602	No	ns	-4.975 to 7.252
0 µM vs 8 µM	-3.285	1.617	No	ns	-9.399 to 2.828
0 µM vs 20 µM	-0.6068	0.2986	No	ns	-6.720 to 5.507
3.2 µM vs 8 µM	-4.424	2.177	No	ns	-10.54 to 1.690
3.2 µM vs 20 µM	-1.745	0.8588	No	ns	-7.859 to 4.368
8 µM vs 20 µM	2.678	1.318	No	ns	-3.435 to 8.792

S89. Statistical analysis of the cytotoxicity of derivative **9** in Hs27 cells

Table Analyzed	Deriv. 10				
One-way analysis of variance					
P value	0.2272				
P value summary	ns				
Are means signif. different? (P < 0.05)	No				
Number of groups	4				
F	1.607				
R square	0.2315				
Bartlett's test for equal variances					
Bartlett's statistic (corrected)	6.307				
P value	0.0976				
P value summary	ns				
Do the variances differ signif. (P < 0.05)	No				
ANOVA Table	SS	df	MS		
Treatment (between columns)	21.13	3	7.042		
Residual (within columns)	70.13	16	4.383		
Total	91.26	19			
Bonferroni's Multiple Comparison Test	Mean Diff.	t	Significant? P < 0.05?	Summary	95% CI of diff
0 μM vs 3.2 μM	-1.864	1.408	No	ns	-5.848 to 2.119
0 μM vs 8 μM	-1.270	0.9594	No	ns	-5.254 to 2.713
0 μM vs 20 μM	-2.838	2.143	No	ns	-6.821 to 1.146
3.2 μM vs 8 μM	0.5940	0.4486	No	ns	-3.389 to 4.577
3.2 μM vs 20 μM	-0.9734	0.7352	No	ns	-4.957 to 3.010
8 μM vs 20 μM	-1.567	1.184	No	ns	-5.551 to 2.416

S90. Statistical analysis of the cytotoxicity of derivative **10** in Hs27 cells

Table Analyzed	Deriv. 11				
One-way analysis of variance					
P value	0.1783				
P value summary	ns				
Are means signif. different? (P < 0.05)	No				
Number of groups	4				
F	1.853				
R square	0.2578				
Bartlett's test for equal variances					
Bartlett's statistic (corrected)	9.907				
P value	0.0194				
P value summary	*				
Do the variances differ signif. (P < 0.05)	Yes				
ANOVA Table	SS	df	MS		
Treatment (between columns)	50.41	3	16.80		
Residual (within columns)	145.1	16	9.069		
Total	195.5	19			
Bonferroni's Multiple Comparison Test	Mean Diff.	t	Significant? P < 0.05?	Summary	95% CI of diff
0 µM vs 3.2 µM	-2.964	1.556	No	ns	-8.694 to 2.765
0 µM vs 8 µM	0.5280	0.2772	No	ns	-5.202 to 6.258
0 µM vs 20 µM	-2.810	1.476	No	ns	-8.540 to 2.919
3.2 µM vs 8 µM	3.492	1.834	No	ns	-2.237 to 9.222
3.2 µM vs 20 µM	0.1540	0.08087	No	ns	-5.576 to 5.884
8 µM vs 20 µM	-3.338	1.753	No	ns	-9.068 to 2.391

S91. Statistical analysis of the cytotoxicity of derivative **11** in Hs27 cells
